# “DEPHENCE” system—a novel regimen of therapy that is urgently needed in the high-grade serous ovarian cancer—a focus on anti-cancer stem cell and anti-tumor microenvironment targeted therapies

**DOI:** 10.3389/fonc.2023.1201497

**Published:** 2023-06-28

**Authors:** Jacek R. Wilczyński, Miłosz Wilczyński, Edyta Paradowska

**Affiliations:** ^1^ Department of Gynecological Surgery and Gynecological Oncology, Medical University of Lodz, Lodz, Poland; ^2^ Department of Gynecological, Endoscopic and Oncological Surgery, Polish Mother’s Health Center—Research Institute, Lodz, Poland; ^3^ Department of Surgical and Endoscopic Gynecology, Medical University of Lodz, Lodz, Poland; ^4^ Laboratory of Virology, Institute of Medical Biology of the Polish Academy of Sciences, Lodz, Poland

**Keywords:** high-grade serous ovarian cancer, ovarian cancer stem cells, metastatic niche, tumor microenvironment, chemo-resistance, experimental therapy, clinical trial

## Abstract

Ovarian cancer, especially high-grade serous type, is the most lethal gynecological malignancy. The lack of screening programs and the scarcity of symptomatology result in the late diagnosis in about 75% of affected women. Despite very demanding and aggressive surgical treatment, multiple-line chemotherapy regimens and both approved and clinically tested targeted therapies, the overall survival of patients is still unsatisfactory and disappointing. Research studies have recently brought some more understanding of the molecular diversity of the ovarian cancer, its unique intraperitoneal biology, the role of cancer stem cells, and the complexity of tumor microenvironment. There is a growing body of evidence that individualization of the treatment adjusted to the molecular and biochemical signature of the tumor as well as to the medical status of the patient should replace or supplement the foregoing therapy. In this review, we have proposed the principles of the novel regimen of the therapy that we called the “DEPHENCE” system, and we have extensively discussed the results of the studies focused on the ovarian cancer stem cells, other components of cancer metastatic niche, and, finally, clinical trials targeting these two environments. Through this, we have tried to present the evolving landscape of treatment options and put flesh on the experimental approach to attack the high-grade serous ovarian cancer multidirectionally, corresponding to the “DEPHENCE” system postulates.

## Introduction

Ovarian cancer, especially its type II according to the dualistic model proposed by Kurman and Shih (1), represented mostly by the high-grade serous ovarian cancer (HGSOC) is the most lethal tumor of the female genital tract. The cumulative 5-year survival in the population of patients with all clinical stages does not exceed 48% (2). Despite the fact that some cases of the HGSOC are primarily chemo-refractory, the most of the cancers belonging to this group are chemosensitive to first-line chemotherapy; however, they quickly acquire the secondary chemoresistance that constitutes the main problem in effective management. Moreover, the HGSOC possesses unique behavior that allows spreading of the tumor cells, mostly in the form of cellular spheroids, from the primary tumor into the distant localizations in the peritoneal cavity. Therefore, the HGSOC is a highly malignant, rapidly progressive tumor characterized by poor prognosis and mortality reaching 90% of all ovarian cancer cases (3).

## Ovarian cancer stem cells

One of the main problems in the treatment of HGSOC is the existence of ovarian cancer stem cells (OCSCs) that reside inside tumor niches and cooperate with surrounding cells that compose tumor microenvironment (TME). The character of this cooperation shapes tumor behavior and influences several processes including dormancy, proliferation, metastasis, and, most of all, chemoresistance (4). Cancer stem cells are a population of cells capable of self-renewal and reproduction of the original phenotype of the tumor and are enriched especially in the advanced, disseminated, and recurrent tumors (5). There are two functionally distinct populations of CSCs, proliferating and quiescent, which occupy different niches inside the tumor. The proliferative CSCs are chemoresistant but vulnerable to overdoses of the chemotherapeutics; however, quiescent CSCs are in the autophagic state and could survive even high doses of anti-cancer drugs, thus enabling tumor relapse (6). One of the key phenomena responsible for regulation of stemness is epithelial–mesenchymal transition (EMT) viewed as a continuum of phenotype cellular states from complete epithelial and proliferative state, through several intermediate hybrid states to complete mesenchymal and invasive phenotype. Cancer stem cells could represent any of these steps due to the outstanding plasticity (7). This plasticity of CSCs is highly dependent on the patient’s immunosurveillance as well as on epigenetic and environmental signals from the TME (6). The most recognized stressors that could influence both phenotype and function of CSCs are hypoxia, acidity, mechanical stress, immunological response, epigenetic changes like DNA methylation, histone and non-coding RNA modifications, and, finally, activation of stemness signaling pathways (8–11).

The problem of the stemness is directly connected to the cancer dormancy that is dependent on the presence of circulating tumor cells (CTCs) and disseminated tumor cells (DTCs) that have partially overlapping functions and are enriched by the population of quiescent CSCs (12). CTCs, DTCs, and CSCs are able to produce micrometastases that migrate and home inside the target organs in the pre-metastatic niches composed from tumor cells and recruited local stromal and immune cells from the environment. Quiescent CSCs and dormant DTCs inside pre-metastatic niche show overexpression of signaling pathways, enabling them to survive in stressful conditions, including chemotherapeutics (13–15).

Ovarian CSCs are characterized by cell surface CD44, CD117, CD133, CD24, MyD88, epithelial cell adhesion molecule (EpCAM), leucine-rich repeat containing G protein–coupled receptor-5 (LGR5), and LGR6] and intracellular [aldehyde dehydrogenase (ALDH), sex determining region Y-box 2 (SOX2), octamer-binding transcription factor-4 (OCT4), homeobox protein NANOG transcription factor (NANOG), and forkhead box protein M1 (FOXM1)] markers, as well as by their specific behavior (“side-population” cells). The markers for characterization of OCSCs, their function, and clinical significance are presented in **Table 1**. The OCSC markers unfortunately are not cancer stem cell specific, as they are also present on normal stem cells. Another feature of OCSCs is activation of signaling pathways upregulating their stemness, cancer proliferative capability, and chemoresistance. The most important and studied pathways for preservation of OCSC function are Wnt/β-catenin, Hedgehog, Hippo/yes-associated protein (YAP), neurogenic locus notch homolog (NOTCH), nuclear factor-κ-light chain enhancer of activated B cells (NF-κB), hypoxia-induced factor-1α (HIF-1α), PI3K/protein kinase B (AKT), Janus kinase (JAK)/signal transducer and activator of transcription protein (STAT), transforming growth factor–β (TGF-β), and Rho/Rho-associated protein kinase (Rho/ROCK) pathways. The functional and clinical characterization of these pathways is included in **Table 2**.

**Table 1:** The markers for characterization of OCSCs, their function, and clinical significance.

OCSC marker	Function	Clinical significance	Reference
**CD44** **CD44 spliced variant 6 (CD44v6)**	Cell-surface glycoprotein, receptor for the hyaluronic acid receptor Activates EGFR/Ras/ERK and NANOG-dependent signaling pathways Resulting NANOG/STAT3 interaction upregulates multi-drug resistance CD44+ cells possess self-renewal, tumor-initiating and sphere-forming capability	High number of CD44+ cells in early stage HGSOC correlated with shorter PFS Expression correlated with advanced HGSOC, p53 positivity, tumor grade, and chemoresistance CD44+ cells are overrepresented in recurrent compared to primary HGSOC Increased CD44v6+ cell numbers in primary ovarian tumors correlated with shorter OS CD44v6+ cells are overrepresented in metastases Distant metastases-free survival is better in patients with CD44v6-low tumors Population of CD44+CD166+ cells is abundant in platinum-resistant ovarian cancer	(16–24)
**CD117**	Receptor tyrosine kinase coded by c-kit proto-oncogene Regulates cell proliferation, differentiation, apoptosis, and adhesion Binding CD117 to SCF is followed by activation of several pathways, including Ras/ERK, PI3K/AKT, and Src/JAK/STAT SCF produced by TAMs and CAFs is highly expressed in ascites of patients with HGSOC CD117+ cells are abundant in a sphere-forming non-adherent OCSCs and the “side population” OCSCs with increased capacity of self-renewal, tumorigenicity, and chemoresistance, as well as selective expression of ABC transporters	CD117+ cells correlate with chemoresistance shorter OS, and shorter DFI CD117+ expression present in HGSOC and correlated with chemoresistance CD44+/CD117+ cells are abundant in chemoresistant HGSOC and cell lines resistant to paclitaxel-induced apoptosis	(25–30)
**CD133**	Prominin-1 is a transmembrane glycoprotein Activates PI3K/AKT pathway Is responsible for tumorigenicity, vascularization and chemoresistance Cooperation between CD133 and ETRA augments sphere-forming capacity and homing to peritoneal surface NF-κB and p38/MAPK-dependent pathways enhance self-renewal of CD133+ cells	CD133+ cells are more abundant in recurrent chemoresistant tumors Expression of CD133+ correlated with the HGSOC type, stage, ascites, and chemoresistance CD133+ cells correlated with shorter PFS and OS CD133+/CXCR4+ cells are more platinum-resistant compared to CD133 negative cells	(31–37)
**CD24**	Heat-stable antigen CD24, a transmembrane adhesion molecule Activates JAK/STAT3 signaling pathways and NANOG and OCT4 expression Through stimulation of PI3K/AKT/MAPK pathway is able to stimulate EMT Stimulates stemness, tumor growth, and chemoresistance CD24+ cells form spheroid structures Exosomes present in ascites contain CD24 and EpCAM, which regulate signals between OCSCs and TME	CD24+ cells are abundant in peritoneal implants compared to primary tumor Inhibition of JAK/STAT3 pathway reduces OCSC stemness and improves patient’s survival CD24+ expression is a predictor of poor outcome in patients with ovarian cancer Expression of CD24+ correlates with cancer stage and presence of peritoneal implants and metastases	(29, 38–43)
**EpCAM**	Epithelial cell adhesion molecule is a type I transmembrane glycoprotein regulating intercellular adhesion EpCAM+ cells show greater tumor-initiating potential EpCAM/Bcl-2 pathway prevents platinum-dependent apoptosis of cancer cells EpCAM+CD45+ cells constitute the chemoresistant phenotype in the ascitic fluid of patients with ovarian cancer. These cells overexpress *SIRT1*, *ABCA1*, and *BCL2* genes. EpCAM+CD45+ population is highly invasive with signature mesenchymal gene expression and also consists of CD133+ and CD117+CD44+ OCSCs	EpCAM expression is increased in chemoresistant tumors and correlated with poor outcome EpCAM+ cells are a source of relapse after the chemotherapy	(20, 44, 45)
**MyD88**	Myeloid differentiation primary response gene 88 is an adaptor protein for signals generated from TLR-4 receptor TLR-4/MyD88 pathway is responsible for chemoresistance and activates inflammatory pathways in carcinogenesis	Expression of MyD88 is an unfavorable prognostic factor in ovarian cancer CD44+/MyD88+ cells show increased tumorigenicity, sphere formation, and chemoresistance	(28, 46)
**LGR5 and LGR6**	Leucine-rich repeat containing G protein–coupled receptor-5 and receptor-6 are biomarkers of adult stem cells Expression of LGR5 promotes proliferation and metastasis, and EMT in ovarian cancer LGR5- and LGR6-mediated signaling is responsible for activation of Wnt/β-catenin pathway in OCSCs	LGR5 expression is correlated to ovarian tumor stage and histologic grade LGR6 expression and activation of Wnt/β-catenin pathway are observed in tubal fimbria of patients with HGSOC	(47, 48)
**ALDH**	Aldehyde dehydrogenases are aldehyde-converting enzymes ALDH1 subgroup of enzymes is engaged in protection of cancer cells against chemotherapy and radiation ALDH1A1 and ALDH1A2 are the most popular phenotypes found in OCSCs ALDH1 activates the Wnt/β-catenin pathway and transmembrane transporters	Ovarian cancer cells pre-treated with growing doses of platinum show increased number of ALDH1+ cells HGSOC cells showing ALDH1+/EGFR+ phenotype are correlated with the worse prognosis High expression of ALDH1+ cells correlates with chemoresistance CD44+/CD133+/ALDH1A1+ cells are increased in recurrent tumors Expression of CD133+/ALDH1+ correlates with shorter PFS and OS in HGSOC	(49–52)
**NANOG**	Homeobox protein NANOG transcription factor Regulates self-renewal and pluripotency of embryonic and CSCs cells, and EMT Through STAT3 signaling pathway upregulates chemoresistance	Expression of NANOG+ cells correlates with clinical stage, histologic grade, and chemoresistance	(17)
**SOX2**	Sex determining region Y-box 2 transcription factor Regulates self-renewal and pluripotency of embryonic and CSCs cells Overexpression of SOX2 enhances stemness by inhibition of PI3K/AKT signaling pathway	Highly SOX2+ cells are present in epithelium of tubal fimbriae in HGSOC and BRCA1/2+ patients	(53, 54)
**OCT4**	Octamer-binding transcription factor-4 is engaged in self-renewal of undifferentiated embryonic stem cells Stabilizes structure of chromatin in the NANOG locus Cytoplasmic expression of OCT4 regulates EMT Mechanosensory signals in the peritoneum stimulate EMT; enhance stemness by upregulation of OCT4, CD44, and CD117; and increase chemoresistance	Upregulation of OCT4 in ovarian cancer is correlated to chemoresistance Increased expression of OCT4 is observed in CD24+ OCSC cells	(40, 55–57)
**FOXM1**	Forkhead box protein M1 is a member of the FOX family of transcription factors Regulates cell cycle and controls genomic stability Upregulation of FOXM1 is followed by activation of Wnt/β-catenin signaling pathway and enhances chemoresistance	Overexpression of FOXM1 is observed in OCSCs exposed to LPA present in ascites FOXM1 deactivation results in restoration of chemosensitivity and loss of ability to spheroid creation in peritoneum	(58–60)
**MSH-1/MSH-2**	MSH proteins regulate stemness of OCSCs and are aberrantly expressed in tumors. MSH proteins activate the NOTCH signaling	High expression of MSH proteins is correlated to shorter OS and enhances paclitaxel chemoresistance	(61–64)

CD, cluster of differentiation; EGFR, epidermal growth factor receptor; Ras, Ras small GTPase protein; ERK, extracellular signal–regulated kinase; NANOG, homeobox protein NANOG transcription factor; STAT3, signal transducer and activator of transcription 3; PFS, progression-free survival; OS, overall survival; SCF, stem cell factor; PI3K, phosphoinositide-3-kinase; AKT, protein kinase B; Src, non-receptor tyrosine kinase Src; JAK, Janus kinase; TME, tumor microenvironment; TAMs, tumor-associated macrophages; CAFs, cancer-associated fibroblasts; ABC transporters, ATP-binding cassette drug membrane transporters; DFI, disease-free interval; ETRA, endothelia receptor A; NF-κB, nuclear factor-κ-light chain enhancer of activated B cells; p38/MAPK, p38 mitogen-activated protein kinase; CXCR4, C-X-C chemokine receptor type-4; OCT4, octamer-binding transcription factor-4; EMT, epithelial–mesenchymal transition; Bcl-2, B-cell lymphoma-2 molecule; TLR-4, Toll-like receptor type-4; LGR5, leucine-rich repeat containing G protein–coupled receptor-5; ALDH, aldehyde dehydrogenase; Wnt, wingless and Int-1; SOX2, sex determining region Y-box 2; FOXM1, forkhead box protein M1; LPA, lysophosphatidic acid; SIRT1, sirtuin-1; BCL2, B-cell lymphoma-2; MSH, Musashi protein; NOTCH, neurogenic locus notch homolog.

**Table 2:** The functional and clinical characterization of ovarian cancer stem cell signaling pathways.

Pathway	Function	Clinical significance	Reference
**Wnt/β-catenin signaling**	Activation of ABC transmembrane transporter member-2 (ABCG2) Cooperation with LGR5 and LGR6 stemness markers for activation of the pathway in OCSCs Regulation of EMT through increase of the SNAIL/E-cadherin ratio and enhancement of OCSC motility and chemoresistance Promotion of pro-inflammatory and tumor-supporting phenotype of CAFs	Knock-down of β-catenin restores chemosensitivity in ovarian cancer cells Restoration of SFRP5 function inhibits Wnt/β-catenin signaling, EMT and re-establishes chemosensitivity Chemoresistant HGSOC tumors show the upregulation of Wnt/β-catenin-dependent target genes and OCSC markers	(48, 59, 65–67)
**Hedgehog signaling**	Activation of this pathway in OCSCs stimulates chemoresistance and spheroid formation Proteins GLI1 and SMO are the downward effectors of Hedgehog signaling GLI1 upregulates function of ABCB1 and ABCG2 transporters, thus supporting chemoresistance Hedgehog signaling is important for the interaction between OCSCs and CAFs	Malignant and chemoresistant ovarian tumors show increased expression of GLI1 and SMO compared to benign and chemosensitive tumors	(68–70)
**Hippo/YAP signaling**	Pathway is activated by stiffness of ECM and shear stress in OCSC environment (mechanosensory signals) Hippo/YAP pathway stimulates proliferation, metastasis, and chemoresistance in ovarian cancer Overexpression of YAP promotes EMT and cancer cell migration while inhibiting cells’ anoikis Increase in the OCSC pool results from the activation of the Hippo pathway target genes upon MYPT1 downregulation	YAP expression is an indicator of poor prognosis in ovarian cancer Expression of the key genes related to Hippo/YAP signaling is correlated with PFS	(71–74)
**NOTCH signaling**	The NOTCH receptors are a membrane receptor proteins responsible for proliferation of cells and angiogenesis of the tumor NOTCH overexpression is observed in ovarian cancer, together with the downstream components of this pathway, like VEGF, VEGFR1, DLL4, and JAG1 Tumor hypoxia enhances NOTCH signaling, stemness, and migration of OCSCs NOTCH pathway upregulates NANOG, OCT4, and ABCB1 transporter, thus increasing chemoresistance	High activity of NOTCH pathway is found in paclitaxel-resistant ALDH1+ OCSCs High expression of NOTCH is correlated with poor OS and DFS, and advanced or recurrent cancer Inhibition of NOTCH signaling restores chemosensitivity	(75–77)
**NF-**κ**B signaling**	NF-κB is a protein complex functioning as a transcription factor responsible for cellular response towards stress, cytokines, reactive oxygen species, antigens, and inflammation Inhibition of NF-κB signaling induces apoptosis, restores chemosensitivity, and decreases the CD44+ OCSC population Cooperation between CAFs and OCSCs results in the upregulation of NF-κB signaling in OCSCs Inflammatory signals from the tumor environment enhance NF-κB pathway and stemness of cancer cells NF-κB over-activity characterizes CD44+MyD88+ OCSCs A matrix protein periostin expressed in highly aggressive ovarian cancer is involved in NF-κB–mediated over-activity of M2 macrophages and CAFs that promote tumor growth The TLR4/NF-κB/HIF-1α signaling loop promotes progression of ovarian cancer	Overexpression of NF-κB is correlated with chemoresistance and poor prognosis in HGSOC Expression of NF-κB is correlated to high stage and grade of ovarian cancer NF-κB mediates the BRCA1-induced chemoresistance Signal transduction pathway activity analysis of HGSOC revealed that the low activity of PI3K together with the high activity of NF-κB pathway has favorable prognosis and indicates more active immune response, whereas the high PI3K and the low NF-κB pathway activity has poor prognosis and indicates high cell proliferation	(78–86)
**HIF-1**α** signaling**	Hypoxic environment is followed by increased expression of transcription factor HIF-1α HIF-1α signaling activates EMT and stemness-regulating pathways, like Wnt/β-catenin, Hedgehog, and NOTCH while downregulating NF-κB pathway HIF-1α signaling upregulates OCSC markers CD133, NANOG, and SOX2 SIRT1 is a downstream target gene for the HIF-1α pathway, involved in promotion of OCSCs by hypoxia, and NF-κB signaling cooperates with HIF-1α in SIRT1 upregulation	Patients with higher expression of HIF-1α have shorter OS Patients with III stage of platinum-resistant HGSOC has overrepresentation of HIF-1α signaling pathway	(87–90)
**PI3K/AKT signaling**	CAFs stimulate ovarian cancer invasiveness and chemoresistance through the activation of HGFR/PI3K/AKT signaling Interaction with MSCs leads to activation of PI3K/AKT pathway and MDR proteins in OCSCs Defective function of *CTNNB1*, *PTC*, *SMO*, *NOTCH*, *k-Ras*, and *MEK* genes disturbs between others the function of PI3K/AKT pathway in OCSCs Loss of *BRCA* expression is also followed by activation of PI3K/AKT pathway in OCSCs PI3K/AKT/mTOR pathway is activated by abundance of nutrients and growth factors in TME that inhibits autophagy in cancer cells Activation of PI3K/AKT/mTOR signaling results in upregulation of CD44v6, CD117, and ALDH1A1 OCSC markers as well as enhancement of EMT in chemo resistant ovarian cancer cell lines Shear stress exerted by ascites together with HGF stimulate stemness and chemoresistance by HGFR/PI3K/AKT-miR-199a-3p pathway Blocking CXCR4/PI3K/AKT/mTOR signaling results in reduction of OCSCs and inhibition of EMT Circular RNA circ_0000745 upregulated by IGF2BP2 stimulates stemness of ovarian cancer cells through a miR-3187-3p/ERBB4/PI3K/AKT pathway	High expression of PI3K or AKT is correlated to shorter OS in ovarian cancer UBE2S is a potential oncogene that, through stimulation of PI3K/AKT/mTOR pathway, enhances proliferation and migration of ovarian cancer. Its high expression is a poor prognostic factor Inhibition of PI3K in wild-type *PI3KCA* ovarian cancer induces *BRCA* downregulation and with PARP inhibitors shows synergistic effect against ovarian cancer	(91–103)
**JAK/STAT signaling**	Inhibition of JAK2/STAT3 signaling results in decrease of stemness and reduced tumor growth Inhibition of JAK/STAT pathway in HGSOC cells and CAFs has anti-tumor activity OCT4 accelerates tumor growth and enhances chemoresistance through activation of JAK/STAT pathway in OCSCs represented by “side population” LIF and IL-6 secreted by MSCs promote OCSC function by STAT3 signaling	Higher expression of STAT3 is correlated to poor prognosis in ovarian cancer	(38, 104–107)
**TGF-**β** signaling**	TGF-β is one of the pro inflammatory cytokines secreted by CAFs in the OCSC niche Spheroid cancer cells through secretion of TGF-β force mesothelial epithelium to home cancer implants TGF-β secreted by CAFs and TILs stimulates epigenetic changes that promote EMT Enrichment of OCSC population by MSCs is mediated by TGF-β Upregulation of TGF-β together with VEGF and HIF-1α enhances angiogenesis and stemness OCSCs convert the immature DCs into the TGF-β–secreting cells, which support the expansion of Treg lymphocytes defending the tumor TGF-β induces the population of pro-angiogenic N2-polarized TANs that support tumor growth and vascularization miR-33a-5p through the downregulation of CROT and activation of TGF-β signaling promotes tumor growth and paclitaxel resistance	miR-506 prevents TGF-β–induced EMT. Ovarian tumors showing increased miR-506 expression correlated with better prognosis for the patients *STMN2* and *RAD51AP1* genes overexpression are correlated with poor prognosis in HGSOC and associated with TGF-β signaling pathway TGF-β–induced stimulation of CAF-derived periostin secretion is correlated with reduced survival in HGSOC Seven unfavorable genotypes associated with regulation of TGF-β–mediated signaling are correlated to shorter OS and PFS in patients with ovarian cancer NR2F1 that reveals a high correlation with poor prognosis and tumor stage regulates EMT and immunosuppressive CAFs infiltration through TGF-β signaling	(84, 108–119)
**Rho/ROCK signaling**	ECM stiffness and tissue tension exerted by ascites activate Rho/ROCK pathway and regulate EMT Rho/ROCK signaling is an important mediator in tumor angiogenesis Rho/ROCK pathway is used by invading cell clusters and “leader cells”	Pharmacological inhibition of LPA-mediated stimulation of Rho/ROCK pathway decreases tumor aggressiveness Inhibition of Rho/ROCK pathway blocks HIF-1α signaling and restores platinum sensitivity of ovarian cancer cells	(120–124)

SNAIL, zinc-finger transcription factor SNAI1; SFRP5, secreted frizzled-related protein-5; CAFs, cancer-associated fibroblasts; GLI1, zinc-finger protein GLI1; SMO, smoothened class frizzled G protein–coupled receptor; YAP, yes-associated protein; ECM, extracellular matrix; EMT, epithelial–mesenchymal transition; MYPT1, myosin phosphatase targeting protein-1; PFS, progression-free survival; VEGF, vascular-endothelial growth factor; VEGFR1, VEGF receptor-1; DLL4, delta-like ligand-4; JAG1, protein Jagged 1; NANOG, homeobox protein NANOG transcription factor; OCT4, octamer-binding transcription factor-4; ABC transporter, ATP-binding cassette drug membrane transporter; OS, overall survival; DFS, disease-free survival; NF-κB, nuclear factor-κ-light chain enhancer of activated B cells; TLR-4, Toll-like receptor type-4; HIF-1α, hypoxia-induced factor-1α; BRCA1, breast cancer type-1 susceptibility protein; SIRT1, sirtuin type-1; HGFR, hepatocyte growth factor receptor; MSCs, mesenchymal stem cells; MDR, multi-drug resistance; CTNNB1, catenin beta-1; PTC, papillary thyroid cancer oncogene; SMO, smoothened protein-coding gene; NOTCH, NOTCH receptor-coding gene; k-RAS, Kirsten rat sarcoma virus gene; MEK, mitogen-activated protein kinase kinase-1 coding gene; TME, tumor microenvironment; HGF, hepatocyte growth factor; mTOR, mammalian target of rapamycin kinase; CXCR4, C-X-C chemokine receptor type-4; UBE2S, ubiquitin-conjugating enzyme E2S; PI3KCA, phosphatidylinositol-3-kinase oncogene; PARP, poly-ADP ribose polymerase; TGF, transforming growth factor; TILs, tumor-infiltrating lymphocytes; DCs, dendritic cells; TANs, tumor-associated neutrophils; STMN2, stathmin-2 gene; CROT, carnitine O-octanoyltransferase; RAD51AP1, RAD51-associated protein-1 gene; Rho/ROCK, Rho/Rho-associated protein kinase; LPA, lysophosphatidic acid.

## Tumor microenvironment in ovarian cancer

Ascites is a unique microenvironment for OCSCs and is responsible for exceptional biology of ovarian cancer, shaped by the transcoelomic spread of peritoneal implants. The EMT process enables the tumor cells from primary localization to seed in the form of multiform cellular conglomerates, mostly adopting the form of spheroids enriched in OCSCs. They are transported in fluid into distant places of peritoneal cavity, with the predisposition to home into the adipose tissue collections inside peritoneum, like “milky spots”, omental fat, mesentery, or bowel appendices (125). Sphere-forming cells express OCSC markers CD44v6, CD117, ALDH1, and NANOG and are resistant to anoikis despite lack of anchorage to the surface (16, 126). The presence of cytokines [interleukin-6 (IL-6), IL-8, IL-10, and vascular endothelial growth factor (VEGF)], osteoprotegerin, and exosomes containing micro RNAs (miRNAs), cytokines, and growth factors further enhances stemness in the spheroids (38, 68, 127–129). Spheroids adhere to and destroy the mesothelium, go through the mesenchymal/epithelial transition, and start to proliferate (130, 131). TGF-β, CD133, and CD44 from spheroids stimulate mesothelium to produce fibronectin for cancer cells adhesion, enhance attachment of floating cells to the epithelial surface, and stimulate secretion of metalloproteinase-9 (MMP-9) that supports mesothelial invasion (108, 132). The initial opinions on random transportation of cellular conglomerates have been replaced by the theory of collective invasion, according to that clusters of cancer cells migrate in a directed and coordinated way (133). Collective invasion is described by some characteristic features, mainly preservation of cell-cell junctions, interaction with surface cells and ECM on their way, cooperative cytoskeleton dynamics enabling migration of clusters as a single unit, and multicellular polarity (120, 133–135). Despite a collective behavior, not all cells in the cluster are invasion-competent, and the population of cells that rule invasion is called “leader cells”. These cells delineate the way, change cellular contractility, and grow invadopodia, as well as respond to environmental signals (120, 136–138). Their presence at the front of the cluster results in its polarization. The coordinated movement requires rearrangement of the cytoskeleton, actinomyosin contraction, and activation of PI3K and Rho/ROCK pathways (120, 135, 139). After adhesion to the mesothelial surface, “leader cells” express proteolytic enzymes and penetrate the basement membrane (120, 140). The phenotype of “leader cells” is characterized by the keratin-14 (KRT14) expression. Their functional phenotype resembles the OCSC phenotype but does not correlate to EMT. The KRT14+ cells are able to re-establish the epithelial cells, show clonogenicity, are abundant in metastases, are enriched in response to chemotherapy, and promote the chemoresistance (120, 140–143). Cancer-associated fibroblasts (CAFs) present in TME play important role in collective invasion by regulation of TME remodeling to “pave” the routs for migrating cell clusters (120, 144). After exposition to chemotherapy, the population of apoptosis-resistant “leader cells” increases and shows expression of ALDH1 and CD44v6 stemness markers together with chemoresistance. Functional impairment of the “leader cells” restores chemosensitivity *in vitro* (145). After homing into peritoneal environment, OCSCs reside inside the “metastatic niche” composed of several cell populations, ECM components, lipids, exosomes, regulatory RNAs, and hypoxia that are orchestrated to support the OCSCs. **Table 3** presents the function and clinical significance of the main components of the TME inside the “metastatic niche”.

**Table 3:** The function and clinical significance of the main components of the tumor microenvironment inside the “metastatic niche”.

TME component	Function	Clinical significance	Reference
**CAFs**	CAFs originate from peritoneal fibroblasts or MSCs activated by inflammatory signals, hypoxia, and exosomes produced by cancer cells Activated CAFs secrete TGF-β that stimulates EMT and metastases Increased expression of DKK3 protein enhances Hippo/YAP and Wnt/β-catenin signaling in CAFs thus supporting OCSCs CAFs enhance chemoresistance by activation of HGFR/PI3K/AKT pathway FGF secreted by CAFs stimulates VEGF secretion and OCSC stemness TME remodeling by secretion of ECM components and MMPs Suppression of cytotoxic TILs and enhancement of pro-inflammatory signals The existence of the functional loop between CAFs and ovarian cancer cells is reported, in which CAFs induce angiogenesis by secretion of IL-6, COX-2, and CXCL1, whereas cancer cells induce CAFs to secrete CXCL12, IL-6, and VEGF-A to further enhance angiogenesis	Four genes—*AXL*, *GPR176*, *ITGBL1*, and *TIMP3*—identified as ovarian cancer CAF-specific genes allow to construct the prognostic CAF signature. High CAF signature correlates to chemoresistance and activation of signaling pathways regulating tumor progression Molecular CAF signature characterized by the expression of six CAF-related genes (*COL16A1*, *COL5A2*, *GREM1*, *LUM*, *SRPX*, and *TIMP3*) show that high-risk patients have worse prognosis, ineffective immune response, and low tumor mutational burden CAF-score based on molecular characteristics of CAF-related genes and signaling pathways allows classifying patients with ovarian cancer to high- or low-risk population. Higher CAF score is observed in advanced tumors and in patients with worse OS. Patients with low CAF score have better efficacy of immunotherapy CAFs mediate chemoresistance of ovarian cancer to anti-angiogenic therapy	(91, 110, 146–157)
**CAAs**	Adipocytes are a source of lipids but also secrete adipokines, growth factors, immune mediators, and metabolic agents Omental implants are an example of OCSC niche supporting energetically and proliferatively stem cells Recruitment of OCSCs into the adipose tissue depends on IL-6, IL-8, MCP-1, and TIMP1 Interaction between IL-8 secreted by CAAs and CXCR1 on cancer cells activates metastases through p38MAPK/STAT3 pathway Lipid transfer from CAAs to cancer cells depends on FABP4, which is upregulated especially in metastatic tumors ALDH+CD133+ OCSCs show high levels of desaturation of lipids Survival of OCSCs in adipose tissue TME depends on the function of SCD1, and elimination of SCD1 is synonymous with OCSC depletion Fatty acids supply energy for EMT	High levels of fatty acids desaturation and oxidation in FABP4-positive tumors correlate with poor prognosis FASN expression correlates with stage and grade of ovarian cancer, and patients showing high FASN expression have worse prognosis and chemoresistant tumors	(158–164)
**ADSCs**	ADSCs promote generation of OCSCs with use of Hedgehog/BMP4 signaling. Through secretion of IL-6, IL-8, VEGF, and TNF-α, ADSCs enhance chemoresistance. They are capable to differentiate into CAFs and CAAs		(165–167)
**MSCs**	MSCs are recruited from bone marrow, adipose tissue, and endometrium and are able to differentiate into CAFs MSCs stimulate proliferation, stemness, angiogenesis, and platinum resistance IL-6 and LIF secreted by MSCs enhance OCSC function in the STAT-dependent way MSC-derived TGF-β and VEGF/HIF-1α signals contribute to OCSC support and angiogenesis Bone marrow MSCs enhance chemoresistance of ovarian cancer by releasing miR-1180 that activates Wnt/β-catenin signaling	Interactions with MSCs activate PI3K/AKT pathway and MDR in OCSCs followed by paclitaxel and platinum resistance	(94, 106, 111, 147, 166, 168–171)
**TAMs**	Conversion of monocytes into TAMs is triggered by LIF and IL-6 present in ascites TAMs residing inside “metastatic niche” show immunosuppressive M2 phenotype and take part in immune escape of the tumor, regulation of angiogenesis, invasion, and stemness Hypoxia in ovarian cancer TME shifts polarization of TAMs into M2 phenotype through miR-222-3p and miR-940 released from cancer cells and activation of STAT pathway Another signal for M2 differentiation of TAMs are cytokines IL-4, IL-10, and IL-13 secreted from both cancer and MSC cells By secretion of pro-inflammatory IL-17, TAMs stimulate p38MAPK and NF-κB pathways that induce self-renewal of CD133+ OCSCs TAMs secrete VEGF and EGF that induce spheroid formation and peritoneal spread of cancer implants M2-type TAMs create and support tumor tolerance by inhibition of NK and cytotoxic T-cell activity and by stimulation of Tregs	Patients with higher M1/M2 TAMs ratio have better OS and PFS M2 TAM infiltration is correlated to worse OS	(32, 172–182)
	UBR5-mediated immunosuppressive TAM infiltration augments tumor growth and metastases and, through activation of p53/β-catenin/CCL2 pathway, stimulates spheroid formation *CAPG* gene expression is correlated with infiltration of tumors by Tregs, M2 TAMs, and exhausted T cells contributing to immunosuppression in HGSOC TAMs exert pro-tumor and immunosuppressive effects through secretion of IL10,TGFβ, VEGF, and expression of PD-1 and consumption of arginine to inhibit T-cell efficacy		
**CD4+CD25+FoxP3+ Tregs**	Expression of suppressive molecule IDO by cancer and dendritic cells contributes to recruitment of Tregs into the tumors Tregs from ovarian tumors show upregulation of TGF-β that inhibits secretion of IL-2, IFN-γ and TNF-α followed by impairment of T CD4+ and T CD8+ effector cells Ovarian OCSCs through CCL5–CCR5 interaction recruit Tregs, which, upon culture with CD133+ OCSCs, secrete high levels of IL-10 showing inhibitory immune function and MMP-9 that enables invasion of cancer cells Tregs infiltrating ovarian tumors show highly activated phenotype (PD-1, 4-1BB, and ICOS) responsible for immunosuppression	High numbers of Tregs in tumor immune infiltrates are considered a sign of poor prognosis T CD8+/Tregs and CD4+/Tregs ratio are a good predictors of patient survival Abundance of Tregs and increased VEGF in ascites are observed in patients with poor prognosis However, the prognostic value of Tregs depends on the tumor type and stage, and, in HGSOC tumors, lower Th17/Tregs ratio was correlated with better survival	(183–190)
**mDCs and pDCs**	Tumor and ascites DCs originate from peripheral blood mDCs express IDO and PD-1 and are associated with mmunosuppression of anti-cancer T CD4+ helper and T CD8+ cytotoxic effectors Tumor growth is accompanied by increasing numbers of mDCs, and tumor-derived PGE2 and TGF-β further promote the function of mDCs	The correlation between higher concentration of tumor- associated pDCs and shorter PFS was found The presence of mature DCs correlates with improved prognosis in HGSOC	(191–193)
	Immature mDCs are capable to regulate angiogenesis in the tumor pDCs accumulate preferentially in ascites and their chemoattraction depends on expression of CXCL12 pDCs stimulate the generation of IL-10+ T CD8+ suppressor cells and promote angiogenesis through the secretion of IL-8 and TNF-α The population of tumor-associated pDCs differs functionally from ascitic pDCs and secretes lower levels of pro-inflammatory cytokines		(187, 194–197)
**MDSCs**	MDSC cells possessing CD11b+/Gr-1+ phenotype are a cell population regulating both chronic inflammation and tumor progression MDSCs are able to suppress maturation of DCs and cytotoxic reactions against tumor mediated by T CD8+, NK, and NKT cells Recruitment and functional maturity of MDSCs in the ascites depend on CXCL12/CXCR4 interactions and PGE2 secretion IL-6 and IL10 in ascites increase the number of MDSCs and, through upregulation of STAT3 signaling, promote their suppressive activity by expression of ARG and iNOS Inhibition of mTOR activity decreases MDSC infiltration of ovarian tumors and slows progression PGE2 produced by MDSCs enhances expression of PD-L1 through mTOR pathway. PD-L1 expression is particularly high in OCSCs having ALDH1+ phenotype	Blockade of a key cytokine for MDSCs function, IL10, restores immunosurveillance and improves survival Peripheral blood ARG/IDO/IL10+ MDSCs are especially abundant in patients with advanced ovarian cancer and their depletion is a good prognostic factor BRCA-mutated patients have less MDSCs and more T CD*+ effectors than patients with wild BRCA copy in early stage HGSOC, what could explain partly the survival benefit in this group of patients	(198–205)
**ECM**	Mechanosensory signals produced by ascites and tumor expansion regulate EMT and interaction with EMC, as well as enhance angiogenesis, stemness, and chemoresistance Shear stress stimulates stemness by increase of CD44, CD117, and OCT4 activity ECM stiffness upregulates expression of stemness CD133 marker Compression changes activity of the Wnt/β-catenin pathway and regulates EMT Expression of PAX8 links migratory and adhesive properties of Fallopian tube epithelium, STIC, and HGSOC cells. Inhibition of PAX8 reduces ability of cancer cells to migrate and adhere to fibronectin and collagen	Chondroitin sulfate is upregulated in the ECM of more than 90% of HGSOC and linked to poor prognosis Acquisition of mesothelial–mesenchymal phenotype by cancer cells, characterized by expression of CALB2 and PDPN, regulates adhesion to ECM and tumor progression and is correlated to poor outcome	(55, 130, 206–212)
**Exosomes**	Exosomes loaded with miRNAs miR-409-3p and miR-339-5p are involved in Wnt/β-catenin signaling pathway and stimulation of metastases in HGSOC Ascites contain exosomes transferring cytokines, growth factors, miRNAs, lipids, and OCSC markers CD44 and EpCAM between tumor environment and OCSCs Exosomes from cancer cells transfer CD44 into mesothelial cells stimulating MMP-9, which supports adhesion and invasion of spheroid cells to the peritoneal surface Tumor cells stimulate conversion of omental fibroblasts into CAFs by production of exosomes containing deregulated miRNAs miR-31, miR-214, and miR-155 Hypoxic environment reprograms TAMs into M2 polarization through exosomes containing miR-222-3p and miR-940 Omental CAFs and CAAs upregulate cancer cells’ chemoresistance and activate anti-apoptotic pathways through miR-21–containing exosomes MSCs enhance tumor growth producing exosomes loaded with miR-21, miR-221, and miR-92a	Exosomal miR-146a secreted from MSCs reduces cancer growth and chemoresistance to taxanes Abundance of CD117-containing small extracellular vesicles in ascites correlates with tumor grade, chemoresistance, and recurrence Higher concentration of exosomes containing miR-21, miR-141, miR-200a, miR-200b, miR-200c, miR-203, miR-205, and miR-214 is found in serum of patients with ovarian cancer compared to patients with benign ovarian tumors Expression of *LBP*, *FGG*, *FGA*, and *GSN* genes in exosomes isolated from plasma is involved in coagulation and apoptosis related pathways and can be a potential diagnostic and prognostic biomarker for OS and PFS *CAV1* gene, which is the direct target of miR-1246, is involved in the process of exosomal transfer. Patients with high miR-1246 and low Cav1 expression have a significantly worse prognosis Serum exosomal level of lncRNA MALAT1 predicts advanced and metastatic ovarian cancer phenotype and correlates to OS	(43, 172, 173, 213–226)
	Exosomes containing miR-146b-5p produced by TAMs activate TRAF6/NF-κB/MMP2 pathway that deregulates endothelial cell migration inside tumor Adipose tissue MSC-derived exosomes secreted into ascites promote tumor growth and peritoneal implants by activation of FOXM1 signaling Small extracellular vesicles released from ascites OCSCs upon cisplatin treatment are capable to activate the pro-tumorigenic phenotype in MSCs Exosomes secreted by expanded tumor-derived NK cells containing cisplatin can reverse chemoresistance of cancer cells and augment NK cytotoxic activity CD163+ TAMs secrete exosomes containing miR-221-3p that downregulates *ADAMTS6* and activate EMT, thus triggering the OCSC phenotype and chemoresistance FasL and TRAIL are components in exosomes secreted by cancer cells, responsible for apoptosis of immune cells of cancer infiltrate Ascite-derived exosomes transfer miR-6780b-5p to cancer cells promoting EMT and metastasizing CD47 is overexpressed in tumors and tumor-derived exosomes and facilitates tumor immune evasion. Inhibition of exosomal CD47 improves anti-cancer macrophage activity and suppresses peritoneal dissemination EXOSC4 is involved in RNA degradation. Knockdown of EXOSC4 inhibits the proliferation, migration, and invasion ability of ovarian cancer cells by suppressing the Wnt/β-catenin pathway	Plasma exosomal miR-1260a, miR-7977, and miR-192-5p are significantly decreased in ovarian cancer compared with healthy controls Expression level of miR-205 in plasma exosomes of the ovarian cancer group is significantly higher compared to the benign and control groups and correlates with clinical stage and lymph node metastases	(227–234)
**Hypoxia and acidosis**	Hypoxia and HIF-1α activation are capable of sustaining the CD117 expression through Wnt/β-catenin signaling Hypoxia and HIF-1α enhance stemness and EMT via activation of Wnt/β-catenin, Hedgehog, and NOTCH pathways, as well as CD133, SOX2, and NANOG markers Hypoxia/NOTCH/SOX2 signaling is important for maintaining OCSCs, as it enhances spheroid formation, upregulation of ALDH and ABC proteins, and chemoresistance Hypoxia and HIF-1α promote MDSCs to secrete TGF-β, IL-6, and IL-8 that enhance immunosuppressive conditions Hypoxia activates MAPK pathway to induce autophagy in OCSC cells Hypoxia attracts TAMs that support immune tolerance against tumor cells and predisposes mature DC cells to apoptosis Acidosis increases the expression of stemness markers OCT4 and NANOG and secretion of VEGF and IL-8 in OCSC niche Increased aerobic glycolysis in cancer cells is a source of lactate that strongly inhibits T and NK effectors, shifts TAMs into M2 phenotype, and recruits Tregs	The signature of genes associated with regulation of hypoxia and immune response allow to divide patients with ovarian cancer into high- or low-risk groups Higher *ALOX5AP*, *ANXA1*, *PLK3*, and *SREBF1* mRNA levels are significantly associated with shorter OS, whereas *LAG3* and *IGFBP2* lower mRNA levels with better prognosis, respectively Expression of seven hypoxia-related genes—*UQCRFS1*, *KRAS*, *KLF4*, *HOXA5*, *GMPR*, *ISG20*, and *SNRPD1—*divides ovarian cancer into two populations with different prognosis Hypoxia-related miR-23a-3p is overexpressed in HGSOC showing chemoresistance and shorter PFS	(96, 235–247)

TME, tumor microenvironment; CAFs, cancer-associated fibroblasts; MSCs, mesenchymal stem cells; DKK3, dickopf-related protein-3; YAP, yes-associated protein; PI3K, phosphatidylinositol-3-kinase; AKT, protein kinase B; HGFR, hepatocyte growth factor receptor; FGF, fibroblast growth factor; VEGF, vascular endothelial growth factor; ECM, extracellular matrix; MMPs, metalloproteinases; TILs, tumor-infiltrating lymphocytes; AXL, tyrosine-protein kinase receptor UFO coding gene; GPR176, G protein–coupled receptor 176 coding gene; ITGBL1, integrin subunit beta–like 1 coding gene; TIMP3, TIMP metallopeptidase inhibitor-3 coding gene; COL16A1, alpha 1 chain type XVI collagen coding gene; COL5A2, alpha 2 chain type V collagen coding gene; GREM1, Gremlin-1 protein coding gene; LUM, lumina protein coding gene; SRPX, sushi repeat containing protein X-linked coding gene; OS, overall survival; IL-6, interleukin-6; COX-2, cyclooxygenase-2; CXCL1, C-X-C motif chemokine ligand 1; CXCL12, stromal cell-derived factor 1; CAAs, cancer-associated adipocytes; MCP-1, monocyte chemoattractant protein-1; TIMP1, tissue inhibitor of metalloproteinase-1; CXCR1, C-X-C chemokine receptor type-1; FABP4, fatty acid binding protein-4; SCD1, stearoyl-CoA desaturase-1; FASN, fatty acid synthase; ADSCs, adipose-derived stem cells; BMP4, bone morphogenetic protein-4; MSCs, mesenchymal stem cells; MDR, multi-drug resistance; LIF, leukemia inhibitory factor; HIF-1α, hypoxia-induced factor-1α; TAMs, tumor-associated macrophages; p38/MAPK, p38 mitogen-activated protein kinase; EGF, epithelial growth factor; NK, natural killer; Tregs, T regulatory lymphocytes; OS, overall survival; PFS, progression-free survival; UBR5, ubiquitin protein ligase E3 component n-recognin-5; CCL2, chemokine ligand-2; CAPG, capping actin protein gelsolin-like gene; PD-1, programmed death-1; IDO, indoleamine 2,3-dioxygenase; TGF-β, transforming growth factor-β; PGE2, prostaglandin E2; CCL5, C-C motif chemokine ligand-5; CCR5, CCL5 receptor; MMP-9, metalloproteinase-9; 4-1BB, CD137 or TNF factor receptor superfamily T-cell costimulatory receptor; ICOS, CD278 or inducible T-cell costimulator; mDCs, myeloid dendritic cells; pDCs, plasmacytoid dendritic cells; MDSCs, myeloid-derived suppressor cells; NKT, natural killer T cells; ARG, arginine; iNOS, inducible nitric oxide synthase; PAX8, paired box gene 8 protein; CALB, calretinin; PDPN, podoplanin; TRAF6, TNF receptor–associated factor protein-6; FOXM1, Forkhead box protein M1; ADAMTS6, ADAM metallopeptidase With thrombospondin type 1 motif 6; FasL, Fas ligand; TRAIL, TNF-related apoptosis-inducing ligand; LBP, lipopolysaccharide binding protein; FGG, fibrinogen gamma chain; FGA, fibrinogen alpha chain; GSN, gelsolin; CAF1, caveolin-1; lncRNA, long non-coding RNA; MALAT1, metastasis−associated lung adenocarcinoma transcript 1; EXOSC4, exosome component 4; ALOX5AP, arachidonate 5-lipoxygenase–activating protein; ANXA1, annexin-A1; PLK3, Polo-like kinase-3; SREBF1, sterol regulatory element–binding transcription factor 1; LAG3, lymphocyte activation gene-3; IGFBP2, insulin-like growth factor binding protein 2; UQCRFS1, ubiquinol-cytochrome C reductase, Rieske iron-sulfur polypeptide 1; KRAS, Kirsten rat sarcoma virus; KLF4, Kruppel-like factor 4; HOXA5, homeobox protein Hox-A5; GMPR, guanosine 5′-monophosphate oxidoreductase; ISG20, interferon-stimulated gene 20-kDa protein; SNRPD1, small nuclear ribonucleoprotein D1 polypeptide.

## Obstacles in the treatment of the HGSOC

The treatment of ovarian cancer is based on debulking cytoreductive surgery, platinum-taxane–based first line chemotherapy, second-line chemotherapy, and targeted therapy approved by the FDA (Food and Drug Administration) and EMA (European Medicines Agency) using bevacizumab and poly-ADP-ribose polymerase (PARP) inhibitors. Several others drugs are being tested in clinical trials including programmed death-1 (PD-1)/ programmed death ligand-1 (PD-L1) inhibitors. HGSOC is initially a chemosensitive tumor, especially in the cases of positive *BRCA* germinal or somatic mutations. However, recurrent tumors are mostly chemoresistant due to activation of many mechanisms associated with the exceptional function and proliferative activity of OCSCs or reverse *BRCA* mutations occurring during the treatment. Moreover, the unique pattern of cancer spread inside peritoneal cavity that utilizes both collective invasion and sanguiferous route is relatively early phenomena in the course of the disease. The important obstacle in the effective treatment of HGSOC is also tumor heterogeneity comprehended as spatial heterogeneity in the different areas of the tumor, the inter-patient heterogeneity, and temporal heterogeneity between primary tumors, metastases, and recurrent disease. Even OCSCs themselves exhibit unexpected phenotypic plasticity and may differ in the same patient or among different patients depending on the cancer molecular type, advancement of the disease, patient health, and treatment scheme. The conclusion from those observations is that the use of the uniform treatment for all patients or for all temporal stages of the tumor is an oversimplification that results in observed unsatisfactory results in the context of both OS and PFS. The complexity of interaction between tumor cells, OCSCs, and TME in metastatic niche is another factor of great importance for supporting tumor growth, enhancing chemoresistance and the immune attack defiance. Therefore, tumor environment with all its components should also be treated as a target for anti-cancer therapy.

## Remarks on the targeting of the OCSCs

Taking the abovementioned reflections into consideration, the interesting targets for multidirectional treatment are OCSCs themselves and the components of OCSC microenvironment, particularly metastatic niche. One of the most explored areas of anti-OCSCs therapy is drugs directed against OCSC markers, signaling pathways, and epigenetic regulators. Targeting OCSC markers is important as chemotherapy, whereas decreasing tumor burden simultaneously increases the number of OCSCs. After exposition to chemotherapy, increased numbers of ascitic EpCAM+, CD44+, and OCT4+ cells were noted (248). Similarly, recurrent tumors contain more ALDHA1+, CD133+, and CD44+ OCSCs than primary tumors (49). These phenomena are observed not only in standard platinum/taxol-based chemotherapy but also in the tumors treated with PARP inhibitors (PARPis), where increased numbers of CD133+ and CD117+ OCSCs precede the acquired PARP resistance (249). However, targeting OCSC markers has to overcome two problems. The first one is that OCSC markers are not able to distinguish cancer stem cells exclusively, as about 75% of known cancer stem cell markers are also present on the surface of embryonic and adult stem cells (250). For instance, CD44 is present on hematopoietic cells, MSCs, and adipose-derived stem cells (251–253). CD117 is positive on 25% of embryonic stem cells (254), whereas CD166 is also found on epithelial cells, MSCs, and intestinal stem cells (255, 256). Intracellular cancer stem cell markers, like NANOG, OCT4, and SOX2, are also present in normal stem cells (257, 258). The second problem is associated with the fact that there is no universal cancer stem cell marker known. Tumor heterogeneity, differentiation status, and environment are reasons for OCSC different types. Therefore, the more effective strategy of elimination of OCSCs relies on targeting of at least two OCSC markers simultaneously. Targeting the signaling pathways used by OCSCs is also reasoned by the fact that many of them are likewise OCSC markers, activated after exposition to chemotherapy (259). Epigenetic regulation in ovarian cancer is associated with both hypermethylation and hypomethylation of DNA, as well as with histone methylation and acethylation. Hypermethylation of DNA contributes to formation of OCSCs (260). The CpG islands of many onco-suppressor genes were shown to be hypermethylated in ovarian cancer, leading to the loss of DNA-repair function and cell cycle control desynchronization (261). Upon chemotherapy, hypermethylation of genes responsible for cell resistance to apoptosis was detected (262). Gene hypomethylation is frequently observed in advanced HGSOC and correlates with worse survival (263). Histone methylation is engaged in upregulation of ATP-binding cassette drug membrane (ABC) transporters in chemoresistant OCSCs (264). Disturbed function of histone deacetylases promotes tumor progression (265). **Table 4** contains data on both the experimental and clinical trials of targeting OCSCs.

**Table 4:** Data on both the experimental and clinical trials devoted to targeting the OCSCs.

Target	Drug	Mechanism of action	Trial	Reference
**CD44**	ALM201	ALM201 is a residue peptide of FKBPL, which targets angiogenesis and CD44+ OCSCs by affecting the CD44/STAT3 pathway ALM201 is safe and well-tolerated, with stabilization of the disease in 22% of patients	Experimental Phase I dose-escalation human trial EudraCT	(266) (267)
**CD117**	Imatinib mesylate monotherapy Imatinib mesylate + docetaxel	Inhibition of CD117/PDGF signaling pathway. Treatment was well-tolerated, but no complete or partial responses were documented during a median follow-up of 6.6 months. However, 33% of patients had stable disease lasting from 4 to 8+ months. There was no relationship between best response (stable disease) and target expression Treatment showed low toxicity but also unsatisfactory anti-tumor activity. There were no objective responders. Median PFS was 2 months, and median OS was 10 months. Higher pre-treatment plasma concentrations of PDGF and VEGF were associated with shorter PFS and survival Patients with heavily pre-treated recurrent ovarian cancer expressing CD117 or PDGFRα showed ORR of 22%, which included one complete and four partial responses, and additional three patients had stable disease for more than 4 months	Phase II NCT00510653 Gynecologic Oncology Group study Southwest Oncology Group Protocol S0211 Hoosier Oncology Group trial	(268) (269) (270) (271)
**CD44/** **CD117**	Salinomycin monotherapy Salinomycin + paclitaxel Metformin + bevacizumab + cisplatin	Salinomycin is the mono carboxylic polyether antibiotic inhibiting ABC-transporter system and promoting OCSC apoptosis. Encapsulated salinomycin in the form of salinomycin-loaded high-density lipoprotein showed effective cellular uptake and reduced the EMT, stemness, and angiogenesis mediated by OCSCs Combined treatment reduced stemness and spheroid forming capability and enhanced apoptosis of ascitic OCSCs Combination of drugs reduced a significant number of CD44+CD117+ OCSCs and inhibited tumor growth	Experimental Experimental Experimental	(272) (273) (274)
**CD44/** **MyD88**	NV128	Isoflavone derivative causing depression of mitochondrial function and cellular starvation of OCSCs	Experimental	(275)
**ALDH**	673A CM37 ATRA + carboplatin 673A Disulfiram	The result of ALDH1A inhibition is an accumulation of toxic aldehyde metabolites in OCSCs. The effects are stronger in combination with ATR inhibitors ALDH1A inhibitor that disturbed spheroid production by the OCT4 and SOX2 downregulation A vitamin A derivative, in combination with carboplatin, suppresses ALDH1 expression and downregulates functionality of OCSCs The pan-ALDH1A inhibitor that preferentially kills CD133+ OCSCs through initiation of necroptosis and sensitizes tumor to platinum-based chemotherapy The anti-alcoholic medication, ALDH inhibitor, in combination with cisplatin, induced apoptosis and necrosis in ALDH+ cisplatin-resistant OCSCs	Experimental Experimental Experimental Experimental Experimental	(276) (277) (278) (279) (280)
	Selumetinib + Saracatinib PNA3 + guadecitabine	Both Src and MEK signaling kinases are co-activated in 31% of HGSOC. Combined treatment with Src inhibitor saracatinib and MEK inhibitor selumetinib decreased ALDH1+ cell sphere formation and loss of ALDH1+ OCSCs LncRNA HOTAIR is upregulated in HGSOC and especially in ALDH1+ OCSCs. Peptide nucleic acid PNA3 inhibits HOTAIR, and enhancer of zeste homolog 2 (EZH2) interaction and when combined with DNMT inhibitor guadecitabine abrogates ALDH1+ spheroid formation and decreases their number and tumor-promoting ability	Experimental Experimental	(281) (282)
**ALDH/** **CD133**	Salinomycin monotherapy Licofelone Metformin	Graphene oxide–silver nanocomposite combined with salinomycin showed high toxicity against ALDH+CD133+ OCSCs COX/5-LOX inhibitor that reversed stem-like properties in spheroids and augmented paclitaxel activity resulting in prolongation of mice survival Ovarian cancer II-IV FIGO. Metformin in combination with standard chemotherapy in neoadjuvant and adjuvant setting. Median PFS of 18 months, and median OS of 58 months. Tumors treated with metformin had a 2.4-fold decrease in ALDH+CD133+ CSCs and showed increased sensitivity to cisplatin	Experimental Experimental Phase II NCT01579812	(283) (284) (285)
**CD133**	Salinomycin monotherapy dCD133KDEL anti-CD133 CAR-NK cells + cisplatin	Conjugates of salinomycin with anti-CD133 antibody and nanoparticles are effective in transportation of the antibiotic into CD133+ OCSCs Deimmunized *Pseudomonas* endotoxin conjugated to anti-CD133 antibody inhibits tumor growth Sequential treatment using CAR-NK cells and cisplatin eradicated CD133+ OCSCs from cell lines and cell cultures obtained from ascites samples	Experimental Experimental Experimental	(286) (287) (288)
**EpCAM**	EpCAM-specific CAR-T cells Catumaxomab	Infusion of CAR-T cells delayed tumor progression in xenograft mice model of peritoneal carcinomatosis Hybrid moAb against EpCAM/CD3. Intraperitoneal use of this moAb resulted in prolongation of puncture-free interval (two-fold from 12 to 27.5 days) and time to first therapeutic puncture (four-fold from 12 to 52 days) in heavily pre-treated patients with EpCAM+ recurrent tumors complicated with malignant ascites. The median puncture-free survival and overall survival were 29.5 and 111 days, respectively Deterioration of quality of life appeared earlier in control than in catumaxomab-treated group of patients with ascites (19–26 days vs. 47–49 days) In patients with malignant ascites, peritoneal catumaxomab infusion enhanced the expression of the CD69 and CD38 activation molecules in T CD4+ and T CD8+, NK cells, and macrophages and enhanced T CD8+ accumulation into the peritoneal cavity	Experimental NCT00326885 Phase II European Medicines Agency approved Phase II/III NCT00836654 CASIMAS Phase IIIb NCT00822809	(289) (290) (291) (292)
**ATR**	Ceralasertib (AZD6738) + olaparib M6620 (VX-970) + carboplatin	ATR is a protein kinase involved in recognition of DNA damage and activation of DNA damage checkpoint. Inhibitors of ATR combined with PARP inhibitors (PARPi) were able to overcome PARPi and platinum resistance in *BRCA* and *CCNE1* wild and mutated cell lines Well-tolerated therapy with reduction in tumor burden, especially in BRCA-mutated patients (median PFS was 4.2 months overall and 8.2 months for patients with BRCA1 mutations) Partial response in platinum-resistant patients with *BRCA1* mutation. A patient with advanced germline BRCA1 ovarian cancer achieved RECIST partial response despite being platinum-refractory and PARP inhibitor–resistant	Experimental CAPRI phase II Phase I	(293) (294) (295)
**FAK**	Defactinib (VS-6063) + paclitaxel VS-4718 + platinum APG-2449	FAK is a tyrosine kinase activated by matrix and integrin receptors controlling cell motility. FAK inhibitor VS-6063 enhances chemosensitivity and decreases CD44 OCSC marker. Combination with paclitaxel reduces >90% tumor weight. Modest activity in advanced platinum-resistant ovarian cancer FAK inhibitor combined with platinum triggered ovarian cancer cell apoptosis and restored chemosensitivity A multikinase inhibitor of FAK, ROS proto-oncogene 1 receptor tyrosine kinase (ROS1), and anapestic lypmphoma kinase (ALK). Combination of APG-2449 and osimertinib (EGFR tyrosine kinase inhibitor) and mitogen-activated extracellular signal–regulated kinase inhibitor trametinib overcomes osimertinib resistance	Experimental NCT01778803 Phase I Experimental Experimental	(296) (297) (298) (299)
**Calcium channels**	Manidipine Lacipidine Benidipine Lomerizine Manidipine + paziotinib	Calcium channel blockers were found to target the OCSC function by decreasing steroid formation, proliferation, and induction of apoptosis. Use of these drugs downregulated expression of stemness markers OCT4, NANOG, SOX2, ALDH1, and CD133. Combination of calcium channel blocker with pan-HER inhibitor paziotinib showed synergism in reduction of OCSC spheroid formation, expression of stemness markers, and enhancement of apoptosis	Experimental Experimental	(300) (301)
**MSH-1/MSH-2**	siRNA	Dual knockdown of MSH-1 and MSH-2 downregulated OCSC ALDH4A1 and Myc and improved chemosensitivity	Experimental	(302)
**ERβ receptor**	LY5000307 (Erteberel) monotherapy	Selective agonist of estrogen receptor ERβ. Treatment with the agonist reduced the viability, sphere formation capacity, self-renewal, and invasion of OCSCs while augmenting their apoptosis	Experimental	(303)
**NAMPT**	FK866 + cisplatin	NAMPT is an enzyme for the NAD+ biosynthetic salvage pathway and is overexpressed in cancers. Combination of NAMPT inhibitor and cisplatin inhibited expression of ALDH1 and CD133 OCSCs and improved survival in the mouse model	Experimental	(304)
**Survivin**	AS602801 CEP-1347	Inhibitor of c-Jun N-terminal kinase downregulates survivin. Chemo-sensitization of OCSCs to carboplatin and paclitaxel A small-molecule kinase inhibitor downregulates survivin and sensitizes OCSCs to standard chemotherapy	Experimental Experimental	(305) (306)
**Wnt signaling pathway**	Ipafricept (OMP54F28) + carboplatin + docetaxel WNT974 + carboplatin Vantictumab (OMP-18R5)	Inhibitor of Fc-Frizzled-8 receptor antagonizing Wnt signaling. Sequential combined treatment is well-tolerated but has limited efficacy. The ORR was 76%. Median PFS was 10.3 months and OS was 33 months Inhibitor of PORCN that lowers secretion and binding of Wnt to its receptor. Combined therapy caused cell cycle arrest and cytotoxicity of cells isolated from ascites of patients with HGSOC moAB that inhibits Wnt pathway by targeting the Frizzled receptors on cancer cells. Treatment with vantictumab before paclitaxel therapy sensitizes cancer cells to death	NCT02050178 Phase I Experimental Experimental	(307) (308) (309)
**Hedgehog signaling pathway**	Cyclopamine Vismodegib (GDC-0449) monotherapy Sonidegib (LDE225) + paclitaxel	Steroidal alkaloid isolated from poisonous plant *Veratrum californicum* that inhibits Hedgehog signaling. Inhibition of spheroid-forming cells in the cell culture was observed upon treatment with cyclopamine The SMO receptor antagonist. Therapy was well-tolerated; however, the anticipated increase in PFS was not achieved. Median PFS was 7.5 months the in treated group and 5.8 months in the placebo group. Hedgehog expression was detected only in 13.5% of tissues The SMO receptor antagonist. Combination of drugs was well-tolerated and showed partial responses or stabilization of the disease in ovarian cancer	Experimental NCT00739661 phase II Phase I	(69) (310) (311)
**NOTCH signaling pathway**	LY900009 monotherapy MK-0752 + cisplatin RO4929097 monotherapy Enoticumab (REGN421) monotherapy Enoticumab + aflibercept Demcizumab (OMP-21M18) + paclitaxel Navicixizumab (OMP-305B83) monotherapy Navicixizumab (OMP-305B83) + paclitaxel	Inhibitor of γ-secretase protein. Therapy was well-tolerated, and five patients with solid tumors including ovarian cancer had stabilization of the disease Inhibitor of γ-secretase protein. Combination effectively stimulated cancer cells apoptosis and reduced growth of ovarian cancer xenografts in mice Inhibitor of γ-secretase protein. Monotherapy in recurrent ovarian cancer was well-tolerated but had insufficient activity. Fifteen of the 40 patients had stabilization of the disease lasting with median of 3 months. The results were better in HGSOC with high expression of intracellular NOTCH protein moAb against DLL4–NOTCH ligand involved in angiogenesis. Therapy had acceptable toxicity. In the group of patients with solid tumors including ovarian cancer, two partial responses and 15 stabilizations of the disease were observed Combination of anti-DLL4 and anti-VEGF therapy showed greater anti-tumor effects compared to either monotherapy in murine model of ovarian cancer moAb against DLL4. Combination showed ORR of 21% with manageable toxicity in the group of patients with highly pre-treated HGSOC with platinum-resistant tumors Combined dual moAb anti-DLL4/anti-VEGF. Showed acceptable toxicity profile and reduced the tumors in seven of the 11 of patients with pre-treated ovarian cancer Combination demonstrated manageable toxicity and ORR of 33% in bevacizumab pre-treated patients, 64% in bevacizumab naive patients, and 62% in the biomarker (high angiogenesis and suppressed immune response)–positive group	Phase I Experimental Princess Margaret, Chicago and California consortium Phase II Phase I Experimental SIERRA Phase Ib Phase Ia Phase Ib	(312) (313) (314) (315) (316) (317) (318) (319)
**PI3K/AKT/mTOR signaling pathway**	Metformin monotherapy Metformin + cisplatin/paclitaxel LY294002 + carboplatin Atorvastatin	Activation of AMPK followed by inhibition of signaling and reduction of energy consumption by OCSCs. Metformin inhibited cell viability, invasion, and autophagy while promoting apoptosis in paclitaxel-resistant ovarian cancer cell lines via downregulation of lncRNA SNHG—a regulator of PI3K/AKT/mTOR pathway Combination of Metformin with chemotherapy significantly reduced cell proliferation and migration and increased chemosensitivity by reducing the OCSCs in treated cell lines Addition of metformin to standard adjuvant or neo-adjuvant chemotherapy reduced two-fold concentration of ALDH+CD133+ OCSCs and increased cisplatin sensitivity of tumors, resulting in median OS of 58% PI3K antagonist combined with carboplatin enhances its anti-cancer effect in mouse xenograft model Statin that, through inhibition of AKT/mTOR pathway, stimulates apoptosis of ovarian cancer cells and inhibits cell invasion	Experimental Experimental NCT01579812 Phase II Experimental Experimental	(320) (321) (285) (322) (323)
**NF-**κ**B signaling pathway**	Metformin + cisplatin/paclitaxel MK-5108 monotherapy	Metformin through inhibition of NF-κB signaling pathway enhanced sensitivity to standard chemotherapeutics in both sensitive and resistant cell lines Aurora-A kinase inhibitor. Its use in ovarian cancer cell lines caused cell cycle arrest, inhibition of NF-κB signaling, and cytokine secretion	Experimental Experimental	(324) (325)
**Hippo/YAP signaling**	Verteporfin Verteporfin + carboplatin/taxol	Photosensitizer releases a singlet oxygen and ROS toxic to cancer cells upon exposure to light of particular wavelength. Verteporfin-loaded lipid nanoparticles inhibited tumor xenografts in mice upon laser light exposure Combination was efficient in reducing proliferation, invasion, and clonogenic capacity of ovarian cancer cell lines	Experimental Experimental	(326) (327)
**JAK/STAT signaling**	Ruxolitinib + paclitaxel TG101209 CYT387 + paclitaxel JQ1	Inhibitor of JAK, thus inhibiting the JAK/STAT pathway. Synergic effects of combined therapy on tumor growth in mouse model of advanced/ascites+ ovarian cancer JAK2 inhibitor that induced cytotoxicity in spire-forming CD24+ cells, thus inhibiting migration and metastasis of ovarian cancer in murine model Combination of JAK2 inhibitor with chemotherapy inhibited paclitaxel-mediated OCSC enrichment and reduced tumor burden in mouse xenografts Selective small-molecule bromodomain inhibitor that inhibits JAK/STAT pathway. JQ1 resensitized ovarian cancer cells to platinum	Experimental Experimental Experimental Experimental	(328) (329) (38) (330)
**TGF-β signaling**	SB525334	TGF-β1 receptor inhibitor blocked ALDH1+ OCSCs self-renewal, invasion, and spheroid formation	Experimental	(331)
**Src and MAPK signaling kinases**	Selumetinib + Saracatinib	MAPK and Src inhibitors showed synergistic induction of apoptosis and tumor inhibition in ovarian cancer mouse model. Treatment decreased spheroid formation and ALDH1 expression	Experimental	(281)
**DNA methylation**	Decitabine + carboplatin	Decitabine is a DNMT1 inhibitor, a hypomethylating agent. In the group of pre-treated patients with solid tumors, containing two ovarian HGSOC tumors, a partial response to combined therapy was observed	NCT01799083 Phase II	(332)
	Decitabine + carboplatin/paclitaxel + cytokine-induced killer cells (CIK) Guadecitabine + carboplatin Guadecitabine + PNA3 Azacitidine + carboplatin	The population of patients with chemoresistant recurrent ovarian cancer treated with combined regimen showed ORR of 87.5% and prolongation of PFS to 8 months and OS to 19 months This regimen combining DNMT1 inhibitor with chemotherapy, compared to second-line chemotherapy alone, resulted in increased rate of patients having 6-months PFS (37% vs. 11%) PNA3 is HOTAIR inhibitor. Combined with DNMT1 inhibitor showed reduction of spheroids and ALDH1+ OCSCs Combination of DNMT1 inhibitor with carboplatin caused stabilization of the disease > 4 months in three patients with refractory or resistant ovarian cancer Sequential combined treatment significantly slowed platinum-resistant HGSOC growth and activated immune-related pathways priming tumor for checkpoint inhibitor immunotherapy	Phase II Phase II Experimental Phase II Experimental	(333) (334) (282) (335) (336)
**Histone deacetylation**	Vorinostat monotherapy Vorinostat + carboplatin + gemcytabine Belinostat (PXD-101) + carboplatin + paclitaxel Belinostat (PXD-101) + carboplatin Entinostat (MS-275) monotherapy Entinostat (MS-275) + olaparib	HDAC inhibitor that induces accumulation of acetylated histones and transcription factors that arrest cell cycle. Monotherapy of platinum-resistant progressive HGSOC was well-tolerated; however, clinical efficacy was minimal (two women had PFS over 6 months, with one having a partial response) Combination was effective (partial response in six of the 15 patients) in recurrent platinum-sensitive ovarian cancer but was accompanied with hematological toxicity HDAC inhibitor that induces apoptosis and sensitizes tumor for chemotherapy. Combination had acceptable toxicity; in three of the 35 patients, complete response was obtained, and, in 12 of the 35 patients, partial response was obtained. ORR was 44% in platinum-resistant and 63% in platinum-sensitive patients. Combination was effective in 14 of the 27 patients with platinum-resistant ovarian cancer (one complete and one partial response; 12 patients had disease stabilization) Benzamide derivative of HDAC that inhibits selectively class I and IV HDAC. Entinostat was effective in therapy of intraperitoneal tumors in mouse model; however, its activity depended on immunocompetence represented by upregulation of MHCII and infiltration of T CD8+ cytotoxic cells in the tumors Combination potentiates the effect of olaparib in HR-proficient ovarian cancer and enhances olaparib-induced DNA damage	Gynecologic Oncology Group Phase II Phase I Phase II Gynecologic Oncology Group Phase II Experimental Experimental	(337) (338) (339) (340) (341) (342)
**Metabolism**	2-deoxy-D-glucose (2DG) Devimistat (CPI-613) TVB-3166 *USP13* knockdown Etomoxir Perhexiline CAY-10566	Glycolysis inhibitor. In HGSOC tumors with reduced beta-F1-ATPase/oxidative phosphorylation, it sensitized cancer cells for platinum Mitochondrial metabolism inhibitor. Preferentially targets OCSCs and prevents acquired chemoresistance into olaparib or carboplatin/paclitaxel therapy Fatty acid synthase (FASN) inhibitor. Induced apoptosis in HGSOC model Inhibits ATP citrate synthase (ACLY) followed by inhibition of ovarian HGSOC tumors in the mouse xenograft model An irreversible inhibitor of carnitine palmitoyltransferase-1 (CPT-1) in mitochondria. Targets OCSCs and induces their apoptosis, and inhibits growth of tumor xenografts Inhibitor of carnitine palmitoyltransferase-1 (CPT-1) in mitochondria. Sensitized *NKX2-8*-deleted HGSOC lines to cisplatin Selective inhibitor of stearoyl-CoA-desaturase-1 (SCD1). Induced apoptosis and ferroptosis in HGSOC lines and eliminated OCSCs and spheroid formation	Experimental Experimental Experimental Experimental Experimental Experimental Experimental	(343) (344) (345) (346) (347) (348) (162, 349)

FKBPL, FK506-binding protein–like; PDGF, platelet-derived growth factor; PDGFR, platelet-derived growth factor receptor; ALDH, aldehyde dehydrogenase; ATRA, all-trans retinoid acid; COX, cyclooxygenase; 5-LOX, Arachidonate 5-lipooxygenase; NK, natural killer cells; CAR, chimeric antigen receptor; EpCAM, epithelial cell adhesion molecule; ATR, ataxia telangiectasia and Rad3-related protein; OCT4, Octamer-binding transcription factor 4; SOX2, sex determining region Y-box 2; NANOG, homeobox protein NANOG; FAK, focal adhesion kinase; CCNE1, cyclin E1; MSH, Musashi protein; siRNA, small interfering RNA; NAMPT, nicotinamide phosphoribosyltransferase; PORCN, porcupine acetyltransferase; SMO, smoothened receptor; moAb, monoclonal antibody; DLL4, delta-like ligand-4; VEGF, vascular endothelial growth factor; ORR, overall response rate; AMPK, AMP-activated protein kinase; lncRNA, long non-coding RNA; SNHG, small nucleolar RNA host gene 1; OS, overall survival; Src, Src non-receptor tyrosine kinase; MAPK, mitogen-activated protein kinase; JAK, Janus kinase; STAT, signal transducer and activator of transcription protein; DNMT1, DNA-(cytosine-5)-methyltransferase-1; ORR, overall response rate; PNA, peptide nucleic acid; HOTAIR, lac RNA HOX antisense intergenic RNA; PFS, progression-free survival; HDAC, histone deacetylase; MHC, major histocompatibility complex; HR, homologous recombination; USP13, ubiquitin specific peptidase 13; NKX2-8, NK2 Homeobox 8 protein.

## Remarks on the targeting of the tumor microenvironment

One of the most important targets in TME is CAFs. However, the past experience with anti-CAFs therapy has indicated that the aim in this approach should be to revert CAFs functionally back to normal fibroblasts, rather than eradicating them completely from the TME. Eradication of CAFs has proved to change the tumor into more aggressive phenotype, instead of eliminating tumor cells (350). It is even more important taking into consideration that CAF populations of different tumor-promoting abilities and phenotype (CD49e+, fibroblast activation protein FAP-high or FAP-low) have been identified (351). The reprogramming of M2 tumor-associated macrophages (TAMs), another key population of tumor-supporting cells, into M1 phenotype could be similarly to CAFs, which is a better option than eliminating them completely (352). Another, recently identified population of cells in TME is cancer-associated mesothelial cells (CAMs) that originate from peritoneal normal mesothelial cells activated by cancer-derived promoting factors that induce mesothelial–mesenchymal transition and secretion of factors, enhancing peritoneal metastases and chemoresistance (353). Hepatocyte growth factor (HGF) released from ovarian cancer cells in hypoxic conditions induces the senescence of mesothelial cells and downregulates the expression of junctional proteins that results in disintegration of mesothelial integrity and enables cancer invasion through the mesothelial barrier (354, 355). Phenotypic changes of mesothelial cells to CAMs are mediated by TGF-β and CD44 and annexin A2 secreted inside exosomes from cancer cells (356–358). In response to those changes, CAMs secrete VEGF and upregulate fibronectin expression in ECM, thus promoting tumor vascularization and binding of tumor cells’ integrins to ECM to support metastases (108, 359). Moreover, CAMs increase secretion of IL-8 and CCL2 that stimulate pyruvate dehydrogenase kinase-1 in cancer cells followed by increased expression of integrins to enhance adhesion and migration (360, 361). Interaction between intelectin-1 on CAMs and lipoprotein receptor–related protein-1 on cancer cells also contributes to invasion by upregulation of MMP-1 (362). CAMs pre-stimulated by cancer cell–derived TGF-β secret osteopontin, which, in turn, activates CD44/PI3K/AKT pathway in OCSCs, leading to ABC transporters’ overexpression and chemoresistance (363). M2-shifted TAMs also support CAMs activity by macrophage inflammatory protein-1β that activates P-selectin secretion by CAMs, followed by stimulation of CD24 on the cancer cells’ surface and increased adhesion (364). CAMs are, in turn, able to polarize the TAM phenotype into M2 type (365). CAMs are also capable to regulate the expression of glucose transporter type 4, resulting in increased glucose intake by cancer cells and growth promotion (362). Because of all above functions, CAMs are an interesting target for anti-TME therapy in ovarian cancer.

The next promising target for the therapy is metabolism of cancer cells. Cancer cells use both aerobic glycolysis (the Warburg effect) and oxidative phosphorylation (OXPHOS). Aerobic glycolysis protects cells from oxydative stress and fuels proliferation. However, OXPHOS and resistance to glucose deprivation in tumor environment are a metabolic adaptation enabling chemoresistance. Both ways of glucose metabolism are therefore used by cancer cells, including OCSCs and are another sign of their plasticity (366–368). The metabolic interactions between omental adipocytes and OCSCs are another reason for cancer progression and chemoresistance. Fatty acids could be very efficient source of energy that fuels the spread and growth of peritoneal implants (369). Adipocytes are stimulated by cancer cells to release fatty acids into metastatic niche, and, in turn, adipocytes induce expression of fatty acid receptor CD36 on cancer cells, thus enhancing uptake of fatty acids by cancer (370). Colonization of omental tissue depends on expression of salt-inducible kinase 2 (SIK2) in cancer cells. SIK2 kinase stimulates cell proliferation in PI3K/AKT-mediated manner and enhances paclitaxel resistance in HGSOC cells (371). Moreover, fatty acid oxidase and fatty acid synthase (FASN) have been shown to sustain survival of cancer cells in TME and increase resistance to anoikis and chemotherapy and spheroid formation in HGSOC lines (347, 372). Ovarian cancer CSCs indicate increased concentration of unsaturated lipids and what enhances cell membrane fluidity and facilitates OCSC plasticity and self-renewal. Inhibition of desaturases inhibits spheroid formation and abrogates tumor growth and metastases (373).

Another potential target for anti-TME therapy in HGSOC is exosomes. The identification of their origin inside TME and the recognition of their cargo have the key role in exosome-directed therapy. Exosomes could be also used as potential vehicles for the transportation of drugs into the tumor. It was also found that exosomes secreted from untreated tumors have a significant influence on the expression of many genes involved in functional change of fibroblasts into CAFs and in stimulation of tumor metastases. Such ability was less evident in exosomes secreted by pre-treated tumors (374). The situation is, therefore, complicated, as it seems that exosomes differ depending not only on the type of secreting cell but also on its functional status and temporal changes during therapy. Exosomes are able to influence several mechanisms of tumor growth. Their cargo, including proteins, neoantigens, cytokines, growth factors, and miRNAs, is responsible for cancer progression, metastases, and chemoresistance. Exosomes contain also modulators of immune response capable of inhibition of macrophages: natural killer (NK) cells, dendritic cells (DCs), and B and T lymphocytes (375, 376). Exosomes negatively regulate immunosurveillance of the host against tumor, through inhibition of T lymphocytes, NK cells, DCs, and monocytes in tumor environment and ascites (227, 377–379). Exosomes stimulate tumor angiogenesis affecting the VEGF and HIF-1α expression and by activation of Wnt/β-catenin and NF-κB signaling pathways (380, 381). Exosomes influence also stroma remodeling by cooperation with CAFs and adipocyte-derived stem cells (165). Recently, tumor-derived exosomal miR-141 was identified as a regulator of stromal-tumor interactions and inducer of tumor-promoting stromal niche by activation of YAP/chemokine (C-X-C motif) ligand 1 (GROα)/CXCR signaling pathway (382). One of the most interesting vectors of information between cancer cells and TME is non-coding miRNAs and long non-coding RNAs (lncRNAs). They were found in the serum and ascites of patients with ovarian cancer (227, 228); however, their presence in tumor-derived exosomes ensures safe and undisturbed transportation to the target cells. Non-coding RNAs play extremely important functions. Exosomes loaded with miR-1246 are able to enhance pro-tumorigenic effects of M2-shifted TAMs and to facilitate paclitaxel resistance (383). Cancer cell–derived miR-21-3p, miR-222, miR-125b-5p, miR-181d-5p, and miR-940 target TAMs and polarize them into M2 phenotype (172, 384). miR-99a-5p affects human peritoneal mesothelial cells and enhances cancer cell invasion (385). The Let-7a and miR-200a regulate tumor invasiveness (386). Exosomes containing lncRNAs ENST00000444164 and ENST0000043768 are responsible for activation of NF-κB signaling in cancer cells (387). **Table 5** presents data on targeting the components of TME and OCSC niche.

**Table 5:** Data on the experimental and clinical trials of drugs targeting the tumor microenvironment and OCSC metastatic niche.

Target	Drug	Mechanism of action	Trial	Reference
**MSCs**	Metformin Letrozole + Ruxolitinib	Prevention of reprogramming of MSCs into active cancer-associated cells and decrease of tumor Tregs. Clinical utility in early ovarian cancer and pre-treatment in immunotherapy regimens Combined therapy of anti-estrogen drug with inhibitor of JAK enables inhibition of LIF/IL-6–mediated signals from cancer-associated MSCs, which is followed by sensitization of ovarian cancer to anti-estrogen therapy	Experimental Experimental	(388) (389)
**MDSCs**	Alemtuzumab	moAb targeting CD52 expressed by vascular leukocytes and Tie2+ monocytes, thus disturbing interactions with SIGLEC10. This moAb induces complement-dependent lysis of CD52-positive cells in ascites and restricts angiogenesis	Experimental	(390)
**CAMs**	Rilotumumab (AMG102) Oregovomab monotherapy Oregovomab + carboplatin + paclitaxel	Anti-HGF moAb. Although well-tolerated, the treatment showed very limited efficacy Anti-MUC16 moAb. While used in monotherapy, oregovomab did not show any benefit to patients with persistent advanced ovarian cancer moAb combined with first line standard chemotherapy in patients with advanced ovarian cancer showed prolongation of both PFS and OS In patients with optimally resected advanced ovarian cancer, combination of drugs resulted in more than three times longer PFS compared to chemotherapy alone	Phase II Phase II Phase II Phase II	(391) (392) (393) (394)
	REGN4018 monotherapy Anetumab ravtansine + pegylated liposomal doxorubicin	Bispecific anti-MUC16/CD3 moAb inhibited the growth of murine peritoneal tumors Conjugate of anti-MUC16 moAb with cytotoxic maytansinoid tubulin inhibitor DM4. In recurrent ovarian cancer, this combination showed 28% response rate, mostly in the form of partial response	Experimental Phase Ib	(395) (396)
	Intetumumab (CNTO-95) monotherapy Volociximab monotherapy	moAb targeting αγβ3 and αγβ5 integrins. In patients with solid tumors including ovarian carcinosarcoma, it showed stabilization of the disease moAb against α5β1 integrin. In advanced platinum-resistant ovarian cancer, it failed to demonstrate efficacy	Phase I Phase II	(397) (398)
**TAMs**	Anti-CD24 moAb Hu5F9-G4 moAb Celecoxib + cyclophosphamide Celecoxib + carboplatin Celecoxib + carboplatin + docetaxel G5-MTX	CD24 is a “do not eat me” signal for macrophages. Disrupting the signal between CD24 on cancer cells and SIGLEC-10 on TAMs enhanced phagocytosis of cancer cells and could be a novel target for therapy moAb blocking phagocytosis mediated by CD47. Partial remission in two patients COX-2 inhibitor that blocks M2 shift of TAMs mediated by OCSC-derived COX-2 activity. Combination therapy did not show therapeutic benefit in recurrent ovarian cancer The response rate to this combination in recurrent platinum-resistant ovarian cancer was 29% In the group of Ic-IV stage ovarian cancer, addition of celecoxib to first-line standard chemotherapy did not show any benefit G5-methotrexate nanoparticles depleted TAMs in models of ovarian cancer and ascites and were able to reverse anti-angiogenic therapy resistance	Experimental NCT02216409 Phase I Phase II Phase II DoCaCel study Phase II Experimental	(399) (400) (401) (402) (403) (404)
	Clodronate liposomes (biphosphonate) Trabectedin Trabectedin monotherapy Trabectedin + pegylated liposomal doxorubicin Trabectedin + durvalumab Trabectedin + bevacizumab	Reduced metastases and ascites formation in HGSOC xenografts Extract from the sea squirt *Ecteinascidia turbinata* selectively cytotoxic to macrophages and reducing angiogenesis in mouse model of ovarian cancer Sensitizes platinum-resistant tumors for the consecutive platinum treatment Combination showed ORR of 28% in advanced recurrent ovarian cancer Patients with germline BRCA1/2 mutations and platinum-free interval of 6–12 months had survival benefit from this treatment Combination with PD-1/PD-L1 inhibitor resulted in tumor shrinkage and 6-month PFS in 43% of patients with advanced ovarian cancer Combination showed benefit; 75% of patients had PFS of 6 months	Experimental Experimental Phase II Phase I Phase III TRAMUNE Study Phase Ib Phase II	(405) (406) (407) (408) (409) (410) (411)
	GW2580 ARRY-382 + pembrolizumab AC708 + anti-VEGF + paclitaxel Mannose receptor (CD206)-targeted nanocarrier of IRF5 and IKKβ mRNA	CSF1R inhibitor inhibits CSF1/CSF1R pathway responsible for TAMs survival. Reduction of ascites in the mouse model of ovarian cancer Combination with PD-1/PD-L1 inhibitor. One patient with ovarian cancer had partial response Partial restoration of sensitivity to anti-VEGF therapy in mouse xenograft model Reversing TAM phenotype into M1. Reduces tumor growth in mouse model of ovarian cancer	Experimental NCT02880371 Phase Ib/II Experimental Experimental	(412) (413) (414) (415)
**CAAs**	Metformin	Inhibits adipocyte-mediated cancer cell proliferation, migration, and bio-energetic changes	Experimental	(416)
**CAFs**	A-83-01 LY2109761 + cisplatin Sorafenib monotherapy Sorafenib + bevacizumab	CAF-derived TGF-β promotes tumor supporting environment. Inhibitor of TGF-β signaling pathway decreased peritoneal metastases and improved survival in mouse model of ovarian cancer Combination of TGF-β type I/type II inhibitor with cisplatin increased significantly cytotoxic activity of chemotherapeutic in chemoresistant cell line In recurrent ovarian cancer and peritoneal carcinomatosis, therapy was effective in two of the 59 patients with partial response and 20 of the 59 patients with disease stabilization but showed substantial toxicity CAFs secrete growth factors stimulating cancer proliferation. Combination of multi-kinase inhibitor against PDGFR, VEGFR, Raf, and CD117 with anti-angiogenic therapy resulted in partial response in 9 of the 35 patients and stabilization in 18 of the 35 of patients	Experimental Experimental Gynecologic Oncology Group Phase II Phase II	(417) (418) (419) (420)
	Sorafenib + topotecan Sorafenib + gemcitabine Sorafenib + paclitaxel + carboplatin	In recurrent ovarian cancer, therapy was effective in five of the 30 patients with partial response, and 14 of the 30 patients with disease stabilization but showed substantial toxicity Oral sorafenib in combination with topotecan and continued as maintenance monotherapy showed significant prolongation of PFS compared to placebo in recurrent ovarian cancer, with acceptable toxicity The combination was associated with encouraging rates of stable disease and CA-125 response, with manageable toxicity The combination of sorafenib and standard therapy in the first-line treatment of advanced ovarian cancer did not improve efficacy and substantially increased toxicity	Hoosier Oncology Group Phase II NCT01047891 Phase II Princess Margaret Hospital Phase II Sarah Cannon Research Institute Phase II	(421) (422) (423) (402, 424)
	Aflibercept monotherapy Cediranib (AZD2171) monotherapy	This recombinant fusion VEGFR1/2 protein extracellular domain is a decoy receptor that inhibits pro-angiogenic signaling from CAFs. Aflibercept was effective in controlling of malignant ascites in advanced ovarian cancer Receptor tyrosine kinase inhibitor that inhibits VEGFR1/2/3 and PDGFR-α/-β and CD117, thus blocking signals from CAFs; 17% of patients and 13% of patients had partial response and stable disease, respectively In platinum-sensitive group, there was partial response in 26% and stable disease in 51% of patients. In the group of platinum-resistant patients, only stabilization of the disease in 66% was observed	Phase II Phase II Princess Margaret, Chicago and California consortium Phase II	(425) (426) (427)
	Cediranib + olaparib	Combination of two drugs used in the group of patients with progressive HGSOC pre-treated with platinum (both resistant and sensitive) and with acquired PARP inhibitors resistance. Sixteen-week PFS varied from 39% to 55% and was dependent on genomic alterations, being the shortest in the group with reverse *BRCA1*, *BRCA2*, and *RAD51B* mutations and *ABCB1* upregulation In platinum-sensitive recurrent ovarian cancer, combination of drugs did not improve PFS, compared to platinum-based chemotherapy. However, in patients with germinal *BRCA* mutation, it was significantly effective In patients with platinum-resistant recurrent *BRCA* germline mutation-negative ovarian cancer pre-treated with a median of 4 lines of chemotherapy and bevacizumab, the ORR with this drug combination was 15%, PFS was 5 months, and OS was 13 months	EVOLVE Phase II NRG-GY004 Phase III CONCERTO Phase III	(428) (429) (430)
	Nintedanib (BIBF1120) monotherapy Nintedanib (BIBF1120) + carboplatin + paclitaxel Pazopanib (GW786034) + paclitaxel	Receptor tyrosine kinase inhibitor that inhibits VEGFR1/2/3 and FGFR1/2/3 and PDGFR-α/-β. Maintenance therapy after completed chemotherapy for relapsed ovarian cancer showed improvement of PFS (16% vs. 5%) Combination of drugs compared to standard chemotherapy alone showed modest efficacy (improved PFS) in the group of patients with advanced HGSOC and upfront debulking surgery Receptor tyrosine kinase inhibitor that inhibits VEGFR1/2/3, PDGFR-α/-β, and CD117. Combination of drugs compared to paclitaxel alone showed improvement in PFS (6.3 vs. 3.5 months) and OS (18.7 vs. 14.8 months) in advanced platinum-resistant or platinum-refractory ovarian cancer	Phase II AGO-OVAR12 Phase III MITO-11 Phase II	(431) (432) (433)
	Pazopanib (GW786034) + paclitax el Pazopanib (GW786034) + cyclophosphamide Pazopanib (GW786034) + chemotherapy Calcitriol Losartan + platinum + taxol	In ovarian cancer relapse during maintenance therapy with bevacizumab, combination of drugs compared to paclitaxel alone did not improve efficacy but increased toxicity In the group of recurrent, platinum-resistant and pre-treated ovarian cancer combination of drugs showed promising results (PFS of 8 months and OS of 25 months) Meta-analysis of five studies indicated that pazopanib combined with chemotherapy improved ORR, but without improvement in OS and with increase of toxicity Inhibits SMAD signaling in CAFs. Use of calcitriol improved survival of mice with xenografted HGSOC tumors Angiotensin inhibitor that showed activity against ovarian cancer in combination with standard therapy—prolongation of OS and reduction of ascites	TAPAZ Phase II PACOVAR Phase I Experimental Observational	(434) (435) (436) (437) (438)
**Immune cells**	Atezolizumab+ platinum + bevacizumab Atezolizumab + bevacizumab Avelumab + carboplatin + paclitaxel	Immune checkpoint inhibitor anti–PD-L1 moAb in combination with platinum-based chemotherapy and anti-angiogenic therapy in patients with newly diagnosed advanced ovarian cancer with residual disease after primary cytoreduction or in neo-adjuvant setting. No benefit was observed in Atezolizumab therapy compared to placebo In patients with platinum-resistant ovarian cancer, the partial responses were observed in three of 20 and stabilization of the disease in 8 of the 20 patients, respectively Immune checkpoint inhibitor anti–PD-L1 moAb addition to standard chemotherapy in advanced ovarian cancer after primary cytoreduction or in neo-adjuvant setting did not improve PFS	IMagyn050/GOG3015/ENGOT-OV39 Phase III Phase Ib JAVELIN Ovarian 100 Phase III	(439) (440) (441)
	Avelumab + pegylated liposomal doxorubicin Avelumab + Talazoparib Durvalumab + TPIV200 Durvalumab + Olaparib	In the group of patients with recurrent ovarian cancer pre-treated with at least three cycles for platinum-sensitive disease, combination did not improved either PFS or OS significantly Combination of PD-L1 and PARP inhibitor in solid tumors with *BRCA1/2* or *ATM* mutation (including ovarian cancer) did not reach the presumed PFS Combination of this PD-L1 inhibitor with folate receptor-α vaccine in recurrent advanced platinum-resistant ovarian cancer showed robust immune response but low response rate, however with unexpected prolongation of survival (median OS 21 months) In the group of patients with ovarian cancer with recurrent tumor, this combination showed overall disease control rate of 71% (partial response + stabilization), as well as switch into immunoreactive environment	JAVELIN Ovarian 200 Phase III JAVELIN BRCA/ATM Phase IIb Phase II Phase II	(442) (443) (444) (445)
	Durvalumab + Olaparib or Durvalumab + pegylated liposomal doxorubicin/topotecan/paclitaxel or Durvalumab + Tremelimumab (anti-CTLA-4 moAb) + pegylated liposomal doxorubicin/topotecan/paclitaxel Durvalumab + Olaparib + Cediranib Nivolumab + Ipilimumab Nivolumab + Bevacizumab	Combinations were adjusted to the biosignature of the tumors in the context of PD-L1 and homologous recombination deficiency (HRD) status. In the group of recurrent platinum-resistant ovarian cancer, this therapy showed median ORR of 37% with manageable toxicity Combination of PD-L1 and PARP inhibitors with VEGFR1-3 inhibitor showed disease control rate of 67% in the group of heavily pre-treated patients with gynecologic cancers including seven of the nine ovarian cancers Combined anti–PD-1 and CTLA-4 therapy compared to nivolumab monotherapy indicated better 6-month ORR (31% vs. 12%) and PFS (4 vs. 2 months) in recurrent/persistent ovarian cancer Combined therapy in relapsed ovarian cancer showed ORR of 40% in platinum-sensitive and ORR of 17% in platinum-resistant patients	AMBITION/KGOG3045 Phase III Phase I NRG Oncology Study Phase II Phase II	(446) (447) (448) (449)
	Nivolumab monotherapy Nivolumab + Galinpepimut-S Pembrolizumab + Bevacizumab + cyclophosphamid Pembrolizumab monotherapy	Monotherapy did not improve OS and PFS in patients with recurrent platinum-resistant ovarian cancer, when compared to gemcytabine or pegylated liposomal doxorubicin Combination with tetravalent Wilms’ Tumor 1 (WT1) peptide vaccine in patients with ovarian cancer with second/third remission and tumors showing WT1 expression indicated prolongation of PFS to 1 year in 70% of patients treated with more than two cycles This combination gave clinical benefit in 95% and median PFS of 10 months in the group of patients with mostly platinum-resistant recurrent ovarian cancer In the group of pre-treated with standard chemotherapy patients, monotherapy showed modest clinical efficacy (ORR not exceeding 10%) and better in tumor’s PD-L1 higher positivity	NINJA Phase III Phase I Phase II KEYNOTE-100 Phase II	(450) (451) (452) (453)
	Pembrolizumab + cisplatin + gemcytabine Pembrolizumab + Niraparib Pembrolizumab + low-dose carboplatin Pembrolizumab + pegylated liposomal doxorubicin	In recurrent platinum-resistant ovarian cancer, addition of pembrolizumab to chemotherapy did not result in a benefit of the therapy In recurrent platinum-resistant ovarian cancer, this combination showed disease control rate of 65% In recurrent platinum-resistant ovarian cancer, this combination showed ORR of 62% and better OS in the group of patients with the higher CD8+PD-1+Ki67+ T cells to the tumor burden ratio Combination showed ORR of 26% in the group of patients with recurrent ovarian cancer, and results were better compared to monotherapy using each drug separately	Phase II Phase II Phase II Phase II	(454) (455) (456) (457)
**Exosomes**	Peptide-engineered exosomes Tumor suppressor miRNA Exosomes with Triptolide Exosome-liposome hybrid nanoparticle delivery system α-Mangostin + cisplatin	Artificially generated exosomes with overexpression of miR-92b-3p could be used as an anti-angiogenic therapy MiR-199a-3p loaded to exosomes inhibited ovarian cancer proliferation and invasion and, in xenograft mouse model, inhibited peritoneal dissemination of cancer Diterpenoid epoxide packed into exosomes showed anti-proliferative effect on ovarian cancer cell lines and xenografted tumors, however with considerable hepatic and splenic toxicity Hybrid transport system packed with Triptolide and miR-497 showed apoptotic effects and enabled to overcome platinum resistance Combination of natural plant derivative with cisplatin changed the number and activity of CAF-derived exosomes	Experimental Experimental Experimental Experimental Experimental	(458) (459) (460) (461) (374)
**RNAs-targeted therapy**	Circ_EXOC6B RNA miR-671-5p Icariside II Circ_TYMP1 RNA circ_0026123 RNA	Circ_EXOC6B RNA suppressed the progression and paclitaxel resistance of ovarian cancer cells through sequestering miR-376c-3p miR-671-5p reduces tumorigenicity of ovarian cancer through suppressing histone deacetylase 5 (HDAC5) and HIF-1α expression Herbal component from *Epimedium brevicornum*. Suppresses the tumorigenesis of ovarian cancer by promoting autophagy by miR-144-3p/IGF2R axis Downregulation of circ_TYMP1 decreased ovarian cell proliferation and invasion by miR-182A-3p/TGF-β pathway Downregulation of this circRNA inhibited proliferation and metastases of ovarian cancer through miR-124-3p/enhancer of zeste homolog 2 (EZH2) pathway	Experimental Experimental Experimental Experimental Experimental	(462) (463) (464) (465) (466)
	miR-146a Follistatin mRNA anti-*PLXDC1* siRNA miR-20c	MiR-146a secreted by MSCs increased the chemosensitivity of ovarian cancer resistant cell lines to docetaxel Lipid nanoparticles containing mRNA for follistatin injected intraperitonealy inhibited cancer dissemination and prevented the onset of cachexia in the mouse model of ovarian cancer Chitosan nanoparticles containing anti-*PLXDC1* siRNA decreased tumor proliferation and microvessel density and increased apoptosis in murine model of ovarian cancer MiR-200c was used as an inhibitor of NRP1 transmembrane receptor highly expressed by ovarian cancer and connected to multi-drug resistance. Combination of miRNA with olaparib enhanced its cytotoxicity	Experimental Experimental Experimental Experimental	(220) (467) (468) (469)

MSCs, mesenchymal stem cells; MDSCs, myeloid-derived stem cells; HGF, hepatocyte growth factor; MUC16, mesothelin; CD52, CAMPATH-1 antigen; SIGLEC-10, silica acid binding Ig–like lectin-10; Tregs, T regulatory cells; CAAs, cancer-associated adipocytes; DNMT1, DNA-(cytosine-5)-methyltransferase-1; TAMs, tumor-associated macrophages; COX-2, cyclooxygenase-2; ORR, overall response rate; CSF1, colony-stimulating factor-1; CSF1R, colony-stimulating factor-1 receptor; IFR5, Interferon regulatory factor; IKKβ, IkappaB kinase beta; PDGFR, platelet-derived growth factor receptor; VEGFR, vascular endothelial growth factor receptor; Raf, RAF proto-oncogene serine/threonine-protein kinase; PFS, progression-free survival; BRCA, breast cancer type 1 susceptibility protein; RAD51B, RAD51 paralog B; ABCB1, ATP-binding cassette sub-family B member 1; FGFR, fibroblast growth factor receptor; ORR, overall response rate; PARP, poly-(ADP-rybose) polymerase; CTLA-4, cytotoxic T-cell antigen 4; IGF2R, insulin-like growth factor-2 receptor; mRNA, messenger RNA; siRNA, small interfering RNA; PLXDC1, plexin domain containing 1; NRP1, neuropilin-1.

## A novel regimen of therapy

The urgent need for improvement of efficacy in the HGSOC treatment is obvious, and many researchers have called attention to the novel approaches in diagnosis, monitoring, and management of patients with ovarian cancer. We have learned from the experience from therapy of hematologic cancers and several solid tumors that the individual approach to the treatment based on genetic, molecular, or metabolic signatures of the patients and the cancer itself usually results in better treatment efficacy and improved outcome. However, such individualization of therapy is much more difficult to be used in solid tumors, compared to hematologic malignancies, and ovarian cancer due to its unique biology is even more demanding and challenging target.

In the recent article devoted to OCSCs and OCSC-targeted treatment (470), we proposed that the novel complex standard of ovarian cancer therapy called the “DEPHENCE” system (“Dynamic PHarmacologic survEillaNCE”) should be worked out. In our opinion, it ought to be based on the following rules:

1. avoidance of monotherapy, as usually combination of several drugs directed against different targets, is more efficient and, if properly orchestrated, could be less toxic;
2. identification of the markers for pharmacologic compliance or resistance of the tumor and stratification of the patients according to the prognosis of treatment efficacy;
3. performing the sampling of the tumor (primary, metastatic, and recurrent) repetitively for characterization of genetic signature and TME features, which could change in the course of the disease and in the response to the treatment;
4. using the repeated biopsy of the tumor, but preferentially liquid biopsy, which enables to obtain more complex picture of growing tumor, as compared to standard biopsy the results of liquid biopsy do not depend on the site of the harvest of the sample;
5. such approach and individualization of the therapy could enable to restore the pharmacologic surveillance over the tumor that fits the actual status of both tumor and the patient;
6. every line of treatment should simultaneously target cancer cells, OCSCs, and elements of TME, as well as should generate potentialization of the patient’s immune status;
7. HGSOC molecular types and different phases of the disease need different approach to the therapy;
8. at the beginning, such therapy could allow for stabilization of the disease, hopefully enabling prolongation of PFS and OS; however, in a distant future the goal of this approach should be complete curation.

We think that the necessary components incorporated into the DEPHENCE system should also be

1. identification of the high-risk population of women (gene mutations, single-nucleotide polymorphisms, metabolic syndromes, and environmental factors);
2. searching for the techniques of early detection or even for the screening tools both in the high-risk and general populations;
3. searching for the infection factors responsible potentially for ovarian cancer origin (viruses, microbiome disturbances);
4. looking for prognosis biomarkers of ovarian cancer.

The practical implementation of the “DEPHENCE” system in the diagnosis and therapy of ovarian cancer is still awaiting, although the first signs of its use can be seen in the attempts to classify the molecular signatures of the tumors and TME components (158, 458, 471–476), to personalize therapy according to the tumor origin, histology, and most of all to genomic and epigenomic disturbances. The first such studies grouped HGSOC tumors T into four subtypes: C1, high stromal response; C2, high immune signature; C4, low stromal response; and C5, mesenchymal, with low immune signature. These subtypes differed in the extent of immune infiltration, desmoplasia, and EMT predisposition, and what could suggest different approach to the treatment, including immunotherapy, and patients from the C1 and C5 subtypes showed poor survival compared with other subtypes (3). Another genomic classification was proposed by The Cancer Genome Atlas Research Network, which, based on the genomic pattern, divided the ovarian cancer into four subtypes: mesenchymal, immunoreactive, proliferative, and differentiated. Mesenchymal and proliferative subtypes showed profound desmoplasia and invasive gene expression pattern, with limited immune infiltration and activation of stemness markers. Both were characterized by unfavorable prognosis. Immunoreactive subtype showed extensive immune infiltration and, similar to differentiated more mature tumors, had better prognosis (477–479). The next analysis of tumor genome identified three novel ovarian cancer subtypes named tumor-enriched, immune-enriched, and mixed. The meaning of these subtypes for therapy implies that tumor-enriched tumors should be treated with tumor killing therapy, whereas immune-enriched tumors with immunotherapy or mixture of both approaches (480). Molecular characterization of platinum-refractory and platinum-resistant ovarian tumors identified three tumor clusters: cluster 1 with overrepresentation of growth factor signaling pathways, cluster 2 with pathways regulating cell survival in hypoxic conditions and senescence, and cluster 3 related to cellular senescence. A possible treatment of choice for cluster 1 could be tyrosine kinase or angiokinase inhibitors, cluster 2 could theoretically response to mTOR inhibitors, whereas cluster 3 could be treated with the deacetylase inhibitors (87, 481, 482). Another single-cell transcriptome study revealed the heterogeneity of HGSOC, which was found to be composed of several cell clusters. The first one called EC1 showed gene enrichment for glycolysis/gluconeogenesis and ECM-receptor interactions. The EC2 subtype expressed genes, suggesting their origin from tube epithelium. The EC3 subtype showed overexpression of genes associated with function of ABC transporters, suggesting a potential to be a drug-resistant subtype. EC4 subtype was characterized by the immune response-related pathways indicating the activity of EC4 cells in immune response. The chemoresistance responsible genes were strongly represented in EC5 cell population (483).

Epigenomic analysis of immune-related lncRNAs revealed RNAs having the potential to divide the population of patients with ovarian cancer into high-risk and low-risk groups characterized by a shorter or longer overall survival (OS), respectively. High-risk score tumors were positively correlated with abundant representation of checkpoint and immunosuppressive molecules, indicating the group of patients with compromised anti-tumor immune response (484). The DNA methylation signatures represent another epigenetic point of interest in ovarian tumors. The hypomethylated upregulated tumor necrosis factor (*TNF*), estrogen receptor 1 (*ESR1*), mucin 1 (*MUC1*) genes, and hypermethylated downregulated forkhead box O1 (FOXO1) gene could serve as targets for epigenetic therapy and were correlated with patients’ prognosis (485).

According to the TME components, the four different CAF subsets (S1 to S4) were identified in ovarian tumors. The HGSOC of mesenchymal subtype, defined by stromal gene signatures and poor survival, had high numbers of CAF-S1 cells, which attracted and sustained immunosuppressive infiltration of Treg CD25+FoxP3+ T lymphocytes (475). The study of immunological profile of HGSOC showed the presence of activated‐immune and CAF‐immune subtypes. Activated-immune subtype showed anti-tumor features exemplified by active immune response and better prognosis. The CAF‐immune subtype was characterized by tumor‐promoting signals like, activated stroma, M2 macrophages, and a poor prognosis. The activated‐immune subtype was more likely than the CAF‐immune subtype to respond to checkpoint blockade immunotherapy (486).

The most painful problem in ovarian cancer therapy is the acquired chemoresistance following the initial good response to the first-line chemotherapy. Therefore, identification of the biomarkers of chemoresistance is one of the most important activities in ovarian cancer surveillance. The classic biomarkers of platinum and PARP chemosensitivity are the germinal and somatic mutations of *BRCA1/2* genes (487). However, the reversion mutations in *BRCA* genes and in other homologous recombination repair (HR) genes were found to be responsible for secondary resistance to platinum- and PARPi–based therapy (488, 489). On the basis of the homologous recombination deficiency, insertions and deletions, copy number changes, and mutational signatures, a combined predictor of platinum resistance, named DRDscore, was established, and, when validated in a cohort of patients with HGSOC, it reached sensitivity of 91% (490). Four miRNA biomarkers (miR-454-3p, miR-98-5p, miR-183-5p, and miR-22-3p) identified in ovarian cancer tissues were able to discriminate between platinum-sensitive and platinum-resistant patients with HGSOC (491). Treatment using PARPis results in acquired PARPi resistance. The reason for this is a promotion of STAT3 activity both in tumor cells and populations of immune and CAF cells, followed by creation of an immunosuppressive environment. Treatment of olaparib-resistant ovarian cancer cell line with napabucasin, the STAT3 inhibitor, improved PARPi sensitivity (492). Hypoxia and therapy-induced senescence are the key drivers of primary chemo-refractoriness and secondary chemoresistance of HGSOC (493). Hypoxic TME induces the M2-phenotype in TAMs, which, in turn, secrete exosomes containing miR-223 that, when transported into ovarian cancer cells, makes them chemoresistant (494). To overcome chemoresistance, there are plenty of different drug combinations tested in both experimental and clinical settings (**Tables 4**, **5**). Simultaneously, identification of potentially resistant tumors is of the utmost importance for successful therapy. Identification of ovarian cancer cells with high-stress signature and disturbed drug responsiveness could optimize the subsequent therapy to attenuate their function or eliminate them from the tumor (493, 495, 496). Moreover, as HGSOC tumors are characterized by temporal heterogeneity, the repetitive circulating tumor DNA (ctDNA)/CTCs testing should be performed to have the most actual picture of the disease.

The exploration of the infection factors in the origin or predisposition to ovarian cancer is also being realized in the analysis of microbiome and viral infections (497–499). Another field of intensive investigation is searching for prognostic biomarkers (500–503). It is a lot of work to do to safely and effectively combine different drugs, but the practical use of the “DEPHENCE” system philosophy could, in our opinion, lead doctors and researchers in proper direction.

## Author contributions

All authors contributed to the conception of the review. Data collection and analysis were performed by JW, MW, and EP. The first version of manuscript was written by JW and MW, and all authors commented on previous versions of the paper. EP and MW read and approved the final version of the manuscript, made linguistic corrections, and revised the text. All authors contributed to the article and approved the submitted version.

## References

[1] Kurman RJ Shih I-M. The dualistic model of ovarian carcinogenesis. Am J Pathol (2016) 186:733–47. doi: 10.1016/j.ajpath.2015.11.011 PMC5808151 27012190

[2] Survival rates for ovarian cancer (2022). Available at: https://www.cancer.org/cancer/ovarian-cancer/detection-diagnosis-staging/survival-rates.html.

[3] Tothill RW Tinker AV George J Brown R Fox SB Lade S. Novel molecular subtypes of serous and endometrioid ovarian cancer linked to clinical outcome. Clin Cancer Res (2008) 14:5198–208. doi: 10.1158/1078-0432.CCR-08-0196 18698038

[4] Muñoz-Galván S Carnero A. Targeting cancer stem cells to overcome therapy resistance in ovarian cancer. Cells (2020) 9:1402. doi: 10.3390/cells9061402 32512891 PMC7349391

[5] Wang X. Stem cells in tissues, organoids, and cancers. Cell Mol Life Sci (2019) 76:4043–70. doi: 10.1007/s00018-019-03199-x PMC6785598 31317205

[6] Batlle E Clevers H. Cancer stem cells revisited. Nat Med (2017) 23:1124–34. doi: 10.1038/nm.4409 28985214

[7] Tam WL Weinberg RA. The epigenetics of epithelial-mesenchymal plasticity in cancer. Nat Med (2013) 19:1438–49. doi: 10.1038/nm.3336 PMC4190672 24202396

[8] Berabez N Durand S Gabut M. Post-transcriptional regulations of cancer stem cell homeostasis. Curr Opin Oncol (2019) 31:100–7. doi: 10.1097/CCO.0000000000000503 30562314

[9] Marcucci F Bellone M Caserta CA Corti A. Pushing tumor cells towards a malignant phenotype: stimuli from the microenvironment, intercellular communications and alternative roads: stimuli promoting a malignant phenotype. Int J Cancer (2014) 135:1265–76. doi: 10.1002/ijc.28572 24174383

[10] Takebe N Miele L Harris PJ Jeong W Bando H Kahn M. Targeting notch, hedgehog, and wnt pathways in cancer stem cells: clinical update. Nat Rev Clin Oncol (2015) 12:445–64. doi: 10.1038/nrclinonc.2015.61 PMC4520755 25850553

[11] Taniguchi H Suzuki Y Natori Y. The evolving landscape of cancer stem cells and ways to overcome cancer heterogeneity. Cancers (2019) 11:532. doi: 10.3390/cancers11040532 31013960 PMC6520864

[12] Risson E Nobre AR Maguer-Satta V Aguirre-Ghiso JA. The current paradigm and challenges ahead for the dormancy of disseminated tumor cells. Nat Cancer (2020) 1:672–80. doi: 10.1038/s43018-020-0088-5 PMC7929485 33681821

[13] Hen O Barkan D. Dormant disseminated tumor cells and cancer stem/progenitor-like cells: similarities and opportunities. Semin Cancer Biol (2020) 60:157–65. doi: 10.1016/j.semcancer.2019.09.002 31491559

[14] Schewe DM Aguirre-Ghiso JA. ATF6α-Rheb-mTOR signaling promotes survival of dormant tumor cells *in vivo* . Proc Natl Acad Sci (2008) 105:10519–24. doi: 10.1073/pnas.0800939105 PMC2492459 18650380

[15] De Angelis M Francescangeli F Zeuner A. Breast cancer stem cells as drivers of tumor chemoresistance, dormancy and relapse: new challenges and therapeutic opportunities. Cancers (2019) 11:1569. doi: 10.3390/cancers11101569 31619007 PMC6826533

[16] Tjhay F Motohara T Tayama S Narantuya D Fujimoto K Guo J. CD 44 variant 6 is correlated with peritoneal dissemination and poor prognosis in patients with advanced epithelial ovarian cancer. Cancer Sci (2015) 106:1421–8. doi: 10.1111/cas.12765 PMC4638001 26250934

[17] Bourguignon LYW Peyrollier K Xia W Gilad E. Hyaluronan-CD44 interaction activates stem cell marker nanog, stat-3-mediated MDR1 gene expression, and ankyrin-regulated multidrug efflux in breast and ovarian tumor cells. J Biol Chem (2008) 283:17635–51. doi: 10.1074/jbc.M800109200 PMC2427357 18441325

[18] Zhang S Balch C Chan MW Lai H-C Matei D Schilder JM. Identification and characterization of ovarian cancer-initiating cells from primary human tumors. Cancer Res (2008) 68:4311–20. doi: 10.1158/0008-5472.CAN-08-0364 PMC2553722 18519691

[19] Grass GD Tolliver LB Bratoeva M Toole BP. CD147, CD44, and the epidermal growth factor receptor (EGFR) signaling pathway cooperate to regulate breast epithelial cell invasiveness. J Biol Chem (2013) 288:26089–104. doi: 10.1074/jbc.M113.497685 PMC3764812 23888049

[20] Motohara T Fujimoto K Tayama S Narantuya D Sakaguchi I Tashiro H. CD44 variant 6 as a predictive biomarker for distant metastasis in patients with epithelial ovarian cancer. Obstet Gynecol (2016) 127:1003–11. doi: 10.1097/AOG.0000000000001420 27159753

[21] Liu M Mor G Cheng H Xiang X Hui P Rutherford T. High frequency of putative ovarian cancer stem cells with CD44/CK19 coexpression is associated with decreased progression-free intervals in patients with recurrent epithelial ovarian cancer. Reprod Sci (2013) 20:605–15. doi: 10.1177/1933719112461183 PMC3635069 23171677

[22] Zhang J Chang B Liu J. CD44 standard form expression is correlated with high-grade and advanced-stage ovarian carcinoma but not prognosis. Hum Pathol (2013) 44:1882–9. doi: 10.1016/j.humpath.2013.02.016 PMC3752324 23664487

[23] Kar K Ghosh S Roy AK. A study of CD44 positive cancer cells in epithelial ovarian cancer and their correlation with P53 and Ki67. J Lab Physicians (2021) 13:050–7. doi: 10.1055/s-0041-1724235 PMC8154345 34054238

[24] Wu Y Wang T Xia L Zhang M. LncRNA WDFY3-AS2 promotes cisplatin resistance and the cancer stem cell in ovarian cancer by regulating hsa-miR-139-5p/SDC4 axis. Cancer Cell Int (2021) 21:284. doi: 10.1186/s12935-021-01993-x 34051810 PMC8164817

[25] Golebiewska A Brons NHC Bjerkvig R Niclou SP. Critical appraisal of the side population assay in stem cell and cancer stem cell research. Cell Stem Cell (2011) 8:136–47. doi: 10.1016/j.stem.2011.01.007 21295271

[26] Luo L Zeng J Liang B Zhao Z Sun L Cao D. Ovarian cancer cells with the CD117 phenotype are highly tumorigenic and are related to chemotherapy outcome. Exp Mol Pathol (2011) 91:596–602. doi: 10.1016/j.yexmp.2011.06.005 21787767

[27] Raspollini MR Amunni G Villanucci A Baroni G Taddei A Taddei GL. C-KIT expression and correlation with chemotherapy resistance in ovarian carcinoma: an immunocytochemical study. Ann Oncol (2004) 15:594–7. doi: 10.1093/annonc/mdh139 15033665

[28] Chen M Su J Feng C Liu Y Zhao L Tian Y. Chemokine CCL20 promotes the paclitaxel resistance of CD44 ^+^ CD117 ^+^ cells via the Notch1 signaling pathway in ovarian cancer. Mol Med Rep (2021) 24:635. doi: 10.3892/mmr.2021.12274 34278466 PMC8280726

[29] Stemberger-Papić S Vrdoljak-Mozetic D Ostojić DV Rubesa-Mihaljević R Krigtofić I Brncić-Fisher A. Expression of CD133 and CD117 in 64 serous ovarian cancer cases. Coll Antropol (2015) 39:745–53. 26898076

[30] Mazzoldi EL Pavan S Pilotto G Leone K Pagotto A Frezzini S. A juxtacrine/paracrine loop between c-kit and stem cell factor promotes cancer stem cell survival in epithelial ovarian cancer. Cell Death Dis (2019) 10:412. doi: 10.1038/s41419-019-1656-4 31138788 PMC6538673

[31] Roy L Bobbs A Sattler R Kurkewich JL Dausinas PB Nallathamby P. CD133 promotes adhesion to the ovarian cancer metastatic niche. Cancer Growth Metastasis (2018) 11:117906441876788. doi: 10.1177/1179064418767882 PMC5894897 29662326

[32] Xiang T Long H He L Han X Lin K Liang Z. Interleukin-17 produced by tumor microenvironment promotes self-renewal of CD133+ cancer stem-like cells in ovarian cancer. Oncogene (2015) 34:165–76. doi: 10.1038/onc.2013.537 24362529

[33] Baba T Convery PA Matsumura N Whitaker RS Kondoh E Perry T. Epigenetic regulation of CD133 and tumorigenicity of CD133+ ovarian cancer cells. Oncogene (2009) 28:209–18. doi: 10.1038/onc.2008.374 18836486

[34] Zhang J Guo X Chang DY Rosen DG Mercado-Uribe I Liu J. CD133 expression associated with poor prognosis in ovarian cancer. Mod Pathol (2012) 25:456–64. doi: 10.1038/modpathol.2011.170 PMC3855345 22080056

[35] Kusumbe AP Mali AM Bapat SA. CD133-expressing stem cells associated with ovarian metastases establish an endothelial hierarchy and contribute to tumor vasculature. Stem Cells (2009) 27:498–508. doi: 10.1634/stemcells.2008-0868 19253934

[36] Ferrandina G Martinelli E Petrillo M Prisco MG Zannoni G Sioletic S. CD133 antigen expression in ovarian cancer. BMC Cancer (2009) 9:221. doi: 10.1186/1471-2407-9-221 19583859 PMC3224735

[37] Qin Q Sun Y Fei M Zhang J Jia Y Gu M. Expression of putative stem marker nestin and CD133 in advanced serous ovarian cancer. Neoplasma (2012) 59:310–6. doi: 10.4149/neo_2012_040 22296500

[38] Abubaker K Luwor RB Zhu H McNally O Quinn MA Burns CJ. Inhibition of the JAK2/STAT3 pathway in ovarian cancer results in the loss of cancer stem cell-like characteristics and a reduced tumor burden. BMC Cancer (2014) 14:317. doi: 10.1186/1471-2407-14-317 24886434 PMC4025194

[39] Davidson B Holth A Hellesylt E Tan TZ Huang RY-J Tropé C. The clinical role of epithelial-mesenchymal transition and stem cell markers in advanced-stage ovarian serous carcinoma effusions. Hum Pathol (2015) 46:1–8. doi: 10.1016/j.humpath.2014.10.004 25455994

[40] Davidson B. CD24 is highly useful in differentiating high-grade serous carcinoma from benign and malignant mesothelial cells. Hum Pathol (2016) 58:123–7. doi: 10.1016/j.humpath.2016.08.005 27589896

[41] Gao M-Q Choi Y-P Kang S Youn JH Cho N-H. CD24+ cells from hierarchically organized ovarian cancer are enriched in cancer stem cells. Oncogene (2010) 29:2672–80. doi: 10.1038/onc.2010.35 20190812

[42] Nakamura K Terai Y Tanabe A Ono YJ Hayashi M Maeda K. CD24 expression is a marker for predicting clinical outcome and regulates the epithelial-mesenchymal transition in ovarian cancer via both the akt and ERK pathways. Oncol Rep (2017) 37:3189–200. doi: 10.3892/or.2017.5583 PMC5442399 28440503

[43] Runz S Keller S Rupp C Stoeck A Issa Y Koensgen D. Malignant ascites-derived exosomes of ovarian carcinoma patients contain CD24 and EpCAM. Gynecol Oncol (2007) 107:563–71. doi: 10.1016/j.ygyno.2007.08.064 17900673

[44] Fu J Shang Y Qian Z Hou J Yan F Liu G. Chimeric antigen receptor-T (CAR-T) cells targeting epithelial cell adhesion molecule (EpCAM) can inhibit tumor growth in ovarian cancer mouse model. J Vet Med Sci (2021) 83:241–7. doi: 10.1292/jvms.20-0455 PMC7972873 33328392

[45] Akhter MZ Sharawat SK Kumar V Kochat V Equbal Z Ramakrishnan M. Aggressive serous epithelial ovarian cancer is potentially propagated by EpCAM+CD45+ phenotype. Oncogene (2018) 37:2089–103. doi: 10.1038/s41388-017-0106-y 29379166

[46] The PLOS ONE Staff. Correction: the MyD88+ phenotype is an adverse prognostic factor in epithelial ovarian cancer. PloS One (2014) 9:e108833. doi: 10.1371/journal.pone.0108833 PMC4076208 24977712

[47] Liu W Zhang J Gan X Shen F Yang X Du N. LGR 5 promotes epithelial ovarian cancer proliferation, metastasis, and epithelial–mesenchymal transition through the Notch1 signaling pathway. Cancer Med (2018) 7:3132–42. doi: 10.1002/cam4.1485 PMC6051213 29777575

[48] Schindler AJ Watanabe A Howell SB. LGR5 and LGR6 in stem cell biology and ovarian cancer. Oncotarget (2018) 9:1346–55. doi: 10.18632/oncotarget.20178 PMC5787443 29416699

[49] Steg AD Bevis KS Katre AA Ziebarth A Dobbin ZC Alvarez RD. Stem cell pathways contribute to clinical chemoresistance in ovarian cancer. Clin Cancer Res (2012) 18:869–81. doi: 10.1158/1078-0432.CCR-11-2188 PMC3271164 22142828

[50] Silva IA Bai S McLean K Yang K Griffith K Thomas D. Aldehyde dehydrogenase in combination with CD133 defines angiogenic ovarian cancer stem cells that portend poor patient survival. Cancer Res (2011) 71:3991–4001. doi: 10.1158/0008-5472.CAN-10-3175 21498635 PMC3107359

[51] House CD Jordan E Hernandez L Ozaki M James JM Kim M. NFκB promotes ovarian tumorigenesis via classical pathways that support proliferative cancer cells and alternative pathways that support ALDH+ cancer stem–like cells. Cancer Res (2017) 77:6927–40. doi: 10.1158/0008-5472.CAN-17-0366 PMC5732863 29074539

[52] Liebscher CA Prinzler J Sinn BV Budczies J Denkert C Noske A. Aldehyde dehydrogenase 1/epidermal growth factor receptor coexpression is characteristic of a highly aggressive, poor-prognosis subgroup of high-grade serous ovarian carcinoma. Hum Pathol (2013) 44:1465–71. doi: 10.1016/j.humpath.2012.12.016 23465277

[53] Li Y Chen K Li L Li R Zhang J Ren W. Overexpression of SOX2 is involved in paclitaxel resistance of ovarian cancer via the PI3K/Akt pathway. Tumor Biol (2015) 36:9823–8. doi: 10.1007/s13277-015-3561-5 26159849

[54] Wen Y Hou Y Huang Z Cai J Wang Z. SOX2 is required to maintain cancer stem cells in ovarian cancer. Cancer Sci (2017) 108:719–31. doi: 10.1111/cas.13186 PMC5406610 28165651

[55] Ip CKM Li S-S Tang MYH Sy SKH Ren Y Shum HC. Stemness and chemoresistance in epithelial ovarian carcinoma cells under shear stress. Sci Rep (2016) 6:26788. doi: 10.1038/srep26788 27245437 PMC4887794

[56] Levasseur DN Wang J Dorschner MO Stamatoyannopoulos JA Orkin SH. Oct4 dependence of chromatin structure within the extended nanog locus in ES cells. Genes Dev (2008) 22:575–80. doi: 10.1101/gad.1606308 PMC2259027 18283123

[57] Peng S Maihle NJ Huang Y. Pluripotency factors Lin28 and Oct4 identify a sub-population of stem cell-like cells in ovarian cancer. Oncogene (2010) 29:2153–9. doi: 10.1038/onc.2009.500 20101213

[58] Fan Q Cai Q Xu Y. FOXM1 is a downstream target of LPA and YAP oncogenic signaling pathways in high grade serous ovarian cancer. Oncotarget (2015) 6:27688–99. doi: 10.18632/oncotarget.4280 PMC4695018 26299613

[59] Nagaraj AB Joseph P Kovalenko O Singh S Armstrong A Redline R. Critical role of wnt/β-catenin signaling in driving epithelial ovarian cancer platinum resistance. Oncotarget (2015) 6:23720–34. doi: 10.18632/oncotarget.4690 PMC4695147 26125441

[60] Zhou J Wang Y Wang Y Yin X He Y Chen L. FOXM1 modulates cisplatin sensitivity by regulating EXO1 in ovarian cancer. PloS One (2014) 9:e96989. doi: 10.1371/journal.pone.0096989 24824601 PMC4019642

[61] Fox RG Park FD Koechlein CS Kritzik M Reya T. Musashi signaling in stem cells and cancer. Annu Rev Cell Dev Biol (2015) 31:249–67. doi: 10.1146/annurev-cellbio-100814-125446 26566113

[62] Kudinov AE Karanicolas J Golemis EA Boumber Y. Musashi RNA-binding proteins as cancer drivers and novel therapeutic targets. Clin Cancer Res (2017) 23:2143–53. doi: 10.1158/1078-0432.CCR-16-2728 PMC5413399 28143872

[63] Chen P Li Q Yang Z. Musashi-1 expression is a prognostic factor in ovarian adenocarcinoma and correlates with ALDH-1 expression. Pathol Oncol Res (2015) 21:1133–40. doi: 10.1007/s12253-015-9943-6 25971681

[64] Chen H Liu J Wang H Cheng Q Zhou C Chen X. Inhibition of RNA-binding protein musashi-1 suppresses malignant properties and reverses paclitaxel resistance in ovarian carcinoma. J Cancer (2019) 10:1580–92. doi: 10.7150/jca.27352 PMC6485236 31031868

[65] Teeuwssen Fodde. Wnt signaling in ovarian cancer stemness, EMT, and therapy resistance. J Clin Med (2019) 8:1658. doi: 10.3390/jcm8101658 31614568 PMC6832489

[66] Su H-Y Lai H-C Lin Y-W Liu C-Y Chen C-K Chou Y-C. Epigenetic silencing of SFRP5 is related to malignant phenotype and chemoresistance of ovarian cancer through wnt signaling pathway. Int J Cancer (2010) 127:555–67. doi: 10.1002/ijc.25083 19957335

[67] Raghavan S Mehta P Xie Y Lei YL Mehta G. Ovarian cancer stem cells and macrophages reciprocally interact through the WNT pathway to promote pro-tumoral and malignant phenotypes in 3D engineered microenvironments. J Immunother Cancer (2019) 7:190. doi: 10.1186/s40425-019-0666-1 31324218 PMC6642605

[68] Chen Y Bieber MM Teng NNH. Hedgehog signaling regulates drug sensitivity by targeting ABC transporters ABCB1 and ABCG2 in epithelial ovarian cancer. Mol Carcinog (2014) 53:625–34. doi: 10.1002/mc.22015 23423781

[69] Rocconi R. Hedgehog signaling pathway regulates the growth of ovarian cancer spheroid forming cells. Int J Oncol (2011) 39:797–804. doi: 10.3892/ijo.2011.1093 21701772

[70] Song X Yan L Lu C Zhang C Zhu F Yang J. Activation of hedgehog signaling and its association with cisplatin resistance in ovarian epithelial tumors. Oncol Lett (2018) 15:5569–76. doi: 10.3892/ol.2018.8008 PMC5840551 29552194

[71] Kim S Kim B Song YS. Ascites modulates cancer cell behavior, contributing to tumor heterogeneity in ovarian cancer. Cancer Sci (2016) 107:1173–8. doi: 10.1111/cas.12987 PMC5021036 27297561

[72] Overholtzer M Zhang J Smolen GA Muir B Li W Sgroi DC. Transforming properties of *YAP* , a candidate oncogene on the chromosome 11q22 amplicon. Proc Natl Acad Sci (2006) 103:12405–10. doi: 10.1073/pnas.0605579103 PMC1533802 16894141

[73] Xia Y Chang T Wang Y Liu Y Li W Li M. YAP promotes ovarian cancer cell tumorigenesis and is indicative of a poor prognosis for ovarian cancer patients. PloS One (2014) 9:e91770. doi: 10.1371/journal.pone.0091770 24622501 PMC3951505

[74] Kubelac P Braicu C Raduly L Chiroi P Nutu A Cojocneanu R. Comprehensive analysis of the expression of key genes related to hippo signaling and their prognosis impact in ovarian cancer. Diagnostics (2021) 11:344. doi: 10.3390/diagnostics11020344 33669647 PMC7922135

[75] Park JT Chen X Tropè CG Davidson B Shih I-M Wang T-L. Notch3 overexpression is related to the recurrence of ovarian cancer and confers resistance to carboplatin. Am J Pathol (2010) 177:1087–94. doi: 10.2353/ajpath.2010.100316 PMC2928943 20671266

[76] McAuliffe SM Morgan SL Wyant GA Tran LT Muto KW Chen YS. Targeting notch, a key pathway for ovarian cancer stem cells, sensitizes tumors to platinum therapy. Proc Natl Acad Sci (2012) 109:E2939-48. doi: 10.1073/pnas.1206400109 23019585 PMC3491453

[77] Perez-Fidalgo JA Ortega B Simon S Samartzis EP Boussios S. NOTCH signalling in ovarian cancer angiogenesis. Ann Transl Med (2020) 8:1705–5. doi: 10.21037/atm-20-4497 PMC7812236 33490217

[78] Gonzalez-Torres C Gaytan-Cervantes J Vazquez-Santillan K Mandujano-Tinoco EA Ceballos-Cancino G Garcia-Venzor A. NF-κB participates in the stem cell phenotype of ovarian cancer cells. Arch Med Res (2017) 48:343–51. doi: 10.1016/j.arcmed.2017.08.001 28886875

[79] Harte MT Gorski JJ Savage KI Purcell JW Barros EM Burn PM. NF-κB is a critical mediator of BRCA1-induced chemoresistance. Oncogene (2014) 33:713–23. doi: 10.1038/onc.2013.10 PMC3917825 23435429

[80] Kaltschmidt C Banz-Jansen C Benhidjeb T Beshay M Förster C Greiner J. A role for NF-κB in organ specific cancer and cancer stem cells. Cancers (2019) 11:655. doi: 10.3390/cancers11050655 31083587 PMC6563002

[81] Lin S-C Liao Y-C Chen P-M Yang Y-Y Wang Y-H Tung S-L. Periostin promotes ovarian cancer metastasis by enhancing M2 macrophages and cancer-associated fibroblasts via integrin-mediated NF-κB and TGF-β2 signaling. J BioMed Sci (2022) 29:109. doi: 10.1186/s12929-022-00888-x 36550569 PMC9784270

[82] Zhao B Niu X Huang S Yang J Wei Y Wang X. TLR4 agonist and hypoxia synergistically promote the formation of TLR4/NF-κB/HIF-1α loop in human epithelial ovarian cancer. Anal Cell Pathol (2022) 2022:1–19. doi: 10.1155/2022/4201262 PMC9023210 35464826

[83] Shuang T Wang M Zhou Y Shi C. Over-expression of nuclear NF-κB1 and c-rel correlates with chemoresistance and prognosis of serous epithelial ovarian cancer. Exp Mol Pathol (2016) 100:139–44. doi: 10.1016/j.yexmp.2015.11.030 26683819

[84] Guo R-X Qiao Y-H Zhou Y Li L-X Shi H-R Chen K-S. Increased staining for phosphorylated AKT and nuclear factor-κB p65 and their relationship with prognosis in epithelial ovarian cancer. Pathol Int (2008) 58:749–56. doi: 10.1111/j.1440-1827.2008.02306.x 19067848

[85] Wang L Wang C Jin S Qu D Ying H. Expression of NF-κB and PTEN in primary epithelial ovarian carcinoma and the correlation with chemoresistance. Int J Clin Exp Pathol (2015) 8:10953–63. PMC4637628 26617813

[86] van Lieshout L van de Stolpe A van der Ploeg P Bowtell D de Hullu J Piek J. Signal transduction pathway activity in high-grade, serous ovarian carcinoma reveals a more favorable prognosis in tumors with low PI3K and high NF-κB pathway activity: a novel approach to a long-standing enigma. Cancers (2020) 12:2660. doi: 10.3390/cancers12092660 32961868 PMC7564278

[87] McDonald M Salinas E Devor E Newtson A Thiel K Goodheart M. Molecular characterization of non-responders to chemotherapy in serous ovarian cancer. Int J Mol Sci (2019) 20:1175. doi: 10.3390/ijms20051175 30866519 PMC6429334

[88] Liu L Salnikov AV Bauer N Aleksandrowicz E Labsch S Nwaeburu C. Triptolide reverses hypoxia-induced epithelial–mesenchymal transition and stem-like features in pancreatic cancer by NF-κB downregulation. Int J Cancer (2014) 134:2489–503. doi: 10.1002/ijc.28583 PMC4255690 24615157

[89] Qin J Liu Y Lu Y Liu M Li M Li J. Hypoxia-inducible factor 1 alpha promotes cancer stem cells-like properties in human ovarian cancer cells by upregulating SIRT1 expression. Sci Rep (2017) 7:10592. doi: 10.1038/s41598-017-09244-8 28878214 PMC5587562

[90] Daponte A Ioannou M Mylonis I Simos G Minas M Messinis IE. Prognostic significance of hypoxia-inducible factor 1 alpha(HIF-1alpha) expression in serous ovarian cancer: an immunohistochemical study. BMC Cancer (2008) 8:335. doi: 10.1186/1471-2407-8-335 19014607 PMC2651893

[91] Deying W Feng G Shumei L Hui Z Ming L Hongqing W. CAF-derived HGF promotes cell proliferation and drug resistance by up-regulating the c-Met/PI3K/Akt and GRP78 signalling in ovarian cancer cells. Biosci Rep (2017) 37:BSR20160470. doi: 10.1042/BSR20160470 28258248 PMC5469328

[92] Kwon Y Smith BD Zhou Y Kaufman MD Godwin AK. Effective inhibition of c-MET-mediated signaling, growth and migration of ovarian cancer cells is influenced by the ovarian tissue microenvironment. Oncogene (2015) 34:144–53. doi: 10.1038/onc.2013.539 PMC4067476 24362531

[93] Kalluri R Zeisberg M. Fibroblasts in cancer. Nat Rev Cancer (2006) 6:392–401. doi: 10.1038/nrc1877 16572188

[94] Park CW Kim K-S Bae S Son HK Myung P-K Hong HJ. Cytokine secretion profiling of human mesenchymal stem cells by antibody array. Int J Stem Cells (2009) 2:59–68. doi: 10.15283/ijsc.2009.2.1.59 24855521 PMC4021795

[95] Gorodetska I Kozeretska I Dubrovska A. *BRCA* genes: the role in genome stability, cancer stemness and therapy resistance. J Cancer (2019) 10:2109–27. doi: 10.7150/jca.30410 PMC6548160 31205572

[96] Parzych KR Klionsky DJ. An overview of autophagy: morphology, mechanism, and regulation. Antioxid Redox Signal (2014) 20:460–73. doi: 10.1089/ars.2013.5371 PMC3894687 23725295

[97] Deng J Bai X Feng X Ni J Beretov J Graham P. Inhibition of PI3K/Akt/mTOR signaling pathway alleviates ovarian cancer chemoresistance through reversing epithelial-mesenchymal transition and decreasing cancer stem cell marker expression. BMC Cancer (2019) 19:618. doi: 10.1186/s12885-019-5824-9 31234823 PMC6591840

[98] Hassan AA Artemenko M Tang MKS Shi Z Chen L-Y Lai H-C. Ascitic fluid shear stress in concert with hepatocyte growth factor drive stemness and chemoresistance of ovarian cancer cells via the c-Met-PI3K/Akt-miR-199a-3p signaling pathway. Cell Death Dis (2022) 13:537. doi: 10.1038/s41419-022-04976-6 35676254 PMC9177676

[99] Zi D Li Q Xu C Zhou Z-W Song G-B Hu C-B. CXCR4 knockdown enhances sensitivity of paclitaxel via the PI3K/Akt/mTOR pathway in ovarian carcinoma. Aging (2022) 14:4673–98. doi: 10.18632/aging.203241 PMC9217704 35681259

[100] Wang S Li Z Zhu G Hong L Hu C Wang K. RNA-Binding protein IGF2BP2 enhances circ_0000745 abundancy and promotes aggressiveness and stemness of ovarian cancer cells via the microRNA-3187-3p/ERBB4/PI3K/AKT axis. J Ovarian Res (2021) 14:154. doi: 10.1186/s13048-021-00917-7 34774079 PMC8590297

[101] Cai J Xu L Tang H Yang Q Yi X Fang Y. The role of the PTEN/PI3K/Akt pathway on prognosis in epithelial ovarian cancer: a meta-analysis. Oncol (2014) 19:528–35. doi: 10.1634/theoncologist.2013-0333 PMC4012960 24718516

[102] Zhang M Liu Y Yin Y Sun Z Wang Y Zhang Z. UBE2S promotes the development of ovarian cancer by promoting PI3K/AKT/mTOR signaling pathway to regulate cell cycle and apoptosis. Mol Med (2022) 28:62. doi: 10.1186/s10020-022-00489-2 35658829 PMC9166599

[103] Wang D Li C Zhang Y Wang M Jiang N Xiang L. Combined inhibition of PI3K and PARP is effective in the treatment of ovarian cancer cells with wild-type PIK3CA genes. Gynecol Oncol (2016) 142:548–56. doi: 10.1016/j.ygyno.2016.07.092 PMC5551048 27426307

[104] Izar B Tirosh I Stover EH Wakiro I Cuoco MS Alter I. A single-cell landscape of high-grade serous ovarian cancer. Nat Med (2020) 26:1271–9. doi: 10.1038/s41591-020-0926-0 PMC7723336 32572264

[105] Ruan Z Yang X Cheng W. OCT4 accelerates tumorigenesis through activating JAK/STAT signaling in ovarian cancer side population cells. Cancer Manag Res (2018) 11:389–99. doi: 10.2147/CMAR.S180418 PMC6314052 30643464

[106] McLean K Tan L Bolland DE Coffman LG Peterson LF Talpaz M. Leukemia inhibitory factor functions in parallel with interleukin-6 to promote ovarian cancer growth. Oncogene (2019) 38:1576–84. doi: 10.1038/s41388-018-0523-6 PMC6374186 30305729

[107] Shang A-Q Wu J Bi F Zhang Y-J Xu L-R Li L-L. Relationship between HER2 and JAK/STAT-SOCS3 signaling pathway and clinicopathological features and prognosis of ovarian cancer. Cancer Biol Ther (2017) 18:314–22. doi: 10.1080/15384047.2017.1310343 PMC5499756 28448787

[108] Kenny HA Chiang C-Y White EA Schryver EM Habis M Romero IL. Mesothelial cells promote early ovarian cancer metastasis through fibronectin secretion. J Clin Invest (2014) 124:4614–28. doi: 10.1172/JCI74778 PMC4191043 25202979

[109] Zhang Q Peng C. Cancer−associated fibroblasts regulate the biological behavior of cancer cells and stroma in gastric cancer (Review). Oncol Lett (2017) 15:691–98. doi: 10.3892/ol.2017.7385 PMC5772670 29399141

[110] Cardenas H Vieth E Lee J Segar M Liu Y Nephew KP. TGF-β induces global changes in DNA methylation during the epithelial-to-mesenchymal transition in ovarian cancer cells. Epigenetics (2014) 9:1461–72. doi: 10.4161/15592294.2014.971608 PMC4622747 25470663

[111] McLean K Gong Y Choi Y Deng N Yang K Bai S. Human ovarian carcinoma–associated mesenchymal stem cells regulate cancer stem cells and tumorigenesis via altered BMP production. J Clin Invest (2011) 121:3206–19. doi: 10.1172/JCI45273 PMC3148732 21737876

[112] Pasquet M Golzio M Mery E Rafii A Benabbou N Mirshahi P. Hospicells (ascites-derived stromal cells) promote tumorigenicity and angiogenesis. Int J Cancer (2009) 126:2090–101. doi: 10.1002/ijc.24886 19739074

[113] Li X Gao X Yuan J Wang F Xu X Wang C. The miR-33a-5p/CROT axis mediates ovarian cancer cell behaviors and chemoresistance via the regulation of the TGF-β signal pathway. Front Endocrinol (2022) 13:950345. doi: 10.3389/fendo.2022.950345 PMC9478117 36120434

[114] Yang D Sun Y Hu L Zheng H Ji P Pecot CV. Integrated analyses identify a master MicroRNA regulatory network for the mesenchymal subtype in serous ovarian cancer. Cancer Cell (2013) 23:186–99. doi: 10.1016/j.ccr.2012.12.020 PMC3603369 23410973

[115] Shang B Liu Y Jiang S Liu Y. Prognostic value of tumor-infiltrating FoxP3+ regulatory T cells in cancers: a systematic review and meta-analysis. Sci Rep (2015) 5:15179. doi: 10.1038/srep15179 26462617 PMC4604472

[116] Yue H Li W Chen R Wang J Lu X Li J. Stromal POSTN induced by TGF-β1 facilitates the migration and invasion of ovarian cancer. Gynecol Oncol (2021) 160:530–8. doi: 10.1016/j.ygyno.2020.11.026 33317907

[117] Zhao H Gao Y Chen Q Li J Ren M Zhao X. RAD51AP1 promotes progression of ovarian cancer via TGF-β/Smad signalling pathway. J Cell Mol Med (2021) 25:1927–38. doi: 10.1111/jcmm.15877 PMC7882964 33314567

[118] Gagno S Poletto E Bartoletti M Quartuccio L Romualdi C Garziera M. A TGF-β associated genetic score to define prognosis and platinum sensitivity in advanced epithelial ovarian cancer. Gynecol Oncol (2020) 156:233–42. doi: 10.1016/j.ygyno.2019.10.019 31711657

[119] Liang Q Xu Z Liu Y Peng B Cai Y Liu W. NR2F1 regulates TGF-β1-Mediated epithelial-mesenchymal transition affecting platinum sensitivity and immune response in ovarian cancer. Cancers (2022) 14:4639. doi: 10.3390/cancers14194639 36230565 PMC9563458

[120] Moffitt L Karimnia N Stephens A Bilandzic M. Therapeutic targeting of collective invasion in ovarian cancer. Int J Mol Sci (2019) 20:1466. doi: 10.3390/ijms20061466 30909510 PMC6471817

[121] Bryan BA Dennstedt E Mitchell DC Walshe TE Noma K Loureiro R. RhoA/ROCK signaling is essential for multiple aspects of VEGF-mediated angiogenesis. FASEB J (2010) 24:3186–95. doi: 10.1096/fj.09-145102 PMC2923346 20400538

[122] McGrail DJ Kieu QMN Dawson MR. Metastatic ovarian cancer cell malignancy is increased on soft matrices through a mechanosensitive Rho/ROCK pathway. J Cell Sci (2014) jcs:144378. doi: 10.1242/jcs.144378 PMC4058108 24741068

[123] Ogata S Morishige K-I Sawada K Hashimoto K Mabuchi S Kawase C. Fasudil inhibits lysophosphatidic acid-induced invasiveness of human ovarian cancer cells. Int J Gynecol Cancer (2009) 19:1473–80. doi: 10.1111/IGC.0b013e3181c03909 19955921

[124] Ohta T Takahashi T Shibuya T Amita M Henmi N Takahashi K. Inhibition of the Rho/ROCK pathway enhances the efficacy of cisplatin through the blockage of hypoxia-inducible factor-1α in human ovarian cancer cells. Cancer Biol Ther (2012) 13:25–33. doi: 10.4161/cbt.13.1.18440 22336585

[125] Meza-Perez S Randall TD. Immunological functions of the omentum. Trends Immunol (2017) 38:526–36. doi: 10.1016/j.it.2017.03.002 PMC5812451 28579319

[126] Liao J Qian F Tchabo N Mhawech-Fauceglia P Beck A Qian Z. Ovarian cancer spheroid cells with stem cell-like properties contribute to tumor generation, metastasis and chemotherapy resistance through hypoxia-resistant metabolism. PloS One (2014) 9:e84941. doi: 10.1371/journal.pone.0084941 24409314 PMC3883678

[127] Graves LE Ariztia EV Navari JR Matzel HJ Stack MS Fishman DA. Proinvasive properties of ovarian cancer ascites-derived membrane vesicles. Cancer Res (2004) 64:7045–9. doi: 10.1158/0008-5472.CAN-04-1800 15466198

[128] Milliken D Scotton C Raju S Balkwill F Wilson J. Analysis of chemokines and chemokine receptor expression in ovarian cancer ascites. Clin Cancer Res Off J Am Assoc Cancer Res (2002) 8:1108–14. 11948121

[129] Yamamoto CM Oakes ML Murakami T Muto MG Berkowitz RS Ng S-W. Comparison of benign peritoneal fluid- and ovarian cancer ascites-derived extracellular vesicle RNA biomarkers. J Ovarian Res (2018) 11:20. doi: 10.1186/s13048-018-0391-2 29499737 PMC5834862

[130] Bregenzer Horst Mehta Novak Repetto Mehta. The role of cancer stem cells and mechanical forces in ovarian cancer metastasis. Cancers (2019) 11:1008. doi: 10.3390/cancers11071008 31323899 PMC6679114

[131] Yeung T-L Leung CS Yip K-P Au Yeung CL Wong STC Mok SC. Cellular and molecular processes in ovarian cancer metastasis. a review in the theme: cell and molecular processes in cancer metastasis. Am J Physiol-Cell Physiol (2015) 309:C444–56. doi: 10.1152/ajpcell.00188.2015 PMC4593771 26224579

[132] Shishido A Mori S Yokoyama Y Hamada Y Minami K Qian Y. Mesothelial cells facilitate cancer stem−like properties in spheroids of ovarian cancer cells. Oncol Rep (2018) 40:2105–14. doi: 10.3892/or.2018.6605 30066911

[133] Friedl P Gilmour D. Collective cell migration in morphogenesis, regeneration and cancer. Nat Rev Mol Cell Biol (2009) 10:445–57. doi: 10.1038/nrm2720 19546857

[134] Montell DJ. Morphogenetic cell movements: diversity from modular mechanical properties. Science (2008) 322:1502–5. doi: 10.1126/science.1164073 19056976

[135] Mayor R Etienne-Manneville S. The front and rear of collective cell migration. Nat Rev Mol Cell Biol (2016) 17:97–109. doi: 10.1038/nrm.2015.14 26726037

[136] Cheung KJ Gabrielson E Werb Z Ewald AJ. Collective invasion in breast cancer requires a conserved basal epithelial program. Cell (2013) 155:1639–51. doi: 10.1016/j.cell.2013.11.029 PMC3941206 24332913

[137] Khalil AA Friedl P. Determinants of leader cells in collective cell migration. Integr Biol (2010) 2:568. doi: 10.1039/c0ib00052c 20886167

[138] Volkmer J-P Sahoo D Chin RK Ho PL Tang C Kurtova AV. Three differentiation states risk-stratify bladder cancer into distinct subtypes. Proc Natl Acad Sci (2012) 109:2078–83. doi: 10.1073/pnas.1120605109 PMC3277552 22308455

[139] Pandya P Orgaz JL Sanz-Moreno V. Actomyosin contractility and collective migration: may the force be with you. Curr Opin Cell Biol (2017) 48:87–96. doi: 10.1016/j.ceb.2017.06.006 28715714 PMC6137077

[140] Cheung KJ Padmanaban V Silvestri V Schipper K Cohen JD Fairchild AN. Polyclonal breast cancer metastases arise from collective dissemination of keratin 14-expressing tumor cell clusters. Proc Natl Acad Sci (2016) 113:E854-63. doi: 10.1073/pnas.1508541113 PMC4763783 26831077

[141] Zhu F Qian W Zhang H Liang Y Wu M Zhang Y. SOX2 is a marker for stem-like tumor cells in bladder cancer. Stem Cell Rep (2017) 9:429–37. doi: 10.1016/j.stemcr.2017.07.004 PMC5550032 28793245

[142] Papafotiou G Paraskevopoulou V Vasilaki E Kanaki Z Paschalidis N Klinakis A. KRT14 marks a subpopulation of bladder basal cells with pivotal role in regeneration and tumorigenesis. Nat Commun (2016) 7:11914. doi: 10.1038/ncomms11914 27320313 PMC4915139

[143] Kurtova AV Xiao J Mo Q Pazhanisamy S Krasnow R Lerner SP. Blocking PGE2-induced tumour repopulation abrogates bladder cancer chemoresistance. Nature (2015) 517:209–13. doi: 10.1038/nature14034 PMC4465385 25470039

[144] Neri S Ishii G Hashimoto H Kuwata T Nagai K Date H. Podoplanin-expressing cancer-associated fibroblasts lead and enhance the local invasion of cancer cells in lung adenocarcinoma: PDPN-CAFs lead and enhance cancer cell invasion. Int J Cancer (2015) 137:784–96. doi: 10.1002/ijc.29464 25648219

[145] Karimnia N Wilson AL Green E Matthews A Jobling TW Plebanski M. Chemoresistance is mediated by ovarian cancer leader cells *in vitro* . J Exp Clin Cancer Res (2021) 40:276. doi: 10.1186/s13046-021-02086-3 34470672 PMC8408956

[146] Cai J Tang H Xu L Wang X Yang C Ruan S. Fibroblasts in omentum activated by tumor cells promote ovarian cancer growth, adhesion and invasiveness. Carcinogenesis (2012) 33:20–9. doi: 10.1093/carcin/bgr230 22021907

[147] Rynne-Vidal A Jiménez-Heffernan J Fernández-Chacón C López-Cabrera M Sandoval P. The mesothelial origin of carcinoma associated-fibroblasts in peritoneal metastasis. Cancers (2015) 7:1994–2011. doi: 10.3390/cancers7040872 26426054 PMC4695872

[148] Zhang Y Tang H Cai J Zhang T Guo J Feng D. Ovarian cancer-associated fibroblasts contribute to epithelial ovarian carcinoma metastasis by promoting angiogenesis, lymphangiogenesis and tumor cell invasion. Cancer Lett (2011) 303:47–55. doi: 10.1016/j.canlet.2011.01.011 21310528

[149] Jang K Kim M Gilbert CA Simpkins F Ince TA Slingerland JM. VEGFA activates an epigenetic pathway upregulating ovarian cancer-initiating cells. EMBO Mol Med (2017) 9:304–18. doi: 10.15252/emmm.201606840 PMC5331266 28179359

[150] Kalluri R. The biology and function of fibroblasts in cancer. Nat Rev Cancer (2016) 16:582–98. doi: 10.1038/nrc.2016.73 27550820

[151] Sun W Fu S. Role of cancer-associated fibroblasts in tumor structure, composition and the microenvironment in ovarian cancer (Review). Oncol Lett (2019) 18:2173–78. doi: 10.3892/ol.2019.10587 PMC6676664 31452720

[152] Zeng L Wang X Wang F Zhao X Ding Y. Identification of a gene signature of cancer-associated fibroblasts to predict prognosis in ovarian cancer. Front Genet (2022) 13:925231. doi: 10.3389/fgene.2022.925231 35873482 PMC9298777

[153] Feng S Xu Y Dai Z Yin H Zhang K Shen Y. Integrative analysis from multicenter studies identifies a WGCNA-derived cancer-associated fibroblast signature for ovarian cancer. Front Immunol (2022) 13:951582. doi: 10.3389/fimmu.2022.951582 35874760 PMC9304893

[154] Zou R Jiang Q Jin T Chen M Yao L Ding H. Pan-cancer analyses and molecular subtypes based on the cancer-associated fibroblast landscape and tumor microenvironment infiltration characterization reveal clinical outcome and immunotherapy response in epithelial ovarian cancer. Front Immunol (2022) 13:956224. doi: 10.3389/fimmu.2022.956224 36032075 PMC9402225

[155] Sulaiman R De P Aske JC Lin X Dale A Koirala N. Patient-derived primary cancer-associated fibroblasts mediate resistance to anti-angiogenic drug in ovarian cancers. Biomedicines (2023) 11:112. doi: 10.3390/biomedicines11010112 36672620 PMC9855717

[156] Erez N Glanz S Raz Y Avivi C Barshack I. Cancer associated fibroblasts express pro-inflammatory factors in human breast and ovarian tumors. Biochem Biophys Res Commun (2013) 437:397–402. doi: 10.1016/j.bbrc.2013.06.089 23831470

[157] Ko SY Barengo N Ladanyi A Lee J-S Marini F Lengyel E. HOXA9 promotes ovarian cancer growth by stimulating cancer-associated fibroblasts. J Clin Invest (2012) 122:3603–17. doi: 10.1172/JCI62229 PMC3461910 22945634

[158] Ahmed N Escalona R Leung D Chan E Kannourakis G. Tumour microenvironment and metabolic plasticity in cancer and cancer stem cells: perspectives on metabolic and immune regulatory signatures in chemoresistant ovarian cancer stem cells. Semin Cancer Biol (2018) 53:265–81. doi: 10.1016/j.semcancer.2018.10.002 30317036

[159] Kershaw EE Flier JS. Adipose tissue as an endocrine organ. J Clin Endocrinol Metab (2004) 89:2548–56. doi: 10.1210/jc.2004-0395 15181022

[160] Nieman KM Kenny HA Penicka CV Ladanyi A Buell-Gutbrod R Zillhardt MR. Adipocytes promote ovarian cancer metastasis and provide energy for rapid tumor growth. Nat Med (2011) 17:1498–503. doi: 10.1038/nm.2492 PMC4157349 22037646

[161] Mukherjee A Chiang C-Y Daifotis HA Nieman KM Fahrmann JF Lastra RR. Adipocyte-induced FABP4 expression in ovarian cancer cells promotes metastasis and mediates carboplatin resistance. Cancer Res (2020) 80:1748–61. doi: 10.1158/0008-5472.CAN-19-1999 PMC10656748 32054768

[162] Li J Condello S Thomes-Pepin J Ma X Xia Y Hurley TD. Lipid desaturation is a metabolic marker and therapeutic target of ovarian cancer stem cells. Cell Stem Cell (2017) 20:303–314.e5. doi: 10.1016/j.stem.2016.11.004 28041894 PMC5337165

[163] Zhou Z Lu Y Wang Y Du L Zhang Y Tao J. Let-7c regulates proliferation and osteodifferentiation of human adipose-derived mesenchymal stem cells under oxidative stress by targeting SCD-1. Am J Physiol-Cell Physiol (2019) 316:C57–69. doi: 10.1152/ajpcell.00211.2018 30379578

[164] Cai Y Wang J Zhang L Wu D Yu D Tian X. Expressions of fatty acid synthase and HER2 are correlated with poor prognosis of ovarian cancer. Med Oncol (2015) 32:391. doi: 10.1007/s12032-014-0391-z 25433947 PMC4247847

[165] Cho JA Park H Lim EH Kim KH Choi JS Lee JH. Exosomes from ovarian cancer cells induce adipose tissue-derived mesenchymal stem cells to acquire the physical and functional characteristics of tumor-supporting myofibroblasts. Gynecol Oncol (2011) 123:379–86. doi: 10.1016/j.ygyno.2011.08.005 21903249

[166] Coffman LG Choi Y-J McLean K Allen BL di Magliano MP Buckanovich RJ. Human carcinoma-associated mesenchymal stem cells promote ovarian cancer chemotherapy resistance via a BMP4/HH signaling loop. Oncotarget (2016) 7:6916–32. doi: 10.18632/oncotarget.6870 PMC4872758 26755648

[167] Wen Y Guo Y Huang Z Cai J Wang Z. Adipose-derived mesenchymal stem cells attenuate cisplatin-induced apoptosis in epithelial ovarian cancer cells. Mol Med Rep (2017) 16:9587–92. doi: 10.3892/mmr.2017.7783 29039536

[168] Spaeth EL Dembinski JL Sasser AK Watson K Klopp A Hall B. Mesenchymal stem cell transition to tumor-associated fibroblasts contributes to fibrovascular network expansion and tumor progression. PloS One (2009) 4:e4992. doi: 10.1371/journal.pone.0004992 19352430 PMC2661372

[169] Touboul C Lis R Al Farsi H Raynaud CM Warfa M Althawadi H. Mesenchymal stem cells enhance ovarian cancer cell infiltration through IL6 secretion in an amniochorionic membrane based 3D model. J Transl Med (2013) 11:28. doi: 10.1186/1479-5876-11-28 23369187 PMC3582577

[170] Bu S Wang Q Zhang Q Sun J He B Xiang C. Human endometrial mesenchymal stem cells exhibit intrinsic anti-tumor properties on human epithelial ovarian cancer cells. Sci Rep (2016) 6:37019. doi: 10.1038/srep37019 27845405 PMC5109482

[171] Gu Z-W He Y-F Wang W-J Tian Q Di W. MiR-1180 from bone marrow-derived mesenchymal stem cells inducesglycolysis and chemoresistance in ovarian cancer cells by upregulating the wnt signalingpathway. J Zhejiang Univ-Sci B (2019) 20:219–37. doi: 10.1631/jzus.B1800190 PMC6421125 30829010

[172] Chen X Ying X Wang X Wu X Zhu Q Wang X. Exosomes derived from hypoxic epithelial ovarian cancer deliver microRNA-940 to induce macrophage M2 polarization. Oncol Rep (2017) 38:522–8. doi: 10.3892/or.2017.5697 28586039

[173] Yin M Li X Tan S Zhou HJ Ji W Bellone S. Tumor-associated macrophages drive spheroid formation during early transcoelomic metastasis of ovarian cancer. J Clin Invest (2016) 126:4157–73. doi: 10.1172/JCI87252 PMC5096908 27721235

[174] Mantovani A Sozzani S Locati M Allavena P Sica A. Macrophage polarization: tumor-associated macrophages as a paradigm for polarized M2 mononuclear phagocytes. Trends Immunol (2002) 23:549–55. doi: 10.1016/S1471-4906(02)02302-5 12401408

[175] Duluc D Delneste Y Tan F Moles M-P Grimaud L Lenoir J. Tumor-associated leukemia inhibitory factor and IL-6 skew monocyte differentiation into tumor-associated macrophage-like cells. Blood (2007) 110:4319–30. doi: 10.1182/blood-2007-02-072587 17848619

[176] Masoumi Moghaddam S Amini A Morris DL Pourgholami MH. Significance of vascular endothelial growth factor in growth and peritoneal dissemination of ovarian cancer. Cancer Metastasis Rev (2012) 31:143–62. doi: 10.1007/s10555-011-9337-5 PMC3350632 22101807

[177] Zeng X-Y Xie H Yuan J Jiang X-Y Yong J-H Zeng D. M2-like tumor-associated macrophages-secreted EGF promotes epithelial ovarian cancer metastasis via activating EGFR-ERK signaling and suppressing lncRNA LIMT expression. Cancer Biol Ther (2019) 20:956–66. doi: 10.1080/15384047.2018.1564567 PMC6606001 31062668

[178] Song M Yeku OO Rafiq S Purdon T Dong X Zhu L. Tumor derived UBR5 promotes ovarian cancer growth and metastasis through inducing immunosuppressive macrophages. Nat Commun (2020) 11:6298. doi: 10.1038/s41467-020-20140-0 33293516 PMC7722725

[179] Macciò A Gramignano G Cherchi MC Tanca L Melis L Madeddu C. Role of M1-polarized tumor-associated macrophages in the prognosis of advanced ovarian cancer patients. Sci Rep (2020) 10:6096. doi: 10.1038/s41598-020-63276-1 32269279 PMC7142107

[180] Tan Q Liu H Xu J Mo Y Dai F. Integrated analysis of tumor-associated macrophage infiltration and prognosis in ovarian cancer. Aging (2021) 13:23210–32. doi: 10.18632/aging.203613 PMC8544311 34633990

[181] Jiang S Yang Y Zhang Y Ye Q Song J Zheng M. Overexpression of CAPG is associated with poor prognosis and immunosuppressive cell infiltration in ovarian cancer. Dis Markers (2022) 2022:1–18. doi: 10.1155/2022/9719671 PMC8849939 35186171

[182] Wang H Yung MMH Ngan HYS Chan KKL Chan DW. The impact of the tumor microenvironment on macrophage polarization in cancer metastatic progression. Int J Mol Sci (2021) 22:6560. doi: 10.3390/ijms22126560 34207286 PMC8235734

[183] You Y Li Y Li M Lei M Wu M Qu Y. Ovarian cancer stem cells promote tumour immune privilege and invasion via CCL5 and regulatory T cells. Clin Exp Immunol (2017) 191:60–73. doi: 10.1111/cei.13044 28868628 PMC5721255

[184] Knutson KL Maurer MJ Preston CC Moysich KB Goergen K Hawthorne KM. Regulatory T cells, inherited variation, and clinical outcome in epithelial ovarian cancer. Cancer Immunol Immunother (2015) 64:1495–504. doi: 10.1007/s00262-015-1753-x PMC4651184 26298430

[185] Toker A Nguyen LT Stone SC Yang SYC Katz SR Shaw PA. Regulatory T cells in ovarian cancer are characterized by a highly activated phenotype distinct from that in melanoma. Clin Cancer Res (2018) 24:5685–96. doi: 10.1158/1078-0432.CCR-18-0554 30065096

[186] Marchenko S Piwonski I Hoffmann I Sinn BV Kunze CA Monjé N. Prognostic value of regulatory T cells and T helper 17 cells in high grade serous ovarian carcinoma. J Cancer Res Clin Oncol (2022) 149:2523–36. doi: 10.1007/s00432-022-04101-2 PMC10129928 35763108

[187] Curiel TJ Coukos G Zou L Alvarez X Cheng P Mottram P. Specific recruitment of regulatory T cells in ovarian carcinoma fosters immune privilege and predicts reduced survival. Nat Med (2004) 10:942–9. doi: 10.1038/nm1093 15322536

[188] Sato E Olson SH Ahn J Bundy B Nishikawa H Qian F. Intraepithelial CD8 ^+^ tumor-infiltrating lymphocytes and a high CD8 ^+^ /regulatory T cell ratio are associated with favorable prognosis in ovarian cancer. Proc Natl Acad Sci (2005) 102:18538–43. doi: 10.1073/pnas.0509182102 PMC1311741 16344461

[189] Bamias A Koutsoukou V Terpos E Tsiatas ML Liakos C Tsitsilonis O. Correlation of NK T-like CD3+CD56+ cells and CD4+CD25+(hi) regulatory T cells with VEGF and TNFα in ascites from advanced ovarian cancer: association with platinum resistance and prognosis in patients receiving first-line, platinum-based chemotherapy. Gynecol Oncol (2008) 108:421–7. doi: 10.1016/j.ygyno.2007.10.018 18036640

[190] Woo EY Chu CS Goletz TJ Schlienger K Yeh H Coukos G. Regulatory CD4(+)CD25(+) T cells in tumors from patients with early-stage non-small cell lung cancer and late-stage ovarian cancer. Cancer Res (2001) 61:4766–72. 11406550

[191] Labidi-Galy SI Sisirak V Meeus P Gobert M Treilleux I Bajard A. Quantitative and functional alterations of plasmacytoid dendritic cells contribute to immune tolerance in ovarian cancer. Cancer Res (2011) 71:5423–34. doi: 10.1158/0008-5472.CAN-11-0367 21697280

[192] Huarte E Cubillos-Ruiz JR Nesbeth YC Scarlett UK Martinez DG Buckanovich RJ. Depletion of dendritic cells delays ovarian cancer progression by boosting antitumor immunity. Cancer Res (2008) 68:7684–91. doi: 10.1158/0008-5472.CAN-08-1167 PMC2742361 18768667

[193] Truxova I Kasikova L Hensler M Skapa P Laco J Pecen L. Mature dendritic cells correlate with favorable immune infiltrate and improved prognosis in ovarian carcinoma patients. J Immunother Cancer (2018) 6:139. doi: 10.1186/s40425-018-0446-3 30526667 PMC6288908

[194] Zou W Machelon V Coulomb-L’Hermin A Borvak J Nome F Isaeva T. Stromal-derived factor-1 in human tumors recruits and alters the function of plasmacytoid precursor dendritic cells. Nat Med (2001) 7:1339–46. doi: 10.1038/nm1201-1339 11726975

[195] Krempski J Karyampudi L Behrens MD Erskine CL Hartmann L Dong H. Tumor-infiltrating programmed death receptor-1+ dendritic cells mediate immune suppression in ovarian cancer. J Immunol (2011) 186:6905–13. doi: 10.4049/jimmunol.1100274 PMC3110549 21551365

[196] Wertel I Polak G Bednarek W Barczyński B Roliński J Kotarski J. Dendritic cell subsets in the peritoneal fluid and peripheral blood of women suffering from ovarian cancer. Cytometry B Clin Cytom (2008) 74B:251–8. doi: 10.1002/cyto.b.20410 18302193

[197] Wei S Kryczek I Zou L Daniel B Cheng P Mottram P. Plasmacytoid dendritic cells induce CD8+ regulatory T cells in human ovarian carcinoma. Cancer Res (2005) 65:5020–6. doi: 10.1158/0008-5472.CAN-04-4043 15958543

[198] Yanagisawa K Exley MA Jiang X Ohkochi N Taniguchi M Seino K. Hyporesponsiveness to natural killer T-cell ligand α-galactosylceramide in cancer-bearing state mediated by CD11b+ gr-1+ cells producing nitric oxide. Cancer Res (2006) 66:11441–6. doi: 10.1158/0008-5472.CAN-06-0944 17145891

[199] Hart KM Byrne KT Molloy MJ Usherwood EM Berwin B. IL-10 immunomodulation of myeloid cells regulates a murine model of ovarian cancer. Front Immunol (2011) 2:29. doi: 10.3389/fimmu.2011.00029 22566819 PMC3342001

[200] Obermajer N Muthuswamy R Odunsi K Edwards RP Kalinski P. PGE2-induced CXCL12 production and CXCR4 expression controls the accumulation of human MDSCs in ovarian cancer environment. Cancer Res (2011) 71:7463–70. doi: 10.1158/0008-5472.CAN-11-2449 PMC4993027 22025564

[201] Pi R Yang Y Hu X Li H Shi H Liu Y. Dual mTORC1/2 inhibitor AZD2014 diminishes myeloid-derived suppressor cells accumulation in ovarian cancer and delays tumor growth. Cancer Lett (2021) 523:72–81. doi: 10.1016/j.canlet.2021.09.017 34560229

[202] Komura N Mabuchi S Shimura K Yokoi E Kozasa K Kuroda H. The role of myeloid-derived suppressor cells in increasing cancer stem-like cells and promoting PD-L1 expression in epithelial ovarian cancer. Cancer Immunol Immunother (2020) 69:2477–99. doi: 10.1007/s00262-020-02628-2 PMC11027471 32561967

[203] Okła K Czerwonka A Wawruszak A Bobiński M Bilska M Tarkowski R. Clinical relevance and immunosuppressive pattern of circulating and infiltrating subsets of myeloid-derived suppressor cells (MDSCs) in epithelial ovarian cancer. Front Immunol (2019) 10:691. doi: 10.3389/fimmu.2019.00691 31001284 PMC6456713

[204] Wu L Deng Z Peng Y Han L Liu J Wang L. Ascites-derived IL-6 and IL-10 synergistically expand CD14+HLA-DR-/low myeloid-derived suppressor cells in ovarian cancer patients. Oncotarget (2017) 8:76843–56. doi: 10.18632/oncotarget.20164 PMC5652747 29100353

[205] Lee J Botesteanu D Tomita Y Yuno A Lee M Kohn E. Patients with BRCA mutated ovarian cancer may have fewer circulating MDSC and more peripheral CD8+ T cells compared with women with BRCA wild−type disease during the early disease course. Oncol Lett (2019) 18:3914–24. doi: 10.3892/ol.2019.10731 PMC6732977 31516602

[206] Kumar SM Liu S Lu H Zhang H Zhang PJ Gimotty PA. Acquired cancer stem cell phenotypes through Oct4-mediated dedifferentiation. Oncogene (2012) 31:4898–911. doi: 10.1038/onc.2011.656 PMC3343184 22286766

[207] Roy Choudhury A Gupta S Chaturvedi PK Kumar N Pandey D. Mechanobiology of cancer stem cells and their niche. Cancer Microenviron (2019) 12:17–27. doi: 10.1007/s12307-019-00222-4 31004332 PMC6529500

[208] Klymenko Y Kim O Stack M. Complex determinants of epithelial: mesenchymal phenotypic plasticity in ovarian cancer. Cancers (2017) 9:104. doi: 10.3390/cancers9080104 28792442 PMC5575607

[209] Soriano AA de Cristofaro T Di Palma T Dotolo S Gokulnath P Izzo A. PAX8 expression in high-grade serous ovarian cancer positively regulates attachment to ECM via integrin β3. Cancer Cell Int (2019) 19:303. doi: 10.1186/s12935-019-1022-8 31832016 PMC6865034

[210] van der Steen SCHA Bulten J Van de Vijver KK van Kuppevelt TH Massuger LFAG. Changes in the extracellular matrix are associated with the development of serous tubal intraepithelial carcinoma into high-grade serous carcinoma. Int J Gynecol Cancer (2017) 27:1072–81. doi: 10.1097/IGC.0000000000000933 28333845

[211] Vallen MJE Massuger LFAG ten Dam GB Bulten J van Kuppevelt TH. Highly sulfated chondroitin sulfates, a novel class of prognostic biomarkers in ovarian cancer tissue. Gynecol Oncol (2012) 127:202–9. doi: 10.1016/j.ygyno.2012.06.022 22733095

[212] Ojasalu K Brehm C Hartung K Nischak M Finkernagel F Rexin P. Upregulation of mesothelial genes in ovarian carcinoma cells is associated with an unfavorable clinical outcome and the promotion of cancer cell adhesion. Mol Oncol (2020) 14:2142–62. doi: 10.1002/1878-0261.12749 PMC7463315 32533757

[213] Gutwein P Stoeck A Riedle S Gast D Runz S Condon TP. Cleavage of L1 in exosomes and apoptotic membrane vesicles released from ovarian carcinoma cells. Clin Cancer Res (2005) 11:2492–501. doi: 10.1158/1078-0432.CCR-04-1688 15814625

[214] Mitra AK Zillhardt M Hua Y Tiwari P Murmann AE Peter ME. MicroRNAs reprogram normal fibroblasts into cancer-associated fibroblasts in ovarian cancer. Cancer Discovery (2012) 2:1100–8. doi: 10.1158/2159-8290.CD-12-0206 PMC3685866 23171795

[215] Au Yeung CL Co N-N Tsuruga T Yeung T-L Kwan S-Y Leung CS. Exosomal transfer of stroma-derived miR21 confers paclitaxel resistance in ovarian cancer cells through targeting APAF1. Nat Commun (2016) 7:11150. doi: 10.1038/ncomms11150 27021436 PMC4820618

[216] Wu Q Wu X Ying X Zhu Q Wang X Jiang L. Suppression of endothelial cell migration by tumor associated macrophage-derived exosomes is reversed by epithelial ovarian cancer exosomal lncRNA. Cancer Cell Int (2017) 17:62. doi: 10.1186/s12935-017-0430-x 28592924 PMC5461704

[217] Lopatina T Gai C Deregibus MC Kholia S Camussi G. Cross talk between cancer and mesenchymal stem cells through extracellular vesicles carrying nucleic acids. Front Oncol (2016) 6:125. doi: 10.3389/fonc.2016.00125 27242964 PMC4876347

[218] Alharbi M Lai A Guanzon D Palma C Zuñiga F Perrin L. Ovarian cancer-derived exosomes promote tumour metastasis *in vivo*: an effect modulated by the invasiveness capacity of their originating cells. Clin Sci (2019) 133:1401–19. doi: 10.1042/CS20190082 31227603

[219] Qu Q Liu L Cui Y Chen Y Wang Y Wang Y. Exosomes from human omental adipose-derived mesenchymal stem cells secreted into ascites promote peritoneal metastasis of epithelial ovarian cancer. Cells (2022) 11:3392. doi: 10.3390/cells11213392 36359787 PMC9655202

[220] Qiu L Wang J Chen M Chen F Tu W. Exosomal microRNA−146a derived from mesenchymal stem cells increases the sensitivity of ovarian cancer cells to docetaxel and taxane via a LAMC2−mediated PI3K/Akt axis. Int J Mol Med (2020) 46:609–20. doi: 10.3892/ijmm.2020.4634 PMC7307828 32626953

[221] Vera N Acuña-Gallardo S Grünenwald F Caceres-Verschae A Realini O Acuña R. Small extracellular vesicles released from ovarian cancer spheroids in response to cisplatin promote the pro-tumorigenic activity of mesenchymal stem cells. Int J Mol Sci (2019) 20:4972. doi: 10.3390/ijms20204972 31600881 PMC6834150

[222] Luo H Zhou Y Zhang J Zhang Y Long S Lin X. NK cell-derived exosomes enhance the anti-tumor effects against ovarian cancer by delivering cisplatin and reactivating NK cell functions. Front Immunol (2023) 13:1087689. doi: 10.3389/fimmu.2022.1087689 36741396 PMC9892755

[223] Zhang X Wang J Liu N Wu W Li H Chen J. Molecular mechanism of CD163+ tumor-associated macrophage (TAM)-derived exosome-induced cisplatin resistance in ovarian cancer ascites. Ann Transl Med (2022) 10:1014–4. doi: 10.21037/atm-22-4267 PMC9577761 36267748

[224] Shnaider PV Petrushanko IYU Aleshikova OI Babaeva NA Ashrafyan LA Borovkova EI. Expression level of CD117 (KIT) on ovarian cancer extracellular vesicles correlates with tumor aggressiveness. Front Cell Dev Biol (2023) 11:1057484. doi: 10.3389/fcell.2023.1057484 36875773 PMC9978408

[225] Zhang W Ou X Wu X. Proteomics profiling of plasma exosomes in epithelial ovarian cancer: a potential role in the coagulation cascade, diagnosis and prognosis. Int J Oncol (2019) 54:1719–33. doi: 10.3892/ijo.2019.4742 PMC6438431 30864689

[226] Cai J Gong L Li G Guo J Yi X Wang Z. Exosomes in ovarian cancer ascites promote epithelial–mesenchymal transition of ovarian cancer cells by delivery of miR-6780b-5p. Cell Death Dis (2021) 12:210. doi: 10.1038/s41419-021-03490-5 33627627 PMC7904844

[227] Keng. Exosomes in the ascites of ovarian cancer patients: origin and effects on anti-tumor immunity. Oncol Rep (2011) 25:749–62. doi: 10.3892/or.2010.1119 21181093

[228] Taylor DD Gercel-Taylor C. MicroRNA signatures of tumor-derived exosomes as diagnostic biomarkers of ovarian cancer. Gynecol Oncol (2008) 110:13–21. doi: 10.1016/j.ygyno.2008.04.033 18589210

[229] Shimizu A Sawada K Kobayashi M Yamamoto M Yagi T Kinose Y. Exosomal CD47 plays an essential role in immune evasion in ovarian cancer. Mol Cancer Res (2021) 19:1583–95. doi: 10.1158/1541-7786.MCR-20-0956 34016744

[230] Kanlikilicer P Bayraktar R Denizli M Rashed MH Ivan C Aslan B. Corrigendum to ‘Exosomal miRNA confers chemo resistance via targeting Cav1/p-gp/M2-type macrophage axis in ovarian cancer’ [EBioMedicine 38 (2018) 100–112]. EBioMedicine (2020) 52:102630. doi: 10.1016/j.ebiom.2020.102630 31972470 PMC6974767

[231] Qiu J-J Lin X-J Tang X-Y Zheng T-T Lin Y-Y Hua K-Q. Exosomal Metastasis−Associated lung adenocarcinoma transcript 1 promotes angiogenesis and predicts poor prognosis in epithelial ovarian cancer. Int J Biol Sci (2018) 14:1960–73. doi: 10.7150/ijbs.28048 PMC6299373 30585260

[232] Chen L Wang K Li L Zheng B Zhang Q Zhang F. Plasma exosomal *miR-1260a, miR-7977* and *miR-192-5p* as diagnostic biomarkers in epithelial ovarian cancer. Future Oncol (2022) 18:2919–31. doi: 10.2217/fon-2022-0321 35893704

[233] Xiong C Sun Z Yu J Lin Y. Exosome component 4 promotes epithelial ovarian cancer cell proliferation, migration, and invasion via the wnt pathway. Front Oncol (2021) 11:797968. doi: 10.3389/fonc.2021.797968 34956910 PMC8692763

[234] Zhu Z Chen Z Wang M Zhang M Chen Y Yang X. Detection of plasma exosomal miRNA-205 as a biomarker for early diagnosis and an adjuvant indicator of ovarian cancer staging. J Ovarian Res (2022) 15:27. doi: 10.1186/s13048-022-00961-x 35183243 PMC8858566

[235] Li S-S Ma J Wong AST. Chemoresistance in ovarian cancer: exploiting cancer stem cell metabolism. J Gynecol Oncol (2018) 29:e32. doi: 10.3802/jgo.2018.29.e32 29468856 PMC5823988

[236] Majmundar AJ Wong WJ Simon MC. Hypoxia-inducible factors and the response to hypoxic stress. Mol Cell (2010) 40:294–309. doi: 10.1016/j.molcel.2010.09.022 20965423 PMC3143508

[237] Vander Linden C Corbet C. Therapeutic targeting of cancer stem cells: integrating and exploiting the acidic niche. Front Oncol (2019) 9:159. doi: 10.3389/fonc.2019.00159 30941310 PMC6433943

[238] Corbet C Feron O. Tumour acidosis: from the passenger to the driver’s seat. Nat Rev Cancer (2017) 17:577–93. doi: 10.1038/nrc.2017.77 28912578

[239] Toutirais O Chartier P Dubois D Bouet F Lévêque J Catros-Quemener V. Constitutive expression of TGF-bêta1, interleukin-6 and interleukin-8 by tumor cells as a major component of immune escape in human ovarian carcinoma. Eur Cytokine Netw (2003) 14:246–55. 14715418

[240] Mills C. M1 and M2 macrophages: oracles of health and disease. Crit Rev Immunol (2012) 32:463–88. doi: 10.1615/CritRevImmunol.v32.i6.10 23428224

[241] Naldini A Morena E Pucci A Miglietta D Riboldi E Sozzani S. Hypoxia affects dendritic cell survival: role of the hypoxia-inducible factor-1α and lipopolysaccharide. J Cell Physiol (2012) 227:587–95. doi: 10.1002/jcp.22761 21448921

[242] Seo EJ Kim DK Jang IH Choi EJ Shin SH Lee SI. Hypoxia-NOTCH1-SOX2 signaling is important for maintaining cancer stem cells in ovarian cancer. Oncotarget (2016) 7:55624–38. doi: 10.18632/oncotarget.10954 PMC5342441 27489349

[243] Chen X Lan H He D Xu R Zhang Y Cheng Y. Multi-omics profiling identifies risk hypoxia-related signatures for ovarian cancer prognosis. Front Immunol (2021) 12:645839. doi: 10.3389/fimmu.2021.645839 34349753 PMC8327177

[244] Huang Y Zhou Y Zhang M. Identification of seven hypoxia-related genes signature and risk score models for predicting prognosis for ovarian cancer. Funct Integr Genomics (2023) 23:39. doi: 10.1007/s10142-022-00956-3 36642729 PMC9841006

[245] Todeschini P Salviato E Romani C Raimondi V Ciccarese F Ferrari F. Comprehensive profiling of hypoxia-related miRNAs identifies miR-23a-3p overexpression as a marker of platinum resistance and poor prognosis in high-grade serous ovarian cancer. Cancers (2021) 13:3358. doi: 10.3390/cancers13133358 34283087 PMC8268862

[246] Kouidhi S Ben Ayed F Benammar Elgaaied A. Targeting tumor metabolism: a new challenge to improve immunotherapy. Front Immunol (2018) 9:353. doi: 10.3389/fimmu.2018.00353 29527212 PMC5829092

[247] Renner K Singer K Koehl GE Geissler EK Peter K Siska PJ. Metabolic hallmarks of tumor and immune cells in the tumor microenvironment. Front Immunol (2017) 8:248. doi: 10.3389/fimmu.2017.00248 28337200 PMC5340776

[248] Latifi A Luwor RB Bilandzic M Nazaretian S Stenvers K Pyman J. Isolation and characterization of tumor cells from the ascites of ovarian cancer patients: molecular phenotype of chemoresistant ovarian tumors. PloS One (2012) 7:e46858. doi: 10.1371/journal.pone.0046858 23056490 PMC3466197

[249] Bellio C DiGloria C Foster R James K Konstantinopoulos PA Growdon WB. PARP inhibition induces enrichment of DNA repair–proficient CD133 and CD117 positive ovarian cancer stem cells. Mol Cancer Res (2019) 17:431–45. doi: 10.1158/1541-7786.MCR-18-0594 30401718

[250] Kim W-T Ryu CJ. Cancer stem cell surface markers on normal stem cells. BMB Rep (2017) 50:285–98. doi: 10.5483/BMBRep.2017.50.6.039 PMC5498139 28270302

[251] Lapidot T Dar A Kollet O. How do stem cells find their way home? Blood (2005) 106:1901–10. doi: 10.1182/blood-2005-04-1417 15890683

[252] Maleki M Ghanbarvand F Behvarz MR Ejtemaei M Ghadirkhomi E. Comparison of mesenchymal stem cell markers in multiple human adult stem cells. Int J Stem Cells (2014) 7:118–26. doi: 10.15283/ijsc.2014.7.2.118 PMC4249894 25473449

[253] Zuk PA Zhu M Ashjian P De Ugarte DA Huang JI Mizuno H. Human adipose tissue is a source of multipotent stem cells. Mol Biol Cell (2002) 13:4279–95. doi: 10.1091/mbc.e02-02-0105 PMC138633 12475952

[254] Sundberg M Jansson L Ketolainen J Pihlajamäki H Suuronen R Skottman H. CD Marker expression profiles of human embryonic stem cells and their neural derivatives, determined using flow-cytometric analysis, reveal a novel CD marker for exclusion of pluripotent stem cells. Stem Cell Res (2009) 2:113–24. doi: 10.1016/j.scr.2008.08.001 19383417

[255] Zannettino ACW Paton S Arthur A Khor F Itescu S Gimble JM. Multipotential human adipose-derived stromal stem cells exhibit a perivascular phenotype *in vitro* and *in vivo*. J Cell Physiol (2008) 214:413–21. doi: 10.1002/jcp.21210 17654479

[256] Wang F Scoville D He XC Mahe MM Box A Perry JM. Isolation and characterization of intestinal stem cells based on surface marker combinations and colony-formation assay. Gastroenterology (2013) 145:383–395.e21. doi: 10.1053/j.gastro.2013.04.050 23644405 PMC3781924

[257] Clarke MF Fuller M. Stem cells and cancer: two faces of eve. Cell (2006) 124:1111–5. doi: 10.1016/j.cell.2006.03.011 16564000

[258] Dvorak P Dvorakova D Hampl A. Fibroblast growth factor signaling in embryonic and cancer stem cells. FEBS Lett (2006) 580:2869–74. doi: 10.1016/j.febslet.2006.01.095 16516203

[259] Takahashi A Hong L Chefetz I. How to win the ovarian cancer stem cell battle: destroying the roots. Cancer Drug Resist (2021) 3:1021–33. doi: 10.20517/cdr.2020.93 PMC8992559 35582216

[260] Esteller M. Epigenetic gene silencing in cancer: the DNA hypermethylome. Hum Mol Genet (2007) 16:R50–9. doi: 10.1093/hmg/ddm018 17613547

[261] Teodoridis JM Hall J Marsh S Kannall HD Smyth C Curto J. CpG island methylation of DNA damage response genes in advanced ovarian cancer. Cancer Res (2005) 65:8961–7. doi: 10.1158/0008-5472.CAN-05-1187 16204069

[262] Ahmed N Kadife E Raza A Short M Jubinsky PT Kannourakis G. Ovarian cancer, cancer stem cells and current treatment strategies: a potential role of magmas in the current treatment methods. Cells (2020) 9:719. doi: 10.3390/cells9030719 32183385 PMC7140629

[263] Zhang W Klinkebiel D Barger CJ Pandey S Guda C Miller A. Global DNA hypomethylation in epithelial ovarian cancer: passive demethylation and association with genomic instability. Cancers (2020) 12:764. doi: 10.3390/cancers12030764 32213861 PMC7140107

[264] To SKY Mak ASC Eva Fung YM Che C-M Li S-S Deng W. β-catenin downregulates dicer to promote ovarian cancer metastasis. Oncogene (2017) 36:5927–38. doi: 10.1038/onc.2017.185 28650464

[265] Roca Di Gennaro Budillon. Implication for cancer stem cells in solid cancer chemo-resistance: promising therapeutic strategies based on the use of HDAC inhibitors. J Clin Med (2019) 8:912. doi: 10.3390/jcm8070912 31247937 PMC6678716

[266] Annett S Moore G Short A Marshall A McCrudden C Yakkundi A. FKBPL-based peptide, ALM201, targets angiogenesis and cancer stem cells in ovarian cancer. Br J Cancer (2020) 122:361–71. doi: 10.1038/s41416-019-0649-5 PMC7000737 31772325

[267] El Helali A Plummer R Jayson GC Coyle VM Drew Y Mescallado N. A first-in-human phase I dose-escalation trial of the novel therapeutic peptide, ALM201, demonstrates a favourable safety profile in unselected patients with ovarian cancer and other advanced solid tumours. Br J Cancer (2022) 127:92–101. doi: 10.1038/s41416-022-01780-z 35568736 PMC9276671

[268] Coleman RL Broaddus RR Bodurka DC Wolf JK Burke TW Kavanagh JJ. Phase II trial of imatinib mesylate in patients with recurrent platinum- and taxane-resistant epithelial ovarian and primary peritoneal cancers. Gynecol Oncol (2006) 101:126–31. doi: 10.1016/j.ygyno.2005.09.041 16271384

[269] Schilder RJ Sill MW Lee RB Shaw TJ Senterman MK Klein-Szanto AJ. Phase II evaluation of imatinib mesylate in the treatment of recurrent or persistent epithelial ovarian or primary peritoneal carcinoma: a gynecologic oncology group study. J Clin Oncol (2008) 26:3418–25. doi: 10.1200/JCO.2007.14.3420 18612157

[270] Alberts DS Liu PY Wilczynski SP Jang A Moon J Ward JH. Phase II trial of imatinib mesylate in recurrent, biomarker positive, ovarian cancer (Southwest oncology group protocol S0211). Int J Gynecol Cancer (2007) 17:784–8. doi: 10.1111/j.1525-1438.2007.00882.x 17343607

[271] Matei D Emerson RE Schilder J Menning N Baldridge LA Johnson CS. Imatinib mesylate in combination with docetaxel for the treatment of patients with advanced, platinum-resistant ovarian cancer and primary peritoneal carcinomatosis: a Hoosier oncology group trial. Cancer (2008) 113:723–32. doi: 10.1002/cncr.23605 18618737

[272] Lee H-G Shin S-J Chung H-W Kwon S-H Cha S-D Lee J-E. Salinomycin reduces stemness and induces apoptosis on human ovarian cancer stem cell. J Gynecol Oncol (2017) 28:e14. doi: 10.3802/jgo.2017.28.e14 27894167 PMC5323284

[273] Zou M Yin X Zhou X Niu X Wang Y Su M. Salinomycin-loaded high-density lipoprotein exerts promising anti-ovarian cancer effects by inhibiting epithelial–mesenchymal transition. Int J Nanomed (2022) Volume 17:4059–71. doi: 10.2147/IJN.S380598 PMC9467852 36105618

[274] Fan Y Cheng H Liu Y Liu S Lowe S Li Y. Metformin anticancer: reverses tumor hypoxia induced by bevacizumab and reduces the expression of cancer stem cell markers CD44/CD117 in human ovarian cancer SKOV3 cells. Front Pharmacol (2022) 13:955984. doi: 10.3389/fphar.2022.955984 36046821 PMC9421358

[275] Alvero AB Montagna MK Holmberg JC Craveiro V Brown D Mor G. Targeting the mitochondria activates two independent cell death pathways in ovarian cancer stem cells. Mol Cancer Ther (2011) 10:1385–93. doi: 10.1158/1535-7163.MCT-11-0023 PMC3703662 21677151

[276] Grimley E Cole AJ Luong TT McGonigal SC Sinno S Yang D. Aldehyde dehydrogenase inhibitors promote DNA damage in ovarian cancer and synergize with ATM/ATR inhibitors. Theranostics (2021) 11:3540–51. doi: 10.7150/thno.51885 PMC7914353 33664846

[277] Nwani N Condello S Wang Y Swetzig W Barber E Hurley T. A novel ALDH1A1 inhibitor targets cells with stem cell characteristics in ovarian cancer. Cancers (2019) 11:502. doi: 10.3390/cancers11040502 30965686 PMC6521036

[278] Whitworth JM Londoño-Joshi AI Sellers JC Oliver PJ Muccio DD Atigadda VR. The impact of novel retinoids in combination with platinum chemotherapy on ovarian cancer stem cells. Gynecol Oncol (2012) 125:226–30. doi: 10.1016/j.ygyno.2011.12.425 PMC4049085 22155260

[279] Chefetz I Grimley E Yang K Hong L Vinogradova EV Suciu R. A pan-ALDH1A inhibitor induces necroptosis in ovarian cancer stem-like cells. Cell Rep (2019) 26:3061–3075.e6. doi: 10.1016/j.celrep.2019.02.032 30865894 PMC7061440

[280] Guo F Yang Z Sehouli J Kaufmann AM. Blockade of ALDH in cisplatin-resistant ovarian cancer stem cells *In vitro* synergistically enhances chemotherapy-induced cell death. Curr Oncol (2022) 29:2808–22. doi: 10.3390/curroncol29040229 PMC9031660 35448203

[281] Simpkins F Jang K Yoon H Hew KE Kim M Azzam DJ. Dual src and MEK inhibition decreases ovarian cancer growth and targets tumor initiating stem-like cells. Clin Cancer Res (2018) 24:4874–86. doi: 10.1158/1078-0432.CCR-17-3697 PMC6557165 29959144

[282] Wang W Fang F Ozes A Nephew KP. Targeting ovarian cancer stem cells by dual inhibition of HOTAIR and DNA methylation. Mol Cancer Ther (2021) 20:1092–101. doi: 10.1158/1535-7163.MCT-20-0826 PMC8172444 33785648

[283] Choi Y-J Gurunathan S Kim J-H. Graphene oxide–silver nanocomposite enhances cytotoxic and apoptotic potential of salinomycin in human ovarian cancer stem cells (OvCSCs): a novel approach for cancer therapy. Int J Mol Sci (2018) 19:710. doi: 10.3390/ijms19030710 29494563 PMC5877571

[284] Hirst J Pathak HB Hyter S Pessetto ZY Ly T Graw S. Licofelone enhances the efficacy of paclitaxel in ovarian cancer by reversing drug resistance and tumor stem-like properties. Cancer Res (2018) 78:4370–85. doi: 10.1158/0008-5472.CAN-17-3993 PMC6072598 29891506

[285] Brown JR Chan DK Shank JJ Griffith KA Fan H Szulawski R. Phase II clinical trial of metformin as a cancer stem cell-targeting agent in ovarian cancer. JCI Insight (2020) 5:e133247. doi: 10.1172/jci.insight.133247 32369446 PMC7308054

[286] Mi Y Huang Y Deng J. The enhanced delivery of salinomycin to CD133+ ovarian cancer stem cells through CD133 antibody conjugation with poly(lactic-co-glycolic acid)-poly(ethylene glycol) nanoparticles. Oncol Lett (2018) 15:6611–21. doi: 10.3892/ol.2018.8140 PMC5920508 29725407

[287] Skubitz APN Taras EP Boylan KLM Waldron NN Oh S Panoskaltsis-Mortari A. Targeting CD133 in an *in vivo* ovarian cancer model reduces ovarian cancer progression. Gynecol Oncol (2013) 130:579–87. doi: 10.1016/j.ygyno.2013.05.027 PMC3906852 23721800

[288] Klapdor R Wang S Hacker U Büning H Morgan M Dörk T. Improved killing of ovarian cancer stem cells by combining a novel chimeric antigen receptor–based immunotherapy and chemotherapy. Hum Gene Ther (2017) 28:886–96. doi: 10.1089/hum.2017.168 28836469

[289] Ang WX Li Z Chi Z Du S-H Chen C Tay JCK. Intraperitoneal immunotherapy with T cells stably and transiently expressing anti-EpCAM CAR in xenograft models of peritoneal carcinomatosis. Oncotarget (2017) 8:13545–59. doi: 10.18632/oncotarget.14592 PMC5355119 28088790

[290] Berek JS Edwards RP Parker LP DeMars LR Herzog TJ Lentz SS. Catumaxomab for the treatment of malignant ascites in patients with chemotherapy-refractory ovarian cancer: a phase II study. Int J Gynecol Cancer (2014) 24:1583–9. doi: 10.1097/IGC.0000000000000286 25254563

[291] Wimberger P Gilet H Gonschior A-K Heiss MM Moehler M Oskay-Oezcelik G. Deterioration in quality of life (QoL) in patients with malignant ascites: results from a phase II/III study comparing paracentesis plus catumaxomab with paracentesis alone. Ann Oncol (2012) 23:1979–85. doi: 10.1093/annonc/mds178 PMC3403730 22734013

[292] Fossati M Buzzonetti A Monego G Catzola V Scambia G Fattorossi A. Immunological changes in the ascites of cancer patients after intraperitoneal administration of the bispecific antibody catumaxomab (anti-EpCAM×anti-CD3). Gynecol Oncol (2015) 138:343–51. doi: 10.1016/j.ygyno.2015.06.003 26049121

[293] Kim H Xu H George E Hallberg D Kumar S Jagannathan V. Combining PARP with ATR inhibition overcomes PARP inhibitor and platinum resistance in ovarian cancer models. Nat Commun (2020) 11:3726. doi: 10.1038/s41467-020-17127-2 32709856 PMC7381609

[294] Shah PD Wethington SL Pagan C Latif N Tanyi J Martin LP. Combination ATR and PARP inhibitor (CAPRI): a phase 2 study of ceralasertib plus olaparib in patients with recurrent, platinum-resistant epithelial ovarian cancer. Gynecol Oncol (2021) 163:246–53. doi: 10.1016/j.ygyno.2021.08.024 PMC9614917 34620496

[295] Yap TA O’Carrigan B Penney MS Lim JS Brown JS de Miguel Luken MJ. Phase I trial of first-in-Class ATR inhibitor M6620 (VX-970) as monotherapy or in combination with carboplatin in patients with advanced solid tumors. J Clin Oncol (2020) 38:3195–204. doi: 10.1200/JCO.19.02404 PMC7499606 32568634

[296] Kang Y Hu W Ivan C Dalton HJ Miyake T Pecot CV. Role of focal adhesion kinase in regulating YB–1–Mediated paclitaxel resistance in ovarian cancer. JNCI J Natl Cancer Inst (2013) 105:1485–95. doi: 10.1093/jnci/djt210 PMC3787907 24062525

[297] Patel MR Infante JR Moore KN Keegan M Poli A Padval M. Phase 1/1b study of the FAK inhibitor defactinib (VS-6063) in combination with weekly paclitaxel for advanced ovarian cancer. J Clin Oncol (2014) 32:5521–1. doi: 10.1200/jco.2014.32.15_suppl.5521

[298] Diaz Osterman CJ Ozmadenci D Kleinschmidt EG Taylor KN Barrie AM Jiang S. FAK activity sustains intrinsic and acquired ovarian cancer resistance to platinum chemotherapy. eLife (2019) 8:e47327. doi: 10.7554/eLife.47327 31478830 PMC6721800

[299] Fang DD Tao R Wang G Li Y Zhang K Xu C. Discovery of a novel ALK/ROS1/FAK inhibitor, APG-2449, in preclinical non-small cell lung cancer and ovarian cancer models. BMC Cancer (2022) 22:752. doi: 10.1186/s12885-022-09799-4 35820889 PMC9277925

[300] Lee H Kim JW Kim DK Choi DK Lee S Yu JH. Calcium channels as novel therapeutic targets for ovarian cancer stem cells. Int J Mol Sci (2020) 21:2327. doi: 10.3390/ijms21072327 32230901 PMC7177693

[301] Lee H Kim JW Lee D-S Min S-H. Combined poziotinib with manidipine treatment suppresses ovarian cancer stem-cell proliferation and stemness. Int J Mol Sci (2020) 21:7379. doi: 10.3390/ijms21197379 33036254 PMC7583017

[302] Löblein MT Falke I Eich HT Greve B Götte M Troschel FM. Dual knockdown of musashi RNA-binding proteins MSI-1 and MSI-2 attenuates putative cancer stem cell characteristics and therapy resistance in ovarian cancer cells. Int J Mol Sci (2021) 22:11502. doi: 10.3390/ijms222111502 34768932 PMC8584030

[303] He Y Alejo S Venkata PP Johnson JD Loeffel I Pratap UP. Therapeutic targeting of ovarian cancer stem cells using estrogen receptor beta agonist. Int J Mol Sci (2022) 23:7159. doi: 10.3390/ijms23137159 35806169 PMC9266546

[304] Nacarelli T Fukumoto T Zundell JA Fatkhutdinov N Jean S Cadungog MG. NAMPT inhibition suppresses cancer stem-like cells associated with therapy-induced senescence in ovarian cancer. Cancer Res (2020) 80:890–900. doi: 10.1158/0008-5472.CAN-19-2830 31857293 PMC7024650

[305] Yamamoto M Suzuki S Togashi K Sanomachi T Seino S Kitanaka C. AS602801, an anticancer stem cell candidate drug, reduces survivin expression and sensitizes A2780 ovarian cancer stem cells to carboplatin and paclitaxel. Anticancer Res (2018) 38:6699–706. doi: 10.21873/anticanres.13038 30504379

[306] Togashi K Okada M Yamamoto M Suzuki S Sanomachi T Seino S. A small-molecule kinase inhibitor, CEP-1347, inhibits survivin expression and sensitizes ovarian cancer stem cells to paclitaxel. Anticancer Res (2018) 38:4535–42. doi: 10.21873/anticanres.12757 30061219

[307] Moore KN Gunderson CC Sabbatini P McMeekin DS Mantia-Smaldone G Burger RA. A phase 1b dose escalation study of ipafricept (OMP 54F28) in combination with paclitaxel and carboplatin in patients with recurrent platinum-sensitive ovarian cancer. Gynecol Oncol (2019) 154:294–301. doi: 10.1016/j.ygyno.2019.04.001 31174889 PMC7397408

[308] Boone JD Arend RC Johnston BE Cooper SJ Gilchrist SA Oelschlager DK. Targeting the wnt/β-catenin pathway in primary ovarian cancer with the porcupine inhibitor WNT974. Lab Invest (2016) 96:249–59. doi: 10.1038/labinvest.2015.150 26658453

[309] Fischer MM Cancilla B Yeung VP Cattaruzza F Chartier C Murriel CL. WNT antagonists exhibit unique combinatorial antitumor activity with taxanes by potentiating mitotic cell death. Sci Adv (2017) 3:e1700090. doi: 10.1126/sciadv.1700090 28691093 PMC5479655

[310] Kaye SB Fehrenbacher L Holloway R Amit A Karlan B Slomovitz B. Randomized, placebo-controlled study of vismodegib as maintenance therapy in patients with ovarian cancer in second or third complete remission. Clin Cancer Res (2012) 18:6509–18. doi: 10.1158/1078-0432.CCR-12-1796 23032746

[311] Stathis A Hess D von Moos R Homicsko K Griguolo G Joerger M. Phase I trial of the oral smoothened inhibitor sonidegib in combination with paclitaxel in patients with advanced solid tumors. Invest New Drugs (2017) 35:766–72. doi: 10.1007/s10637-017-0454-z 28317088

[312] Pant S Jones SF Kurkjian CD Infante JR Moore KN Burris HA. A first-in-human phase I study of the oral notch inhibitor, LY900009, in patients with advanced cancer. Eur J Cancer (2016) 56:1–9. doi: 10.1016/j.ejca.2015.11.021 26798966

[313] Chen X Gong L Ou R Zheng Z Chen J Xie F. Sequential combination therapy of ovarian cancer with cisplatin and γ-secretase inhibitor MK-0752. Gynecol Oncol (2016) 140:537–44. doi: 10.1016/j.ygyno.2015.12.011 26704638

[314] Diaz-Padilla I Wilson MK Clarke BA Hirte HW Welch SA Mackay HJ. A phase II study of single-agent RO4929097, a gamma-secretase inhibitor of notch signaling, in patients with recurrent platinum-resistant epithelial ovarian cancer: a study of the princess Margaret, Chicago and California phase II consortia. Gynecol Oncol (2015) 137:216–22. doi: 10.1016/j.ygyno.2015.03.005 25769658

[315] Chiorean EG LoRusso P Strother RM Diamond JR Younger A Messersmith WA. A phase I first-in-Human study of enoticumab (REGN421), a fully human delta-like ligand 4 (Dll4) monoclonal antibody in patients with advanced solid tumors. Clin Cancer Res (2015) 21:2695–703. doi: 10.1158/1078-0432.CCR-14-2797 25724527

[316] Huang J Hu W Hu L Previs RA Dalton HJ Yang X-Y. Dll4 inhibition plus aflibercept markedly reduces ovarian tumor growth. Mol Cancer Ther (2016) 15:1344–52. doi: 10.1158/1535-7163.MCT-15-0144 PMC4893925 27009216

[317] Coleman RL Handley KF Burger R Molin GZD Stagg R Sood AK. Demcizumab combined with paclitaxel for platinum-resistant ovarian, primary peritoneal, and fallopian tube cancer: the SIERRA open-label phase ib trial. Gynecol Oncol (2020) 157:386–91. doi: 10.1016/j.ygyno.2020.01.042 32037195

[318] Jimeno A Moore KN Gordon M Chugh R Diamond JR Aljumaily R. A first-in-human phase 1a study of the bispecific anti-DLL4/anti-VEGF antibody navicixizumab (OMP-305B83) in patients with previously treated solid tumors. Invest New Drugs (2019) 37:461–72. doi: 10.1007/s10637-018-0665-y 30229512

[319] Fu S Corr BR Culm-Merdek K Mockbee C Youssoufian H Stagg R. Phase ib study of navicixizumab plus paclitaxel in patients with platinum-resistant ovarian, primary peritoneal, or fallopian tube cancer. J Clin Oncol (2022) 40:2568–77. doi: 10.1200/JCO.21.01801 PMC9362870 35439029

[320] Yu Z Wang Y Wang B Zhai J. Metformin affects paclitaxel sensitivity of ovarian cancer cells through autophagy mediated by long noncoding RNASNHG7/miR-3127-5p axis. Cancer Biother Radiopharm (2022) 37:792–801. doi: 10.1089/cbr.2019.3390 32522016

[321] Bishnu A Sakpal A Ghosh N Choudhury P Chaudhury K Ray P. Long term treatment of metformin impedes development of chemoresistance by regulating cancer stem cell differentiation through taurine generation in ovarian cancer cells. Int J Biochem Cell Biol (2019) 107:116–27. doi: 10.1016/j.biocel.2018.12.016 30593952

[322] Westfall SD Skinner MK. Inhibition of phosphatidylinositol 3-kinase sensitizes ovarian cancer cells to carboplatin and allows adjunct chemotherapy treatment. Mol Cancer Ther (2005) 4:1764–71. doi: 10.1158/1535-7163.MCT-05-0192 16275998

[323] Jones HM Fang Z Sun W Clark LH Stine JE Tran A-Q. Atorvastatin exhibits anti-tumorigenic and anti-metastatic effects in ovarian cancer *in vitro* . Am J Cancer Res (2017) 7:2478–90. PMC5752688 29312801

[324] dos Santos Guimarães I Ladislau-Magescky T Tessarollo NG dos Santos DZ Gimba ERP Sternberg C. Chemosensitizing effects of metformin on cisplatin- and paclitaxel-resistant ovarian cancer cell lines. Pharmacol Rep (2018) 70:409–17. doi: 10.1016/j.pharep.2017.11.007 29627688

[325] Chefetz I Holmberg JC Alvero AB Visintin I Mor G. Inhibition of aurora-a kinase induces cell cycle arrest in epithelial ovarian cancer stem cells by affecting NFĸB pathway. Cell Cycle (2011) 10:2206–14. doi: 10.4161/cc.10.13.16348 PMC3154367 21623171

[326] Michy Massias Bernard Vanwonterghem Henry Guidetti. Verteporfin-loaded lipid nanoparticles improve ovarian cancer photodynamic therapy *In vitro* and *In vivo* . Cancers (2019) 11:1760. doi: 10.3390/cancers11111760 31717427 PMC6896159

[327] Dasari VR Carey DJ Gogoi R. Synergistic enhancement of efficacy of platinum drugs with verteporfin in ovarian cancer cells. BMC Cancer (2020) 20:273. doi: 10.1186/s12885-020-06752-1 32245422 PMC7318501

[328] Han ES Wen W Dellinger TH Wu J Lu SA Jove R. Ruxolitinib synergistically enhances the anti-tumor activity of paclitaxel in human ovarian cancer. Oncotarget (2018) 9:24304–19. doi: 10.18632/oncotarget.24368 PMC5966246 29849942

[329] Burgos-Ojeda D Wu R McLean K Chen Y-C Talpaz M Yoon E. CD24+ ovarian cancer cells are enriched for cancer-initiating cells and dependent on JAK2 signaling for growth and metastasis. Mol Cancer Ther (2015) 14:1717–27. doi: 10.1158/1535-7163.MCT-14-0607 PMC4496272 25969154

[330] Bagratuni T Mavrianou N Gavalas NG Tzannis K Arapinis C Liontos M. JQ1 inhibits tumour growth in combination with cisplatin and suppresses JAK/STAT signalling pathway in ovarian cancer. Eur J Cancer (2020) 126:125–35. doi: 10.1016/j.ejca.2019.11.017 31927213

[331] Wen H Qian M He J Li M Yu Q Leng Z. Inhibiting of self-renewal, migration and invasion of ovarian cancer stem cells by blocking TGF-β pathway. PloS One (2020) 15:e0230230. doi: 10.1371/journal.pone.0230230 32214328 PMC7098562

[332] Fan H Lu X Wang X Liu Y Guo B Zhang Y. Low-dose decitabine-based chemoimmunotherapy for patients with refractory advanced solid tumors: a phase I/II report. J Immunol Res (2014) 2014:1–14. doi: 10.1155/2014/371087 PMC4054619 24963497

[333] Zhang Y Mei Q Liu Y Li X Brock MV Chen M. The safety, efficacy, and treatment outcomes of a combination of low-dose decitabine treatment in patients with recurrent ovarian cancer. OncoImmunology (2017) 6:e1323619. doi: 10.1080/2162402X.2017.1323619 28932630 PMC5599090

[334] Oza AM Matulonis UA Alvarez Secord A Nemunaitis J Roman LD Blagden SP. A randomized phase II trial of epigenetic priming with guadecitabine and carboplatin in platinum-resistant, recurrent ovarian cancer. Clin Cancer Res (2020) 26:1009–16. doi: 10.1158/1078-0432.CCR-19-1638 PMC7056559 31831561

[335] Falchook GS Fu S Naing A Hong DS Hu W Moulder S. Methylation and histone deacetylase inhibition in combination with platinum treatment in patients with advanced malignancies. Invest New Drugs (2013) 31:1192–200. doi: 10.1007/s10637-013-0003-3 PMC3809091 23907406

[336] Wong-Brown MW van der Westhuizen A Bowden NA. Sequential azacitidine and carboplatin induces immune activation in platinum-resistant high-grade serous ovarian cancer cell lines and primes for checkpoint inhibitor immunotherapy. BMC Cancer (2022) 22:100. doi: 10.1186/s12885-022-09197-w 35073851 PMC8787901

[337] Modesitt SC Sill M Hoffman JS Bender DP. A phase II study of vorinostat in the treatment of persistent or recurrent epithelial ovarian or primary peritoneal carcinoma: a gynecologic oncology group study. Gynecol Oncol (2008) 109:182–6. doi: 10.1016/j.ygyno.2008.01.009 18295319

[338] Matulonis U Berlin S Lee H Whalen C Obermayer E Penson R. Phase I study of combination of vorinostat, carboplatin, and gemcitabine in women with recurrent, platinum-sensitive epithelial ovarian, fallopian tube, or peritoneal cancer. Cancer Chemother Pharmacol (2015) 76:417–23. doi: 10.1007/s00280-015-2813-9 26119093

[339] Dizon DS Damstrup L Finkler NJ Lassen U Celano P Glasspool R. Phase II activity of belinostat (PXD-101), carboplatin, and paclitaxel in women with previously treated ovarian cancer. Int J Gynecol Cancer (2012) 22:979–86. doi: 10.1097/IGC.0b013e31825736fd 22694911

[340] Dizon DS Blessing JA Penson RT Drake RD Walker JL Johnston CM. A phase II evaluation of belinostat and carboplatin in the treatment of recurrent or persistent platinum-resistant ovarian, fallopian tube, or primary peritoneal carcinoma: a gynecologic oncology group study. Gynecol Oncol (2012) 125:367–71. doi: 10.1016/j.ygyno.2012.02.019 PMC3330705 22366594

[341] Smith HJ McCaw TR Londono AI Katre AA Meza-Perez S Yang ES. The antitumor effects of entinostat in ovarian cancer require adaptive immunity. Cancer (2018) 124:4657–66. doi: 10.1002/cncr.31761 PMC6294677 30423192

[342] Gupta VG Hirst J Petersen S Roby KF Kusch M Zhou H. Entinostat, a selective HDAC1/2 inhibitor, potentiates the effects of olaparib in homologous recombination proficient ovarian cancer. Gynecol Oncol (2021) 162:163–72. doi: 10.1016/j.ygyno.2021.04.015 PMC8647995 33867143

[343] Hernlund E Hjerpe E Åvall-Lundqvist E Shoshan M. Ovarian carcinoma cells with low levels of β-F1-ATPase are sensitive to combined platinum and 2-deoxy- d -glucose treatment. Mol Cancer Ther (2009) 8:1916–23. doi: 10.1158/1535-7163.MCT-09-0179 19567816

[344] Bellio C DiGloria C Spriggs DR Foster R Growdon WB Rueda BR. The metabolic inhibitor CPI-613 negates treatment enrichment of ovarian cancer stem cells. Cancers (2019) 11:1678. doi: 10.3390/cancers11111678 31671803 PMC6896080

[345] Ventura R Mordec K Waszczuk J Wang Z Lai J Fridlib M. Inhibition of *de novo* palmitate synthesis by fatty acid synthase induces apoptosis in tumor cells by remodeling cell membranes, inhibiting signaling pathways, and reprogramming gene expression. EBioMedicine (2015) 2:808–24. doi: 10.1016/j.ebiom.2015.06.020 PMC4563160 26425687

[346] Han C Yang L Choi HH Baddour J Achreja A Liu Y. Amplification of USP13 drives ovarian cancer metabolism. Nat Commun (2016) 7:13525. doi: 10.1038/ncomms13525 27892457 PMC5133706

[347] Sawyer BT Qamar L Yamamoto TM McMellen A Watson ZL Richer JK. Targeting fatty acid oxidation to promote anoikis and inhibit ovarian cancer progression. Mol Cancer Res (2020) 18:1088–98. doi: 10.1158/1541-7786.MCR-19-1057 PMC7335321 32198139

[348] Zhu J Wu G Song L Cao L Tan Z Tang M. NKX2-8 deletion-induced reprogramming of fatty acid metabolism confers chemoresistance in epithelial ovarian cancer. EBioMedicine (2019) 43:238–52. doi: 10.1016/j.ebiom.2019.04.041 PMC6562195 31047858

[349] Tesfay L Paul BT Konstorum A Deng Z Cox AO Lee J. Stearoyl-CoA desaturase 1 protects ovarian cancer cells from ferroptotic cell death. Cancer Res (2019) 79:5355–66. doi: 10.1158/0008-5472.CAN-19-0369 PMC6801059 31270077

[350] Dasari S Fang Y Mitra AK. Cancer associated fibroblasts: naughty neighbors that drive ovarian cancer progression. Cancers (2018) 10:406. doi: 10.3390/cancers10110406 30380628 PMC6265896

[351] Hussain A Voisin V Poon S Karamboulas C Bui NHB Meens J. Distinct fibroblast functional states drive clinical outcomes in ovarian cancer and are regulated by TCF21. J Exp Med (2020) 217:e20191094. doi: 10.1084/jem.20191094 32434219 PMC7398174

[352] Cummings M Freer C Orsi NM. Targeting the tumour microenvironment in platinum-resistant ovarian cancer. Semin Cancer Biol (2021) 77:3–28. doi: 10.1016/j.semcancer.2021.02.007 33607246

[353] Sandoval P Jiménez-Heffernan JA Rynne-Vidal Á Pérez-Lozano ML Gilsanz Á Ruiz-Carpio V. Carcinoma-associated fibroblasts derive from mesothelial cells via mesothelial-to-mesenchymal transition in peritoneal metastasis. J Pathol (2013) 231:517–31. doi: 10.1002/path.4281 24114721

[354] Mikuła-Pietrasik J Uruski P Pakuła M Maksin K Szubert S Woźniak A. Oxidative stress contributes to hepatocyte growth factor-dependent pro-senescence activity of ovarian cancer cells. Free Radic Biol Med (2017) 110:270–9. doi: 10.1016/j.freeradbiomed.2017.06.015 28652056

[355] Pakuła M Witucka A Uruski P Radziemski A Moszyński R Szpurek D. Senescence-related deterioration of intercellular junctions in the peritoneal mesothelium promotes the transmesothelial invasion of ovarian cancer cells. Sci Rep (2019) 9:7587. doi: 10.1038/s41598-019-44123-4 31110245 PMC6527686

[356] Rynne-Vidal A Au-Yeung CL Jiménez-Heffernan JA Pérez-Lozano ML Cremades-Jimeno L Bárcena C. Mesothelial-to-mesenchymal transition as a possible therapeutic target in peritoneal metastasis of ovarian cancer: mesothelial-to-mesenchymal transition and peritoneal metastatic niche. J Pathol (2017) 242:140–51. doi: 10.1002/path.4889 PMC5468005 28247413

[357] Nakamura K Sawada K Kinose Y Yoshimura A Toda A Nakatsuka E. Exosomes promote ovarian cancer cell invasion through transfer of CD44 to peritoneal mesothelial cells. Mol Cancer Res (2017) 15:78–92. doi: 10.1158/1541-7786.MCR-16-0191 27758876

[358] Gao L Nie X Gou R Hu Y Dong H Li X. Exosomal ANXA2 derived from ovarian cancer cells regulates epithelial-mesenchymal plasticity of human peritoneal mesothelial cells. J Cell Mol Med (2021) 25:10916–29. doi: 10.1111/jcmm.16983 PMC8642686 34725902

[359] Fujikake K Kajiyama H Yoshihara M Nishino K Yoshikawa N Utsumi F. A novel mechanism of neovascularization in peritoneal dissemination via cancer-associated mesothelial cells affected by TGF-β derived from ovarian cancer. Oncol Rep (2017) 39:193–200. doi: 10.3892/or.2017.6104 29192324

[360] Siu MKY Jiang Y Wang J Leung THY Ngu SF Cheung ANY. PDK1 promotes ovarian cancer metastasis by modulating tumor-mesothelial adhesion, invasion, and angiogenesis via α5β1 integrin and JNK/IL-8 signaling. Oncogenesis (2020) 9:24. doi: 10.1038/s41389-020-0209-0 32071289 PMC7028730

[361] Yasui H Kajiyama H Tamauchi S Suzuki S Peng Y Yoshikawa N. CCL2 secreted from cancer-associated mesothelial cells promotes peritoneal metastasis of ovarian cancer cells through the P38-MAPK pathway. Clin Exp Metastasis (2020) 37:145–58. doi: 10.1007/s10585-019-09993-y 31541326

[362] Au-Yeung C-L Yeung T-L Achreja A Zhao H Yip K-P Kwan S-Y. ITLN1 modulates invasive potential and metabolic reprogramming of ovarian cancer cells in omental microenvironment. Nat Commun (2020) 11:3546. doi: 10.1038/s41467-020-17383-2 32669559 PMC7363861

[363] Qian J LeSavage BL Hubka KM Ma C Natarajan S Eggold JT. Cancer-associated mesothelial cells promote ovarian cancer chemoresistance through paracrine osteopontin signaling. J Clin Invest (2021) 131:e146186. doi: 10.1172/JCI146186 34396988 PMC8363279

[364] Carroll MJ Fogg KC Patel HA Krause HB Mancha A-S Patankar MS. Alternatively-activated macrophages upregulate mesothelial expression of p-selectin to enhance adhesion of ovarian cancer cells. Cancer Res (2018) 78:3560–73. doi: 10.1158/0008-5472.CAN-17-3341 PMC6030435 29739756

[365] Asem M Young AM Oyama C Claure de la Zerda A Liu Y Yang J. Host Wnt5a potentiates microenvironmental regulation of ovarian cancer metastasis. Cancer Res (2020) 80:1156–70. doi: 10.1158/0008-5472.CAN-19-1601 PMC8245162 31932454

[366] Jia D Lu M Jung KH Park JH Yu L Onuchic JN. Elucidating cancer metabolic plasticity by coupling gene regulation with metabolic pathways. Proc Natl Acad Sci (2019) 116:3909–18. doi: 10.1073/pnas.1816391116 PMC6397570 30733294

[367] Desbats MA Giacomini I Prayer-Galetti T Montopoli M. Metabolic plasticity in chemotherapy resistance. Front Oncol (2020) 10:281. doi: 10.3389/fonc.2020.00281 32211323 PMC7068907

[368] Pastò A Pagotto A Pilotto G De Paoli A De Salvo GL Baldoni A. Resistance to glucose starvation as metabolic trait of platinum-resistant human epithelial ovarian cancer cells. Oncotarget (2017) 8:6433–45. doi: 10.18632/oncotarget.14118 PMC5351643 28031535

[369] Zhao G Cardenas H Matei D. Ovarian cancer–why lipids matter. Cancers (2019) 11:1870. doi: 10.3390/cancers11121870 31769430 PMC6966536

[370] Ladanyi A Mukherjee A Kenny HA Johnson A Mitra AK Sundaresan S. Adipocyte-induced CD36 expression drives ovarian cancer progression and metastasis. Oncogene (2018) 37:2285–301. doi: 10.1038/s41388-017-0093-z PMC5920730 29398710

[371] Ahmed AA Lu Z Jennings NB Etemadmoghadam D Capalbo L Jacamo RO. SIK2 is a centrosome kinase required for bipolar mitotic spindle formation that provides a potential target for therapy in ovarian cancer. Cancer Cell (2010) 18:109–21. doi: 10.1016/j.ccr.2010.06.018 PMC3954541 20708153

[372] Bauerschlag DO Maass N Leonhardt P Verburg FA Pecks U Zeppernick F. Fatty acid synthase overexpression: target for therapy and reversal of chemoresistance in ovarian cancer. J Transl Med (2015) 13:146. doi: 10.1186/s12967-015-0511-3 25947066 PMC4504229

[373] Yi M Li J Chen S Cai J Ban Y Peng Q. Emerging role of lipid metabolism alterations in cancer stem cells. J Exp Clin Cancer Res (2018) 37:118. doi: 10.1186/s13046-018-0784-5 29907133 PMC6003041

[374] Borzdziłowska P Bednarek I. Alpha mangostin and cisplatin as modulators of exosomal interaction of ovarian cancer cell with fibroblasts. Int J Mol Sci (2022) 23:8913. doi: 10.3390/ijms23168913 36012171 PMC9408324

[375] Cheng L Wu S Zhang K Qing Y Xu T. A comprehensive overview of exosomes in ovarian cancer: emerging biomarkers and therapeutic strategies. J Ovarian Res (2017) 10:73. doi: 10.1186/s13048-017-0368-6 29100532 PMC5670635

[376] Keller S König A-K Marmé F Runz S Wolterink S Koensgen D. Systemic presence and tumor-growth promoting effect of ovarian carcinoma released exosomes. Cancer Lett (2009) 278:73–81. doi: 10.1016/j.canlet.2008.12.028 19188015

[377] Shenoy GN Loyall J Berenson CS Kelleher RJ Iyer V Balu-Iyer SV. Sialic acid–dependent inhibition of T cells by exosomal ganglioside GD3 in ovarian tumor microenvironments. J Immunol (2018) 201:3750–8. doi: 10.4049/jimmunol.1801041 PMC6289713 30446565

[378] Bretz NP Ridinger J Rupp A-K Rimbach K Keller S Rupp C. Body fluid exosomes promote secretion of inflammatory cytokines in monocytic cells via toll-like receptor signaling. J Biol Chem (2013) 288:36691–702. doi: 10.1074/jbc.M113.512806 PMC3868779 24225954

[379] Labani-Motlagh A Israelsson P Ottander U Lundin E Nagaev I Nagaeva O. Differential expression of ligands for NKG2D and DNAM-1 receptors by epithelial ovarian cancer-derived exosomes and its influence on NK cell cytotoxicity. Tumor Biol (2016) 37:5455–66. doi: 10.1007/s13277-015-4313-2 26563374

[380] Yi H Ye J Yang X-M Zhang L-W Zhang Z-G Chen Y-P. High-grade ovarian cancer secreting effective exosomes in tumor angiogenesis. Int J Clin Exp Pathol (2015) 8:5062–70. PMC4503072 26191200

[381] Tang MKS Yue PYK Ip PP Huang R-L Lai H-C Cheung ANY. Soluble e-cadherin promotes tumor angiogenesis and localizes to exosome surface. Nat Commun (2018) 9:2270. doi: 10.1038/s41467-018-04695-7 29891938 PMC5995921

[382] Mo Y Leung LL Mak CSL Wang X Chan W-S Hui LMN. Tumor-secreted exosomal miR-141 activates tumor-stroma interactions and controls premetastatic niche formation in ovarian cancer metastasis. Mol Cancer (2023) 22:4. doi: 10.1186/s12943-022-01703-9 36624516 PMC9827705

[383] Kanlikilicer P Bayraktar R Denizli M Rashed MH Ivan C Aslan B. Exosomal miRNA confers chemo resistance via targeting Cav1/p-gp/M2-type macrophage axis in ovarian cancer. eBioMedicine (2018) 38:100–12. doi: 10.1016/j.ebiom.2018.11.004 PMC6306310 30487062

[384] Ying X Wu Q Wu X Zhu Q Wang X Jiang L. Epithelial ovarian cancer-secreted exosomal miR-222-3p induces polarization of tumor-associated macrophages. Oncotarget (2016) 7:43076–87. doi: 10.18632/oncotarget.9246 PMC5190009 27172798

[385] Yoshimura A Sawada K Nakamura K Kinose Y Nakatsuka E Kobayashi M. Exosomal miR-99a-5p is elevated in sera of ovarian cancer patients and promotes cancer cell invasion by increasing fibronectin and vitronectin expression in neighboring peritoneal mesothelial cells. BMC Cancer (2018) 18:1065. doi: 10.1186/s12885-018-4974-5 30396333 PMC6217763

[386] Kobayashi M Salomon C Tapia J Illanes SE Mitchell MD Rice GE. Ovarian cancer cell invasiveness is associated with discordant exosomal sequestration of let-7 miRNA and miR-200. J Transl Med (2014) 12:4. doi: 10.1186/1479-5876-12-4 24393345 PMC3896684

[387] Zamaraev AV Volik PI Sukhikh GT Kopeina GS Zhivotovsky B. Long non-coding RNAs: a view to kill ovarian cancer. Biochim Biophys Acta BBA Rev Cancer (2021) 1876:188584. doi: 10.1016/j.bbcan.2021.188584 34157315

[388] Taylor SE Chan DK Yang D Bruno T Lieberman R Siddiqui J. Shifting the soil: metformin treatment decreases the protumorigenic tumor microenvironment in epithelial ovarian cancer. Cancers (2022) 14:2298. doi: 10.3390/cancers14092298 35565427 PMC9104826

[389] Tan L Tondo-Steele K Foster C McIlwain C Bolland DE Crawford HC. Inhibition of tumor microenvironment cytokine signaling sensitizes ovarian cancer cells to antiestrogen therapy. Cancers (2022) 14:4675. doi: 10.3390/cancers14194675 36230597 PMC9564160

[390] Pulaski HL Spahlinger G Silva IA McLean K Kueck AS Reynolds RK. Identifying alemtuzumab as an anti-myeloid cell antiangiogenic therapy for the treatment of ovarian cancer. J Transl Med (2009) 7:49. doi: 10.1186/1479-5876-7-49 19545375 PMC2704183

[391] Martin LP Sill M Shahin MS Powell M DiSilvestro P Landrum LM. A phase II evaluation of AMG 102 (rilotumumab) in the treatment of persistent or recurrent epithelial ovarian, fallopian tube or primary peritoneal carcinoma: a gynecologic oncology group study. Gynecol Oncol (2014) 132:526–30. doi: 10.1016/j.ygyno.2013.12.018 PMC4469031 24361733

[392] Berek J Taylor P McGuire W Smith LM Schultes B Nicodemus CF. Oregovomab maintenance monoimmunotherapy does not improve outcomes in advanced ovarian cancer. J Clin Oncol (2009) 27:418–25. doi: 10.1200/JCO.2008.17.8400 19075271

[393] Battaglia A Buzzonetti A Fossati M Scambia G Fattorossi A Madiyalakan MR. Translational immune correlates of indirect antibody immunization in a randomized phase II study using scheduled combination therapy with carboplatin/paclitaxel plus oregovomab in ovarian cancer patients. Cancer Immunol Immunother (2020) 69:383–97. doi: 10.1007/s00262-019-02456-z PMC11027811 31897661

[394] Brewer M Angioli R Scambia G Lorusso D Terranova C Panici PB. Front-line chemo-immunotherapy with carboplatin-paclitaxel using oregovomab indirect immunization in advanced ovarian cancer: a randomized phase II study. Gynecol Oncol (2020) 156:523–9. doi: 10.1016/j.ygyno.2019.12.024 31916979

[395] Crawford A Haber L Kelly MP Vazzana K Canova L Ram P. A mucin 16 bispecific T cell–engaging antibody for the treatment of ovarian cancer. Sci Transl Med (2019) 11:eaau7534. doi: 10.1126/scitranslmed.aau7534 31217340

[396] Santin AD Vergote I González-Martín A Moore K Oaknin A Romero I. Safety and activity of anti-mesothelin antibody–drug conjugate anetumab ravtansine in combination with pegylated-liposomal doxorubicin in platinum-resistant ovarian cancer: multicenter, phase ib dose escalation and expansion study. Int J Gynecol Cancer (2023) 33:562–70. doi: 10.1136/ijgc-2022-003927 PMC10086500 36564099

[397] Mullamitha SA Ton NC Parker GJM Jackson A Julyan PJ Roberts C. Phase I evaluation of a fully human anti–αv integrin monoclonal antibody (CNTO 95) in patients with advanced solid tumors. Clin Cancer Res (2007) 13:2128–35. doi: 10.1158/1078-0432.CCR-06-2779 17404096

[398] Bell-McGuinn KM Matthews CM Ho SN Barve M Gilbert L Penson RT. Single-arm study of the anti-α5β1 integrin antibody volociximab as monotherapy in patients with platinum-resistant advanced epithelial ovarian or primary peritoneal cancer. Gynecol Oncol (2011) 121:273–9. doi: 10.1016/j.ygyno.2010.12.362 PMC4426879 21276608

[399] Barkal AA Brewer RE Markovic M Kowarsky M Barkal SA Zaro BW. CD24 signalling through macrophage siglec-10 is a target for cancer immunotherapy. Nature (2019) 572:392–6. doi: 10.1038/s41586-019-1456-0 PMC6697206 31367043

[400] Sikic BI Lakhani N Patnaik A Shah SA Chandana SR Rasco D. First-in-Human, first-in-Class phase I trial of the anti-CD47 antibody Hu5F9-G4 in patients with advanced cancers. J Clin Oncol (2019) 37:946–53. doi: 10.1200/JCO.18.02018 PMC7186585 30811285

[401] Gupta R Cristea M Frankel P Ruel C Chen C Wang Y. Randomized trial of oral cyclophosphamide versus oral cyclophosphamide with celecoxib for recurrent epithelial ovarian, fallopian tube, and primary peritoneal cancer. Cancer Treat Res Commun (2019) 21:100155. doi: 10.1016/j.ctarc.2019.100155 31279962 PMC9018111

[402] Legge F Paglia A D’Asta M Fuoco G Scambia G Ferrandina G. Phase II study of the combination carboplatin plus celecoxib in heavily pre-treated recurrent ovarian cancer patients. BMC Cancer (2011) 11:214. doi: 10.1186/1471-2407-11-214 21627839 PMC3123659

[403] Reyners AKL de Munck L Erdkamp FLG Smit WM Hoekman K Lalisang RI. A randomized phase II study investigating the addition of the specific COX-2 inhibitor celecoxib to docetaxel plus carboplatin as first-line chemotherapy for stage IC to IV epithelial ovarian cancer, fallopian tube or primary peritoneal carcinomas: the DoCaCel study. Ann Oncol (2012) 23:2896–902. doi: 10.1093/annonc/mds107 22689176

[404] Penn CA Yang K Zong H Lim J-Y Cole A Yang D. Therapeutic impact of nanoparticle therapy targeting tumor-associated macrophages. Mol Cancer Ther (2018) 17:96–106. doi: 10.1158/1535-7163.MCT-17-0688 29133618 PMC5752569

[405] Robinson-Smith TM Isaacsohn I Mercer CA Zhou M Van Rooijen N Husseinzadeh N. Macrophages mediate inflammation-enhanced metastasis of ovarian tumors in mice. Cancer Res (2007) 67:5708–16. doi: 10.1158/0008-5472.CAN-06-4375 17575137

[406] Germano G Frapolli R Belgiovine C Anselmo A Pesce S Liguori M. Role of macrophage targeting in the antitumor activity of trabectedin. Cancer Cell (2013) 23:249–62. doi: 10.1016/j.ccr.2013.01.008 23410977

[407] Casado A Callata HR Manzano A Marquina G Alonso T Gajate P. Trabectedin for reversing platinum resistance and resensitization to platinum in patients with recurrent ovarian cancer. Future Oncol (2019) 15:271–80. doi: 10.2217/fon-2018-0554 30465613

[408] Takahashi S Takekuma M Tamura K Takehara K Nomura H Ono M. A phase I study of combined trabectedin and pegylated liposomal doxorubicin therapy for advanced relapsed ovarian cancer. Int J Clin Oncol (2021) 26:1977–85. doi: 10.1007/s10147-021-01973-1 PMC8449774 34189636

[409] Monk BJ Herzog TJ Wang G Triantos S Maul S Knoblauch R. A phase 3 randomized, open-label, multicenter trial for safety and efficacy of combined trabectedin and pegylated liposomal doxorubicin therapy for recurrent ovarian cancer. Gynecol Oncol (2020) 156:535–44. doi: 10.1016/j.ygyno.2019.12.043 31924332

[410] Toulmonde M Brahmi M Giraud A Chakiba C Bessede A Kind M. Trabectedin plus durvalumab in patients with advanced pretreated soft tissue sarcoma and ovarian carcinoma (TRAMUNE): an open-label, multicenter phase ib study. Clin Cancer Res (2022) 28:1765–72. doi: 10.1158/1078-0432.CCR-21-2258 34965951

[411] Colombo N Zaccarelli E Baldoni A Frezzini S Scambia G Palluzzi E. Multicenter, randomised, open-label, non-comparative phase 2 trial on the efficacy and safety of the combination of bevacizumab and trabectedin with or without carboplatin in women with partially platinum-sensitive recurrent ovarian cancer. Br J Cancer (2019) 121:744–50. doi: 10.1038/s41416-019-0584-5 PMC6888836 31537908

[412] Moughon DL He H Schokrpur S Jiang ZK Yaqoob M David J. Macrophage blockade using CSF1R inhibitors reverses the vascular leakage underlying malignant ascites in late-stage epithelial ovarian cancer. Cancer Res (2015) 75:4742–52. doi: 10.1158/0008-5472.CAN-14-3373 PMC4675660 26471360

[413] Johnson M Dudek AZ Sukari A Call J Kunk PR Lewis K. ARRY-382 in combination with pembrolizumab in patients with advanced solid tumors: results from a phase 1b/2 study. Clin Cancer Res (2022) 28:2517–26. doi: 10.1158/1078-0432.CCR-21-3009 PMC9359741 35302585

[414] Lyons YA Pradeep S Wu SY Haemmerle M Hansen JM Wagner MJ. Macrophage depletion through colony stimulating factor 1 receptor pathway blockade overcomes adaptive resistance to anti-VEGF therapy. Oncotarget (2017) 8:96496–505. doi: 10.18632/oncotarget.20410 PMC5722500 29228548

[415] Zhang F Parayath NN Ene CI Stephan SB Koehne AL Coon ME. Genetic programming of macrophages to perform anti-tumor functions using targeted mRNA nanocarriers. Nat Commun (2019) 10:3974. doi: 10.1038/s41467-019-11911-5 31481662 PMC6722139

[416] Tebbe C Chhina J Dar SA Sarigiannis K Giri S Munkarah AR. Metformin limits the adipocyte tumor-promoting effect on ovarian cancer. Oncotarget (2014) 5:4746–64. doi: 10.18632/oncotarget.2012 PMC4148096 24970804

[417] Yamamura S Matsumura N Mandai M Huang Z Oura T Baba T. The activated transforming growth factor-beta signaling pathway in peritoneal metastases is a potential therapeutic target in ovarian cancer. Int J Cancer (2012) 130:20–8. doi: 10.1002/ijc.25961 21503873

[418] Gao Y Shan N Zhao C Wang Y Xu F Li J. LY2109761 enhances cisplatin antitumor activity in ovarian cancer cells. Int J Clin Exp Pathol (2015) 8:4923–32. PMC4503057 26191185

[419] Matei D Sill MW Lankes HA DeGeest K Bristow RE Mutch D. Activity of sorafenib in recurrent ovarian cancer and primary peritoneal carcinomatosis: a gynecologic oncology group trial. J Clin Oncol (2011) 29:69–75. doi: 10.1200/JCO.2009.26.7856 21098323 PMC3055861

[420] Lee J-M Annunziata CM Hays JL Cao L Choyke P Yu M. Phase II trial of bevacizumab and sorafenib in recurrent ovarian cancer patients with or without prior-bevacizumab treatment. Gynecol Oncol (2020) 159:88–94. doi: 10.1016/j.ygyno.2020.07.031 32747013 PMC7541580

[421] Ramasubbaiah R Perkins SM Schilder J Whalen C Johnson CS Callahan M. Sorafenib in combination with weekly topotecan in recurrent ovarian cancer, a phase I/II study of the Hoosier oncology group. Gynecol Oncol (2011) 123:499–504. doi: 10.1016/j.ygyno.2011.08.033 21955480

[422] Chekerov R Hilpert F Mahner S El-Balat A Harter P De Gregorio N. Sorafenib plus topotecan versus placebo plus topotecan for platinum-resistant ovarian cancer (TRIAS): a multicentre, randomised, double-blind, placebo-controlled, phase 2 trial. Lancet Oncol (2018) 19:1247–58. doi: 10.1016/S1470-2045(18)30372-3 30100379

[423] Welch SA Hirte HW Elit L Schilder RJ Wang L MacAlpine K. Sorafenib in combination with gemcitabine in recurrent epithelial ovarian cancer: a study of the princess Margaret hospital phase II consortium. Int J Gynecol Cancer (2010) 20:787–93. doi: 10.1111/IGC.0b013e3181e273a8 20847613

[424] Hainsworth JD Thompson DS Bismayer JA Gian VG Merritt WM Whorf RC. Paclitaxel/carboplatin with or without sorafenib in the first-line treatment of patients with stage III/IV epithelial ovarian cancer: a randomized phase II study of the Sarah cannon research institute. Cancer Med (2015) 4:673–81. doi: 10.1002/cam4.376 PMC4430260 25556916

[425] Colombo N Mangili G Mammoliti S Kalling M Tholander B Sternas L. A phase II study of aflibercept in patients with advanced epithelial ovarian cancer and symptomatic malignant ascites. Gynecol Oncol (2012) 125:42–7. doi: 10.1016/j.ygyno.2011.11.021 22112608

[426] Matulonis UA Berlin S Ivy P Tyburski K Krasner C Zarwan C. Cediranib, an oral inhibitor of vascular endothelial growth factor receptor kinases, is an active drug in recurrent epithelial ovarian, fallopian tube, and peritoneal cancer. J Clin Oncol (2009) 27:5601–6. doi: 10.1200/JCO.2009.23.2777 PMC2792954 19826113

[427] Hirte H Lheureux S Fleming GF Sugimoto A Morgan R Biagi J. A phase 2 study of cediranib in recurrent or persistent ovarian, peritoneal or fallopian tube cancer: a trial of the princess Margaret, Chicago and California phase II consortia. Gynecol Oncol (2015) 138:55–61. doi: 10.1016/j.ygyno.2015.04.009 25895616

[428] Lheureux S Oaknin A Garg S Bruce JP Madariaga A Dhani NC. EVOLVE: a multicenter open-label single-arm clinical and translational phase II trial of cediranib plus olaparib for ovarian cancer after PARP inhibition progression. Clin Cancer Res (2020) 26:4206–15. doi: 10.1158/1078-0432.CCR-19-4121 32444417

[429] Liu JF Brady MF Matulonis UA Miller A Kohn EC Swisher EM. Olaparib with or without cediranib versus platinum-based chemotherapy in recurrent platinum-sensitive ovarian cancer (NRG-GY004): a randomized, open-label, phase III trial. J Clin Oncol (2022) 40:2138–47. doi: 10.1200/JCO.21.02011 PMC9242406 35290101

[430] Lee J-M Moore RG Ghamande S Park MS Diaz JP Chapman J. Cediranib in combination with olaparib in patients without a germline BRCA1/2 mutation and with recurrent platinum-resistant ovarian cancer: phase IIb CONCERTO trial. Clin Cancer Res (2022) 28:4186–93. doi: 10.1158/1078-0432.CCR-21-1733 PMC9527502 35917514

[431] Ledermann JA Hackshaw A Kaye S Jayson G Gabra H McNeish I. Randomized phase II placebo-controlled trial of maintenance therapy using the oral triple angiokinase inhibitor BIBF 1120 after chemotherapy for relapsed ovarian cancer. J Clin Oncol (2011) 29:3798–804. doi: 10.1200/JCO.2010.33.5208 21859991

[432] du Bois A Kristensen G Ray-Coquard I Reuss A Pignata S Colombo N. Standard first-line chemotherapy with or without nintedanib for advanced ovarian cancer (AGO-OVAR 12): a randomised, double-blind, placebo-controlled phase 3 trial. Lancet Oncol (2016) 17:78–89. doi: 10.1016/S1470-2045(15)00366-6 26590673

[433] Pignata S Lorusso D Scambia G Sambataro D Tamberi S Cinieri S. MITO-11: a randomized multicenter phase II trial testing the addition of pazopanib to weekly paclitaxel in platinum-resistant or -refractory advanced ovarian cancer (AOC). J Clin Oncol (2014) 32:5503–3. doi: 10.1200/jco.2014.32.15_suppl.5503

[434] Joly F Fabbro M Berton D Lequesne J Anota A Puszkiel A. Paclitaxel with or without pazopanib for ovarian cancer relapsing during bevacizumab maintenance therapy: the GINECO randomized phase II TAPAZ study. Gynecol Oncol (2022) 166:389–96. doi: 10.1016/j.ygyno.2022.06.022 35902297

[435] Dinkic C Eichbaum M Schmidt M Grischke EM Gebauer G Fricke HC. Pazopanib (GW786034) and cyclophosphamide in patients with platinum-resistant, recurrent, pre-treated ovarian cancer - results of the PACOVAR-trial. Gynecol Oncol (2017) 146:279–84. doi: 10.1016/j.ygyno.2017.05.013 28528917

[436] Zhang Y Fang H Wang X Wang H Pan G Chen J. The efficacy and safety of pazopanib plus chemotherapy in treating recurrent or persistent ovarian cancer:: a systematic review and meta-analysis. Am J Clin Oncol (2023) 46:254–62. doi: 10.1097/COC.0000000000000999 PMC10205118 36877187

[437] Yeung T-L Sheng J Leung CS Li F Kim J Ho SY. Systematic identification of druggable epithelial–stromal crosstalk signaling networks in ovarian cancer. JNCI J Natl Cancer Inst (2019) 111:272–82. doi: 10.1093/jnci/djy097 PMC6410941 29860390

[438] Zhao Y Cao J Melamed A Worley M Gockley A Jones D. Losartan treatment enhances chemotherapy efficacy and reduces ascites in ovarian cancer models by normalizing the tumor stroma. Proc Natl Acad Sci (2019) 116:2210–9. doi: 10.1073/pnas.1818357116 PMC6369817 30659155

[439] Moore KN Bookman M Sehouli J Miller A Anderson C Scambia G. Atezolizumab, bevacizumab, and chemotherapy for newly diagnosed stage III or IV ovarian cancer: placebo-controlled randomized phase III trial (IMagyn050/GOG 3015/ENGOT-OV39). J Clin Oncol (2021) 39:1842–55. doi: 10.1200/JCO.21.00306 PMC8189598 33891472

[440] Moroney JW Powderly J Lieu CH Bendell JC Eckhardt SG Chang C-W. Safety and clinical activity of atezolizumab plus bevacizumab in patients with ovarian cancer: a phase ib study. Clin Cancer Res (2020) 26:5631–7. doi: 10.1158/1078-0432.CCR-20-0477 32723836

[441] Monk BJ Colombo N Oza AM Fujiwara K Birrer MJ Randall L. Chemotherapy with or without avelumab followed by avelumab maintenance versus chemotherapy alone in patients with previously untreated epithelial ovarian cancer (JAVELIN ovarian 100): an open-label, randomised, phase 3 trial. Lancet Oncol (2021) 22:1275–89. doi: 10.1016/S1470-2045(21)00342-9 34363762

[442] Pujade-Lauraine E Fujiwara K Ledermann JA Oza AM Kristeleit R Ray-Coquard I-L. Avelumab alone or in combination with chemotherapy versus chemotherapy alone in platinum-resistant or platinum-refractory ovarian cancer (JAVELIN ovarian 200): an open-label, three-arm, randomised, phase 3 study. Lancet Oncol (2021) 22:1034–46. doi: 10.1016/S1470-2045(21)00216-3 34143970

[443] Schram AM Colombo N Arrowsmith E Narayan V Yonemori K Scambia G. Avelumab plus talazoparib in patients with *BRCA1/2* - or *ATM* -altered advanced solid tumors: results from JAVELIN BRCA/ATM, an open-label, multicenter, phase 2b, tumor-agnostic trial. JAMA Oncol (2023) 9:29. doi: 10.1001/jamaoncol.2022.5218 36394867 PMC9673021

[444] Zamarin D Walderich S Holland A Zhou Q Iasonos AE Torrisi JM. Safety, immunogenicity, and clinical efficacy of durvalumab in combination with folate receptor alpha vaccine TPIV200 in patients with advanced ovarian cancer: a phase II trial. J Immunother Cancer (2020) 8:e000829. doi: 10.1136/jitc-2020-000829 32503949 PMC7279674

[445] Lampert EJ Zimmer A Padget M Cimino-Mathews A Nair JR Liu Y. Combination of PARP inhibitor olaparib, and PD-L1 inhibitor durvalumab, in recurrent ovarian cancer: a proof-of-Concept phase II study. Clin Cancer Res (2020) 26:4268–79. doi: 10.1158/1078-0432.CCR-20-0056 PMC7442720 32398324

[446] Lee J-Y Kim B-G Kim J-W Lee JB Park E Joung J-G. On behalf of Korean gynecologic oncology group (KGOG) investigators. biomarker-guided targeted therapy in platinum-resistant ovarian cancer (AMBITION; KGOG 3045): a multicentre, open-label, five-arm, uncontrolled, umbrella trial. J Gynecol Oncol (2022) 33:e45. doi: 10.3802/jgo.2022.33.e45 35320892 PMC9250853

[447] Zimmer AS Nichols E Cimino-Mathews A Peer C Cao L Lee M-J. A phase I study of the PD-L1 inhibitor, durvalumab, in combination with a PARP inhibitor, olaparib, and a VEGFR1–3 inhibitor, cediranib, in recurrent women’s cancers with biomarker analyses. J Immunother Cancer (2019) 7:197. doi: 10.1186/s40425-019-0680-3 31345267 PMC6657373

[448] Zamarin D Burger RA Sill MW Powell DJ Lankes HA Feldman MD. Randomized phase II trial of nivolumab versus nivolumab and ipilimumab for recurrent or persistent ovarian cancer: an NRG oncology study. J Clin Oncol (2020) 38:1814–23. doi: 10.1200/JCO.19.02059 PMC7255977 32275468

[449] Liu JF Herold C Gray KP Penson RT Horowitz N Konstantinopoulos PA. Assessment of combined nivolumab and bevacizumab in relapsed ovarian cancer: a phase 2 clinical trial. JAMA Oncol (2019) 5:1731. doi: 10.1001/jamaoncol.2019.3343 31600397 PMC6802049

[450] Hamanishi J Takeshima N Katsumata N Ushijima K Kimura T Takeuchi S. Nivolumab versus gemcitabine or pegylated liposomal doxorubicin for patients with platinum-resistant ovarian cancer: open-label, randomized trial in Japan (NINJA). J Clin Oncol (2021) 39:3671–81. doi: 10.1200/JCO.21.00334 PMC8601279 34473544

[451] Manning-Geist BL Gnjatic S Aghajanian C Konner J Kim SH Sarasohn D. Phase I study of a multivalent WT1 peptide vaccine (Galinpepimut-s) in combination with nivolumab in patients with WT1-expressing ovarian cancer in second or third remission. Cancers (2023) 15:1458. doi: 10.3390/cancers15051458 36900251 PMC10001251

[452] Zsiros E Lynam S Attwood KM Wang C Chilakapati S Gomez EC. Efficacy and safety of pembrolizumab in combination with bevacizumab and oral metronomic cyclophosphamide in the treatment of recurrent ovarian cancer: a phase 2 nonrandomized clinical trial. JAMA Oncol (2021) 7:78. doi: 10.1001/jamaoncol.2020.5945 33211063 PMC7677872

[453] Matulonis UA Shapira-Frommer R Santin AD Lisyanskaya AS Pignata S Vergote I. Antitumor activity and safety of pembrolizumab in patients with advanced recurrent ovarian cancer: results from the phase II KEYNOTE-100 study. Ann Oncol (2019) 30:1080–7. doi: 10.1093/annonc/mdz135 31046082

[454] Walsh CS Kamrava M Rogatko A Kim S Li A Cass I. Phase II trial of cisplatin, gemcitabine and pembrolizumab for platinum-resistant ovarian cancer. PloS One (2021) 16:e0252665. doi: 10.1371/journal.pone.0252665 34081738 PMC8174738

[455] Konstantinopoulos PA Waggoner S Vidal GA Mita M Moroney JW Holloway R. Single-arm phases 1 and 2 trial of niraparib in combination with pembrolizumab in patients with recurrent platinum-resistant ovarian carcinoma. JAMA Oncol (2019) 5:1141. doi: 10.1001/jamaoncol.2019.1048 31194228 PMC6567832

[456] Liao JB Gwin WR Urban RR Hitchcock-Bernhardt KM Coveler AL Higgins DM. Pembrolizumab with low-dose carboplatin for recurrent platinum-resistant ovarian, fallopian tube, and primary peritoneal cancer: survival and immune correlates. J Immunother Cancer (2021) 9:e003122. doi: 10.1136/jitc-2021-003122 34531249 PMC8449961

[457] Lee EK Xiong N Cheng S-C Barry WT Penson RT Konstantinopoulos PA. Combined pembrolizumab and pegylated liposomal doxorubicin in platinum resistant ovarian cancer: a phase 2 clinical trial. Gynecol Oncol (2020) 159:72–8. doi: 10.1016/j.ygyno.2020.07.028 32771276

[458] Wang J Wang C Li Y Li M Zhu T Shen Z. Potential of peptide-engineered exosomes with overexpressed miR-92b-3p in anti-angiogenic therapy of ovarian cancer. Clin Transl Med (2021) 11:e425. doi: 10.1002/ctm2.425 34047469 PMC8131502

[459] Kobayashi M Sawada K Miyamoto M Shimizu A Yamamoto M Kinose Y. Exploring the potential of engineered exosomes as delivery systems for tumor-suppressor microRNA replacement therapy in ovarian cancer. Biochem Biophys Res Commun (2020) 527:153–61. doi: 10.1016/j.bbrc.2020.04.076 32446360

[460] Liu H Shen M Zhao D Ru D Duan Y Ding C. The effect of triptolide-loaded exosomes on the proliferation and apoptosis of human ovarian cancer SKOV3 cells. BioMed Res Int (2019) 2019:1–14. doi: 10.1155/2019/2595801 PMC6556367 31240207

[461] Li L He D Guo Q Zhang Z Ru D Wang L. Exosome-liposome hybrid nanoparticle codelivery of TP and miR497 conspicuously overcomes chemoresistant ovarian cancer. J Nanobiotechnol (2022) 20:50. doi: 10.1186/s12951-022-01264-5 PMC8787930 35078498

[462] Zheng Y Li Z Yang S Wang Y Luan Z. CircEXOC6B suppresses the proliferation and motility and sensitizes ovarian cancer cells to paclitaxel through miR-376c-3p/FOXO3 axis. Cancer Biother Radiopharm (2022) 37:802–14. doi: 10.1089/cbr.2020.3739 33006481

[463] Peng D Wu T Wang J Huang J Zheng L Wang P. microRNA-671-5p reduces tumorigenicity of ovarian cancer via suppressing HDAC5 and HIF-1α expression. Chem Biol Interact (2022) 355:109780. doi: 10.1016/j.cbi.2021.109780 34990588

[464] Yuan D Guo T Qian H Ge H Zhao Y Huang A. Icariside II suppresses the tumorigenesis and development of ovarian cancer by regulating miR-144-3p/IGF2R axis. Drug Dev Res (2022) 83:1383–93. doi: 10.1002/ddr.21967 35808943

[465] Rao Y Zhang W Li D Li X Ma Y Qu P. Circ TYMP1 inhibits carcinogenesis and cisplatin resistance in ovarian cancer by reducing Smad2/3 phosphorylation via a MicroRNA-182A-3p/TGF1B axis. Contrast Media Mol Imaging (2022) 2022:1–9. doi: 10.1155/2022/1032557 PMC9398837 36072623

[466] Yang X Wang J Li H Sun Y Tong X. Downregulation of hsa_circ_0026123 suppresses ovarian cancer cell metastasis and proliferation through the miR−124−3p/EZH2 signaling pathway. Int J Mol Med (2020) 47:668–76. doi: 10.3892/ijmm.2020.4804 PMC7797451 33416105

[467] Korzun T Moses AS Kim J Patel S Schumann C Levasseur PR. Nanoparticle-based follistatin messenger RNA therapy for reprogramming metastatic ovarian cancer and ameliorating cancer-associated cachexia. Small (2022) 18:2204436. doi: 10.1002/smll.202204436 PMC9633376 36098251

[468] Kim GH Won JE Byeon Y Kim MG Wi TI Lee JM. Selective delivery of PLXDC1 small interfering RNA to endothelial cells for anti-angiogenesis tumor therapy using CD44-targeted chitosan nanoparticles for epithelial ovarian cancer. Drug Delivery (2018) 25:1394–402. doi: 10.1080/10717544.2018.1480672 PMC6096458 29890852

[469] Vescarelli E Gerini G Megiorni F Anastasiadou E Pontecorvi P Solito L. MiR-200c sensitizes olaparib-resistant ovarian cancer cells by targeting neuropilin 1. J Exp Clin Cancer Res (2020) 39:3. doi: 10.1186/s13046-019-1490-7 31898520 PMC6939329

[470] Wilczyński JR Wilczyński M Paradowska E. Cancer stem cells in ovarian cancer–a source of tumor success and a challenging target for novel therapies. Int J Mol Sci (2022) 23:2496. doi: 10.3390/ijms23052496 35269636 PMC8910575

[471] Köbel M Kang EY. The evolution of ovarian carcinoma subclassification. Cancers (2022) 14:416. doi: 10.3390/cancers14020416 35053578 PMC8774015

[472] Lupia M Melocchi V Bizzaro F Lo Riso P Dama E Baronio M. Integrated molecular profiling of patient-derived ovarian cancer models identifies clinically relevant signatures and tumor vulnerabilities. Int J Cancer (2022) 151:240–54. doi: 10.1002/ijc.33983 PMC9310611 35218560

[473] Liu X Wu A Wang X Liu Y Xu Y Liu G. Identification of metabolism-associated molecular subtype in ovarian cancer. J Cell Mol Med (2021) 25:9617–26. doi: 10.1111/jcmm.16907 PMC8505839 34523782

[474] Fang F Cardenas H Huang H Jiang G Perkins SM Zhang C. Genomic and epigenomic signatures in ovarian cancer associated with resensitization to platinum drugs. Cancer Res (2018) 78:631–44. doi: 10.1158/0008-5472.CAN-17-1492 PMC5811373 29229600

[475] Givel A-M Kieffer Y Scholer-Dahirel A Sirven P Cardon M Pelon F. miR200-regulated CXCL12β promotes fibroblast heterogeneity and immunosuppression in ovarian cancers. Nat Commun (2018) 9:1056. doi: 10.1038/s41467-018-03348-z 29535360 PMC5849633

[476] Li Y Tian R Liu J Li J Tan H Wu Q. Deciphering the immune landscape dominated by cancer-associated fibroblasts to investigate their potential in indicating prognosis and guiding therapeutic regimens in high grade serous ovarian carcinoma. Front Immunol (2022) 13:940801. doi: 10.3389/fimmu.2022.940801 36119108 PMC9478207

[477] The Cancer Genome Atlas Research Network. Integrated genomic analyses of ovarian carcinoma. Nature (2011) 474:609–15. doi: 10.1038/nature10166 PMC3163504 21720365

[478] Irani S. Emerging insights into the biology of metastasis: a review article. Iran J Basic Med Sci (2019) 22:833–47. doi: 10.22038/ijbms.2019.32786.7839 PMC6760483 31579438

[479] Konecny GE Wang C Hamidi H Winterhoff B Kalli KR Dering J. Prognostic and therapeutic relevance of molecular subtypes in high-grade serous ovarian cancer. JNCI J Natl Cancer Inst (2014) 106:dju249. doi: 10.1093/jnci/dju249 25269487 PMC4271115

[480] Liu B Ji X Li J Zhu N Long J Zhuang X. Integrative analysis identifies three molecular subsets in ovarian cancer. Clin Transl Med (2022) 12:e1029. doi: 10.1002/ctm2.1029 36116137 PMC9482804

[481] Ledermann JA Embleton AC Raja F Perren TJ Jayson GC Rustin GJS. Cediranib in patients with relapsed platinum-sensitive ovarian cancer (ICON6): a randomised, double-blind, placebo-controlled phase 3 trial. Lancet (2016) 387:1066–74. doi: 10.1016/S0140-6736(15)01167-8 27025186

[482] Li D Marchenko ND Moll UM. SAHA shows preferential cytotoxicity in mutant p53 cancer cells by destabilizing mutant p53 through inhibition of the HDAC6-Hsp90 chaperone axis. Cell Death Differ (2011) 18:1904–13. doi: 10.1038/cdd.2011.71 PMC3170683 21637290

[483] Hao Q Li J Zhang Q Xu F Xie B Lu H. Single-cell transcriptomes reveal heterogeneity of high-grade serous ovarian carcinoma. Clin Transl Med (2021) 11:e500. doi: 10.1002/ctm2.500 34459128 PMC8335963

[484] Zhang X Yang Q. An immune-related lncRNA pairing model for predicting the prognosis and immune-infiltrating cell condition in human ovarian cancer. BioMed Res Int (2022) 2022:1–17. doi: 10.1155/2022/3168408 PMC9400430 36033566

[485] Gong G Lin T Yuan Y. Integrated analysis of gene expression and DNA methylation profiles in ovarian cancer. J Ovarian Res (2020) 13:30. doi: 10.1186/s13048-020-00632-9 32192517 PMC7082962

[486] Lu X Ji C Jiang L Zhu Y Zhou Y Meng J. Tumour microenvironment-based molecular profiling reveals ideal candidates for high-grade serous ovarian cancer immunotherapy. Cell Prolif (2021) 54:e12979. doi: 10.1111/cpr.12979 33522069 PMC7941229

[487] Patch A-M Christie EL Etemadmoghadam D Garsed DW George J Fereday S. Whole–genome characterization of chemoresistant ovarian cancer. Nature (2015) 521:489–94. doi: 10.1038/nature14410 26017449

[488] Lin KK Harrell MI Oza AM Oaknin A Ray-Coquard I Tinker AV. *BRCA* reversion mutations in circulating tumor DNA predict primary and acquired resistance to the PARP inhibitor rucaparib in high-grade ovarian carcinoma. Cancer Discovery (2019) 9:210–9. doi: 10.1158/2159-8290.CD-18-0715 30425037

[489] Kondrashova O Nguyen M Shield-Artin K Tinker AV Teng NNH Harrell MI. Secondary somatic mutations restoring *RAD51C* and *RAD51D* associated with acquired resistance to the PARP inhibitor rucaparib in high-grade ovarian carcinoma. Cancer Discovery (2017) 7:984–98. doi: 10.1158/2159-8290.CD-17-0419 PMC5612362 28588062

[490] Li Y Zhang X Gao Y Shang C Yu B Wang T. Development of a genomic signatures-based predictor of initial platinum-resistance in advanced high-grade serous ovarian cancer patients. Front Oncol (2021) 10:625866. doi: 10.3389/fonc.2020.625866 33747898 PMC7977004

[491] Qi X Yu C Wang Y Lin Y Shen B. Network vulnerability-based and knowledge-guided identification of microRNA biomarkers indicating platinum resistance in high-grade serous ovarian cancer. Clin Transl Med (2019) 8:28. doi: 10.1186/s40169-019-0245-6 31664600 PMC6820656

[492] Martincuks A Song J Kohut A Zhang C Li Y-J Zhao Q. PARP inhibition activates STAT3 in both tumor and immune cells underlying therapy resistance and immunosuppression in ovarian cancer. Front Oncol (2021) 11:724104. doi: 10.3389/fonc.2021.724104 34956861 PMC8693573

[493] Lee AH Mejia Peña C Dawson MR. Comparing the secretomes of chemorefractory and chemoresistant ovarian cancer cell populations. Cancers (2022) 14:1418. doi: 10.3390/cancers14061418 35326569 PMC8946241

[494] Zhu X Shen H Yin X Yang M Wei H Chen Q. Macrophages derived exosomes deliver miR-223 to epithelial ovarian cancer cells to elicit a chemoresistant phenotype. J Exp Clin Cancer Res (2019) 38:81. doi: 10.1186/s13046-019-1095-1 30770776 PMC6377760

[495] Zhang K Erkan EP Jamalzadeh S Dai J Andersson N Kaipio K. Longitudinal single-cell RNA-seq analysis reveals stress-promoted chemoresistance in metastatic ovarian cancer. Sci Adv (2022) 8:eabm1831. doi: 10.1126/sciadv.abm1831 35196078 PMC8865800

[496] Kan T Zhang S Zhou S Zhang Y Zhao Y Gao Y. Single-cell RNA-seq recognized the initiator of epithelial ovarian cancer recurrence. Oncogene (2022) 41:895–906. doi: 10.1038/s41388-021-02139-z 34992217

[497] Banerjee S Tian T Wei Z Shih N Feldman MD Alwine JC. The ovarian cancer oncobiome. Oncotarget (2017) 8:36225–45. doi: 10.18632/oncotarget.16717 PMC5482651 28410234

[498] Kania KD Haręża D Wilczyński JR Wilczyński M Jarych D Malinowski A. The toll-like receptor 4 polymorphism Asp299Gly is associated with an increased risk of ovarian cancer. Cells (2022) 11:3137. doi: 10.3390/cells11193137 36231099 PMC9563956

[499] Haręża DA Wilczyński JR Paradowska E. Human papillomaviruses as infectious agents in gynecological cancers. oncogenic properties of viral proteins. Int J Mol Sci (2022) 23:1818. doi: 10.3390/ijms23031818 35163748 PMC8836588

[500] Morand S Devanaboyina M Staats H Stanbery L Nemunaitis J. Ovarian cancer immunotherapy and personalized medicine. Int J Mol Sci (2021) 22:6532. doi: 10.3390/ijms22126532 34207103 PMC8234871

[501] Bonifácio VDB. Ovarian cancer biomarkers: moving forward in early detection. In: Serpa J, editor. Tumor microenvironment. advances in experimental medicine and biology. Cham: Springer International Publishing (2020). p. 355–63. doi: 10.1007/978-3-030-34025-4_18 32130708

[502] Zhu JW Charkhchi P Akbari MR. Potential clinical utility of liquid biopsies in ovarian cancer. Mol Cancer (2022) 21:114. doi: 10.1186/s12943-022-01588-8 35545786 PMC9092780

[503] Zhang R Siu MKY Ngan HYS Chan KKL. Molecular biomarkers for the early detection of ovarian cancer. Int J Mol Sci (2022) 23:12041. doi: 10.3390/ijms231912041 36233339 PMC9569881

